# Structural equation modeling the “control of gut overgrowth” in the prevention of ICU-acquired Gram-negative infection

**DOI:** 10.1186/s13054-020-02906-6

**Published:** 2020-05-04

**Authors:** James C. Hurley

**Affiliations:** 1 Melbourne Medical School, University of Melbourne, Melbourne, Australia; 2 Internal Medicine Service, Ballarat Health Services, PO Box 577, Ballarat, Victoria 3353 Australia

**Keywords:** Bacteremia, *Acinetobacter*, *Pseudomonas*, Antibiotic prophylaxis, Study design, Intensive care, Mechanical ventilation, Selective digestive decontamination, Polymyxin generalized structural equation model

## Abstract

**Background:**

Conceptually, the “control of gut overgrowth” (COGO) is key in mediating prevention against infection with Gram-negative bacilli by topical antibiotic prophylaxis, a common constituent of selective digestive decontamination (SDD) regimens. However, the relative importance of the other SDD components, enteral and protocolized parenteral antibiotic prophylaxis, versus other methods of infection prevention and versus other contextual exposures cannot be resolved within individual studies.

**Methods:**

Seven candidate generalized structural equation models founded on COGO concepts were confronted with *Pseudomonas* and *Acinetobacter* bacteremia as well as ventilator-associated pneumonia data derived from > 200 infection prevention studies. The following group-level exposures were included in the models: use and mode of antibiotic prophylaxis, anti-septic and non-decontamination methods of infection prevention; proportion receiving mechanical ventilation; trauma ICU; mean length of ICU stay; and concurrency versus non-concurrency of topical antibiotic prophylaxis study control groups.

**Results:**

In modeling *Pseudomonas* and *Acinetobacter* gut overgrowth as latent variables, anti-septic interventions had the strongest negative effect against *Pseudomonas* gut overgrowth but no intervention was significantly negative against *Acinetobacter* gut overgrowth. Strikingly, protocolized parenteral antibiotic prophylaxis and concurrency each have positive effects in the model, enteral antibiotic prophylaxis is neutral, and *Acinetobacter* bacteremia incidences are high within topical antibiotic prophylaxis studies, moreso with protocolized parenteral antibiotic prophylaxis exposure. Paradoxically, topical antibiotic prophylaxis (moreso with protocolized parenteral antibiotic prophylaxis) appears to provide the strongest summary prevention effects against overall bacteremia and overall VAP.

**Conclusions:**

Structural equation modeling of published Gram-negative bacillus infection data enables a test of the COGO concept. Paradoxically, *Acinetobacter* and *Pseudomonas* bacteremia incidences are unusually high among studies of topical antibiotic prophylaxis.

## Take home message

- TAP-based decontamination regimens appear superior versus other methods at reducing incidences of overall VAP and bacteremia infections among ICU patients.
- Structural equation modeling of published *Pseudomonas* and *Acinetobacter* infection data enables a test of the control of gut overgrowth concept in the mediation of TAP-based decontamination.
- Paradoxically, both *Acinetobacter* and *Pseudomonas* bacteremia incidences are unusually high among studies of TAP.

## Tweet

GSEM modeling of *Pseudomonas* and *Acinetobacter* gut overgrowth demonstrates complex and paradoxical relationships within SDD/SOD studies.

## Introduction

Of three broad categories of infection prevention in the ICU patient group, selective oral decontamination/selective digestive decontamination (SOD/SDD) shows superior apparent benefit towards overall infection prevention within the ICU context versus anti-septic-based and non-decontamination-based prevention methods [1–9].

The control of gut overgrowth (COGO) is one mechanism proposed to explain how SOD/SDD regimens might prevent ICU-acquired infection. In general, the antibiotics constituent within SOD/SDD regimens, such as topical polymyxin and aminoglycosides, specifically target Gram-negative bacilli including *Pseudomonas* and *Acinetobacter* bacteria whereas anti-septic- and non-decontamination-based prevention methods do not [10].

The exact mechanism for how each of these methods prevents ICU-acquired infection, the basis for the apparent superiority of SOD/SDD among these methods, and even the optimal locus for decontamination, whether the gut or elsewhere, remains unclear despite > 200 studies among patients requiring prolonged mechanical ventilation (MV) or ICU stay [11]. Moreover, the relative importance of the individual SDD components, topical (TAP), enteral (EAP), and protocolized parenteral antibiotic prophylaxis (PPAP; not contained within SOD regimens), versus other methods of infection prevention and versus other contextual exposures such as length of stay and being in a trauma ICU context remains unclear. In addition, concurrency, being the concurrent mixing of study and control patients within the ICU, as typically occurs within randomized concurrent control studies, is believed to influence the results of SOD/SDD studies versus studies without concurrency (i.e., concurrent versus non-concurrent control; CC versus NCC) [10, 12].

The objectives here are threefold. Firstly, to recapitulate the evidence for overall ventilator-associated pneumonia (VAP) and bacteremia prevention among the three broad categories of infection prevention for which *Pseudomonas* and *Acinetobacter* infection data is available. Secondly, to develop and confront candidate models founded on COGO concepts using *Pseudomonas* and *Acinetobacter* infection data from these studies as well as studies without an intervention using GSEM modeling. Thirdly, to compare the relative impacts of the various group-level exposures and interventions on *Pseudomonas* and *Acinetobacter* gut overgrowth as latent variables within the optimal GSEM model.

## Materials and methods

Being an analysis of published work, ethics committee review of this study was not required.

### Study selection and decant of groups

The literature search and study decant used here (Fig S1; see Electronic Supplementary Material for additional ESM tables, ESM figures, and ESM references) is in six steps which is described in full in the ESM and as described previously [13].

Of note, studies undertaken in the context of an ICU outbreak [14–16] were excluded. Due to the absence of eligible studies of TAP undertaken in Asia and Central and South America, together with the significant worldwide variation in both *Pseudomonas* [17] and moreso *Acinetobacter*-associated VAP [18], studies from these regions were excluded from this analysis. A snowballing search strategy [19] using the “Related articles” function within Google Scholar was undertaken for additional studies not identified within systematic reviews.

All eligible studies were then collated, and any duplicate studies were removed and streamed into groups of patients from studies with or without an infection prevention intervention. Those studies without a study intervention provide observational groups.

The component groups were decanted from each study as either observational, control, or intervention groups. Within studies of TAP, any group receiving TAP in any formulation was regarded as an intervention group and all other groups were regarded as a control group regardless of other interventions. The control groups from studies of TAP were stratified into NCC and CC groups.

### Outcomes of interest

The incidences of overall *Pseudomonas* and *Acinetobacter* VAP as well as the incidences of overall *Pseudomonas* and *Acinetobacter* bacteremia were extracted. These were each expressed as a proportion using the number of patients with prolonged (> 24 h) stay in the ICU as the denominator. *Pseudomonas* and *Acinetobacter* gut overgrowth are latent variables as defined within the GSEM models (see below).

### Exposures of interest

The following were also extracted where available: the proportion of each group receiving MV, the proportion of admissions for trauma, and the mean length of ICU stay (LOS). An anti-septic exposure included agents such as chlorhexidine, povidone-iodine, and iseganan. All anti-septic exposures were included regardless of whether the application was to the oropharynx, by tooth-brushing or by body wash.

TAP is defined here as the application of topical antibiotic (TA) prophylaxis to the oropharynx without regard to the specific TA constituents nor to concomitant EAP, being the enteral applications of TA, or PPAP. Note that SOD generally consists of only TAP whereas SDD typically involves TAP together with both EAP and PPAP. A control group of an SOD/SDD study was classified as a CC control if the group was concurrent within the same ICU at the same time as intervention group patients were receiving TAP.

### Visual benchmarking

Scatter plots of the overall and *Pseudomonas* and *Acinetobacter* VAP and bacteremia incidence data were generated to facilitate a visual survey of the entire data as derived from the literature. To facilitate this visual survey, a benchmark for each outcome of interest was generated from the groups of the observational studies using the “metan” command as described in the ESM. The caterpillar plots [20] illustrating the derivation of each bacteremia benchmark are shown in the supplementary material.

### Structural equation modeling

Seven candidate GSEM models were developed using *Pseudomonas* and *Acinetobacter* gut overgrowth as the central latent variables. Group exposure or not to the following factors served as binary indicator variables towards these two latent variables: non-decontamination-based prevention methods, anti-septic-based prevention methods, TAP-based prevention methods, exposure to PPAP, membership of a CC control group within a TAP intervention study, whether the majority of the group were trauma patients, whether more than 90% of patients of the group received more than 24 h of MV, and whether the mean (or median) length of ICU stay for the group was 7 days or more.

The VAP and bacteremia count data for each of *Pseudomonas* and *Acinetobacter* using the number of observed patients as the denominator served as the measurement component for the latent variables using a logit link function in each GSEM. In each model, the observations were clustered by a study identifier in order to generate a robust variance covariance matrix of the parameters of each coefficient estimate. The various exogenous variables were entered into each model without any preselection step to sequentially develop the seven candidate GSEM models using the “GSEM” command in Stata [21]. The model with the lowest Akaike’s information criterion (AIC) score was selected as having parsimony and optimal fit from among the seven candidate models.

### Availability of data and materials

All data generated or analyzed during this study are included in this published article and its supplementary information files (see ESM).

## Results

### Characteristics of the studies

Of the 214 studies identified by the search, 130 were sourced from 23 systematic reviews. Others were found during previous searches or by snowball sampling [19] (Fig S1). Most studies were published between 1990 and 2010, and most had a mean ICU LOS exceeding 7 days. A minority originated from either North American or trauma ICUs. Twenty-one studies had either no control group or more than one control or intervention group. The majority of groups from studies of infection prevention interventions had less than 150 patients per group versus more than 150 patients in the observational studies.

Among the various types of TAP regimen, either topical polymyxin or topical aminoglycoside or both were contained in every regimen except two. PPAP, being a cephalosporin in every case except two, was used within eight control groups and 29 intervention groups of TAP studies. Among TAP intervention groups, 23 used TAP alone (i.e., SOD regimens) and 29 used TAP, EAP, and PPAP in combination (i.e., SDD regimens).

### Overall infection prevention effect

The summary effect sizes for the three categories of interventions against overall VAP (Fig S2 - S4) and also against overall bacteremia (Fig S5-S7) incidence are presented as caterpillar plots. The TAP-based interventions provided greater apparent protection against VAP versus the two other intervention categories (Table 1). Of note, the TAP studies which did (i.e., SDD regimens) versus did not (i.e., SOD regimens) include PPAP within the intervention demonstrated greater protection against both overall bacteremia and overall VAP (Table 1).

**Table 1:** Characteristics of studies

	Observational studies	Infection prevention studies		
	No intervention	Non-decontamination	Anti-septic	TAP ± PPAP/EAP
Study characteristics				
Sources	Table S1	Table S2	Table S3	Table S4
Number of studies^a^	111	45	13	48
Origin from systematic review^b^	46	38	7	38
North American ICUs^c^	32	10	6	3
LOS > 7 days	88	37	9	37
MV for > 48 h for < 90%^d^	21	1	5	11
Trauma ICUs^e^	22	8	2	14
PPAP use in the control group^f^	0	0	1	8
Study publication year (range)	1987–2014	1987–2017	2000–2018	1984–2018
Group characteristics				
Numbers of patients per control group (median; IQR)^g^	279 135–707	75 61–161	96 36–217	86 31–128
Prevention effect size (odds ratio; 95% CI; number of studies)				
VAP	NA	0.73; 0.66–0.80 (45) (see Fig S2)	0.89; 0.72–1.11 (10) (see Fig S3)	0.38; 0.33–0.44 (37)^h^ (see Fig S4)
Bacteremia	NA	0.99; 0.71–1.39 (6) (see Fig S5)	0.72; 0.66–0.79 (10) (see Fig S6)	0.69; 0.62–0.76 (33)^i^ (see Fig S7)

^a^Several studies had more than one control and/or intervention group. Hence, the number of groups does not equal the number of studies

^b^Studies that were sourced from 16 systematic reviews (references in web-only supplementary)

^c^Study originating from an ICU in Canada of the USA

^d^Studies for which less than 90% of patients were reported to receive > 48 h of MV

^e^Trauma ICU arbitrarily defined as an ICU with more than 50% of admissions for trauma

^f^Use of PPAP for control group patients. PPAP is protocolized parenteral antibiotic prophylaxis

^g^Data is median and inter-quartile range (IQR)

^h^VAP prevention effect size for studies not including versus including PPAP in the antibiotic intervention was 0.44; 0.36–0.55 (*n* = 13) and 0.34; 0.28–0.41 (*n* = 24), respectively (see Fig S4)

^i^Bacteremia prevention effect size for studies not including versus including PPAP in the antibiotic intervention was 0.77; 0.68–0.88 (*n* = 10) and 0.57; 0.48–0.67 (*n* = 22), respectively (see Fig S7)

### GSEM modeling

**Fig. 1:**
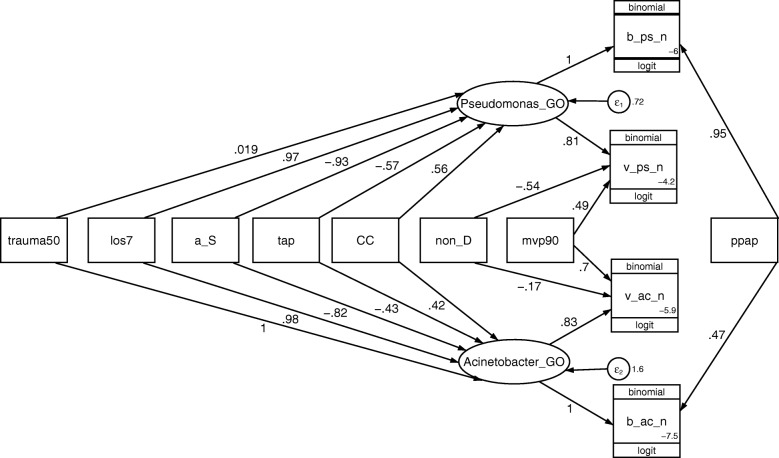
The optimal GSEM (model 6) founded on COGO concepts in relation to *Pseudomonas* and *Acinetobacter* infection data. *Pseudomonas* GO and *Acinetobacter* GO (ovals) are latent variables representing *Pseudomonas* and *Acinetobacter* gut overgrowth (GO), respectively. The variables in rectangles are binary predictor variables representing the group-level exposure to the following: a trauma ICU setting (trauma50), mean or median length of ICU stay ≥ 7 days (los7), exposure to a topical anti-septic-based prevention method (a_S), exposure to a TAP-based prevention method (tap), concurrency of a control group with a TAP intervention group (CC), exposure to a non-decontamination-based prevention method (non-D), use of mechanical ventilation for more than 90% of the group (mvp90), or exposure to PPAP (ppap). The circles contain error terms. The three-part boxes represent the count data for *Pseudomonas* and *Acinetobacter* VAP (v_ps_n, v_ac_n) and bacteremia (b_ps_n, b_ac_n). These counts are logit transformed with the total number of patients in each group as the denominator using the logit link function in the generalized model of the GSEM. Note that EAP use is confounded by PPAP use and that EAP use when separately entered into model 7 (ESM Fig S14) was non-significant

Seven candidate GSEM models of the relationship between various group-level exposures on *Pseudomonas* and *Acinetobacter* gut overgrowth as latent variables were evaluated for fit and parsimony (see Table 2; Fig S8–S14). The optimal model (model 6) is shown (Fig. 1). In developing the seven candidate GSEM models, exposures to PPAP and non-decontamination interventions on *Pseudomonas* and *Acinetobacter* gut overgrowth were both associated with weak coefficients and these pathways were dropped from model 2 onwards. EAP was not a significant factor, and its introduction failed to improve the model fit (model 7, Fig S14).

**Table 2:** Development of the GSEM model

	Model 1	Model 2	Model 3	Model 4	Model 5	Model 7	Model 6	
	Fig S8	Fig S9	Fig S10	Fig S11	Fig S12	Fig S14	Fig S13	95% CI
**Factor**^a,b,c,d,e,f,g,h,i^								
**b_Ps_n**								
***Pseudomonas*** gut overgrowth	1	1	1	1	1	1	1	Constrained
**ppap**			1.11**	0.97**	0.97**	1.00**	0.95**	0.27 to 1.61
**_cons**	− 5.18***	− 5.19***	− 5.38***	− 6.00***	− 6.00***	− 6.05***	− 6.05***	− 6.6 to − 5.4
**b_Ac_n**								
**Acinetobacter GO**	1	1	1	1	1	1	1	Constrained
**ppap**			0.6	0.46	0.48	0.44	0.47	− 0.51 to 4639
**_cons**	− 6.74***	− 6.74***	− 6.83***	− 7.38***	− 7.44***	− 7.47***	− 7.47***	− 8.0 to − 7.0
**v_Ps_n**								
**Pseudomonas** gut overgrowth	0.67***	0.67***	0.71***	0.80***	0.80***	0.81***	0.81***	0.51 to 1.09
**mvp90**	0.55*	0.54*	0.49*	0.43	0.43	0.48*	0.49*	0.03 to 0.92
**non_D**	− 0.37*	− 0.58***	− 0.61***	− 0.60***	− 0.60***	− 0.54***	− 0.54***	− 0.79 to − 0.31
**_cons**	− 3.63***	− 3.63***	− 3.56***	− 4.17***	− 4.17***	− 4.24***	−4.25***	− 4.7 to − 3.7
**v_Ac_n**								
**Acinetobacter** gut overgrowth	0.73***	0.73***	0.74***	0.83***	0.83***	0.83***	0.83***	+ 0.66 to 1.01
**mvp90**	0.79*	0.79*	0.73	0.71	0.69	0.71	0.7	− 0.12 to 1.55
**non_D**	− 0.35	− 0.31	− 0.33	− 0.27	− 0.21	− 0.17	− 0.17	− 0.56 to 0.23
**_cons**	− 5.13***	− 5.13***	− 5.06***	− 5.79***	− 5.85***	− 5.88***	− 5.87***	− 6.8 to −4.9
**Pseudomonas** gut overgrowth								
**TAP**	− 0.65**	− 0.65**	− 0.67***	− 0.68***	− 0.68***	− 0.47*	− 0.57***	− 0.91 to − 0.29
**a_S**	− 1.34***	− 1.33***	− 1.20***	− 1.01***	− 1.00***	− 0.94***	− 0.93***	− 1.46 to − 0.46
**eap**						− 0.21		
**ppap**	0.27	0.27						
**non_D**	−0.33							
**los7**				1.03***	1.03***	0.96***	0.97***	0.53 to 1.45
**trauma50**					0.04	0.03	0.02	− 0.33 to 0.36
**CC**						0.56**	0.56**	0.08 to 1.10
**Acinetobacter** gut overgrowth								
**TAP**	− 0.25	− 0.25	− 0.27	− 0.27	− 0.5	− 0.58	− 0.43	− 1.04 to 0.15
**a_S**	− 1.26*	− 1.27*	− 1.21*	− 1.04*	− 0.85	− 0.8	− 0.82	− 1.83 to 0.19
**eap**						0.25		
**ppap**	0.1	0.1						
**non_D**	0.06							
**los7**				1.15***	1.01***	0.99***	0.98***	0.41 to 1.54
**trauma50**					1.09***	1.04***	1.04***	0.47 to 1.62
**CC**						0.42	0.42	− 0.22 to 1.22
**Error terms**								
**var(e.Ps_GO)**	1.32*	1.32*	1.17**	0.76**	0.76**	0.71**	0.72**	0.36 to 1.47
**var(e.Ac_GO)**	2.66***	2.66***	2.56***	1.92***	1.62***	1.60***	1.60***	1.01 to 2.48
**Model fit**^j^								
**AIC**	3345.94	3344.15	3329.29	3274.57	3261.55	3259.1	3255.53	
***N***	22	20	20	22	24	28	26	
**Groups (*n*)**	334	334	334	334	334	334	334	
**Clusters (*n*)**	213	213	213	213	213	213	213	

*p < 0.05; **p < 0.01; ***p < 0.001

^a^v_ps_n is the count of *Pseudomonas* VAP; v_ac_n is the count of *Acinetobacter* VAP; b_ps_n is the count of *Pseudomonas* bacteremia; and b_ac_n is the count of *Acinetobacter* bacteremia

^b^PPAP is the group-wide use of protocolized parenteral antibiotic prophylaxis; TAP is topical antibiotic prophylaxis; eap is enteral antibiotic prophylaxis

^c^e.Ac_GO is the error term for the *Acinetobacter* gut overgrowth latent variable

^d^e.Ps_GO is the error term for the *Pseudomonas* gut overgrowth latent variable

^e^MVP90 is the use of mechanical ventilation by more than 90% of the group

^f^LOS7 is a mean or median length of ICU stay for the group of 7 days or greater

^g^Trauma ICU arbitrarily defined as an ICU for which > 50% of admissions were for trauma

^h^CC is the concurrency of control groups with an intervention group receiving TAP

^i^Less than 90% of the group receiving prolonged mechanical ventilation.

^j^Model fit; AIC is Akaike’s information criteria. This indicates model fit taking into account the statistical goodness of fit and the number of parameters in the model. Lower values of AIC indicate a better model fit. *N* is the number of parameters in the model

A mean ICU LOS ≥ 7 days was strongly correlated with both *Pseudomonas* gut overgrowth (+ 0.97; 0.53 to 1.45) and *Acinetobacter* gut overgrowth (+ 0.98; 0.41 to 1.54). Exposure to anti-septic interventions was associated with a stronger negative coefficient (− 0.93; − 1.46 to − 0.46) than was exposure to TAP (− 0.57; − 0.91 to − 0.29) towards *Pseudomonas* gut overgrowth but neither exposure was significant towards *Acinetobacter* gut overgrowth. Membership of a control group concurrent to a TAP intervention group was associated with a significant positive coefficient (+ 0.56; 0.08 to 1.10) towards *Pseudomonas* gut overgrowth. PPAP use was a strong positive correlate of *Pseudomonas* bacteremia (+ 0.95; 0.27 to 1.61).

### VAP and bacteremia count data

**Fig. 2:**
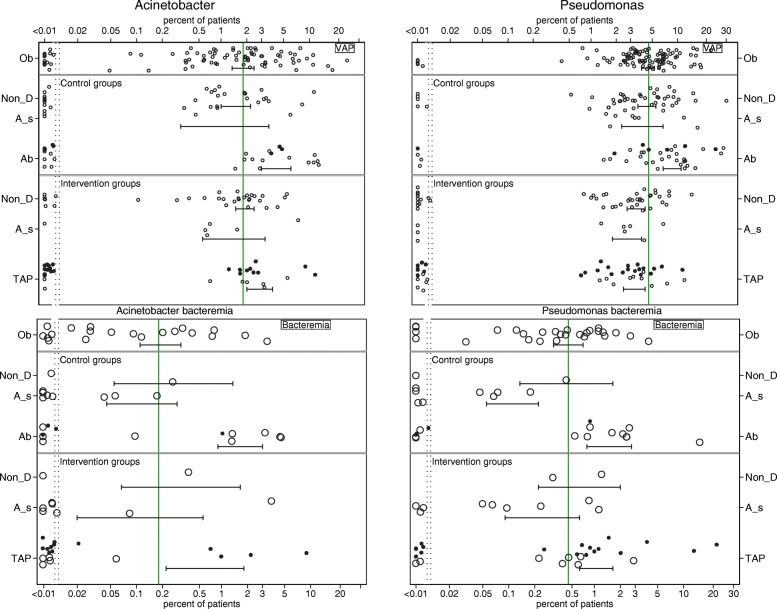
Scatter plots of *Pseudomonas* (right) and *Acinetobacter* (left) VAP (top) and bacteremia (bottom) incidence proportions for the component groups from all studies versus benchmarks derived from observational (Ob) groups. The control and intervention groups are stratified by studies of either non-decontamination (Non-D) methods, anti-septic-based methods (A_s), or antibiotic (Ab, TAP)-based methods. The summary mean and summary 95% confidence intervals are displayed for each category. The derivation of these confidence intervals by random effects methods is displayed in caterpillar plots (Fig S15-S22) in the ESM for the bacteremia data. Note that the *x* axis is a logit scale. Control and intervention groups exposed to PPAP within TAP studies are indicated as solid symbols versus not exposed (open symbols)

The *Pseudomonas* and *Acinetobacter* VAP and bacteremia infection data is presented as percentages (Fig. 2) and as tallied counts (Tables 3 and 4). There were a small number of very large studies with a mean LOS < 7 days and without VAP data. Hence, the tallied counts limited to studies with mean length of stay ≥ 7 days are also shown (Tables 3 and 4).

**Table 3:** VAP count data

	Observational studies	Infection prevention studies		
	No intervention	Non-dec	Anti-septic	TAP ± PPAP
Excluding groups with LOS < 7 days				
*Acinetobacter*				
CC or observational groups	586/37026^a,b^ 1.6% (67)	30/2620^a^ 1.1% (25)	4/780^a^ 0.5% (5)	67/1521^a^ 4.4% (25)
Intervention groups		34/2429^b^ 1.4% (24)	8/786^b^ 1.0% (5)	41/1721^b^ 2.4% (26)
*Pseudomonas*				
CC or observational groups	2217/60131^c, d^ 3.7% (81)	200/4288^c^ 4.7% (38)	27/914^c^ 3.0% (8)	179/2161^c^ 8.3% (34)
Intervention groups		167/4169^d^ 4.0% (37)	24/1027^d^ 2.3% (8)	106/3193^d^ 3.3% (37)

*Non-dec* non-decontamination studies, *TAP* topical antibiotic prophylaxis, *PPAP* protocolized parenteral antibiotic prophylaxis.

^a^The counts of *Acinetobacter* VAP among the three categories of control groups and the category of observation groups among studies after excluding those with length of stay < 7 days differed significantly (*p* < 0.001; Fisher’s exact test)

^b^The counts of *Acinetobacter* VAP among the three categories of intervention groups and the category of observation groups among studies after excluding those with length of stay < 7 days differed significantly (*p* = 0.038; Fisher’s exact test)

^c^The counts of *Pseudomonas* VAP among the three categories of control groups and the category of observation groups among studies after excluding those with length of stay < 7 days differed significantly (*p* < 0.001; Fisher’s exact test)

^d^The counts of *Pseudomonas* VAP among the three categories of intervention groups and the category of observation groups among studies after excluding those with length of stay < 7 days differed marginally (*p* = 0.05; Fisher’s exact test)

**Table 4:** Bacteremia count data

	Observational studies	Infection prevention studies		
	No intervention	Non-dec	Anti-septic	TAP ± PPAP
All groups				
*Acinetobacter*				
CC or observational groups	203/189338 0.11% (20)	1/553 0.18% (2)	17/39162 0.04% (8)	15/1860 0.8% (13)
Intervention groups		1/526 0.19% (2)	7/57009 0.01% (8)	15/13290^a^ 0.11% (18)
*Pseudomonas*				
CC or observational groups	567/192203 0.30% (27)	2/553 0.36% (2)	23/39162 0.06% (8)	63/5280 1.2% (16)
Intervention groups		3/526 0.57% (2)	52/59117 0.09% (9)	139/23543 0.59% (25)
Excluding groups with LOS < 7 days				
*Acinetobacter*				
CC or observational groups	37/12913^b,c^ 0.29% (11)	0/200^b^ 0% (1)	0/308^b^ 0% (3)	14/904^b^ 1.5% (11)
Intervention groups		1/199^c^ 0.5% (1)	1/305^c^ 0.33% (3)	11/1256^c^ 0.88% (14)
*Pseudomonas*				
CC or observational groups	111/14453^d,e^ 0.77% (16)	0/200^d^ 0% (1)	0/308^d^ 0.0% (3)	63/5249^d^ 1.2% (15)
Intervention groups		2/199^e^ 1.0% (1)	17/2413^e^ 0.7% (4)	94/12531^e,f^ 0.75% (22)

*Non-dec* non-decontamination studies, *TAP* topical antibiotic prophylaxis, *PPAP* protocolized parenteral antibiotic prophylaxis.

^a^Among intervention groups of TAP-based prevention studies, the count of *Acinetobacter* bacteremias was 12/6609 (0.18%; 13 studies) versus 3/6681 (0.04%; 4 studies) for those using versus not including PPAP in the intervention (*p* = 0.02; Fisher’s exact test)

^b^The counts of *Acinetobacter* bacteremias among the three categories of control groups and the category of observation groups among studies after excluding those with length of stay < 7 days differed significantly (*p* < 0.001; Fisher’s exact test)

^c^The counts of *Acinetobacter* bacteremias among the three categories of intervention groups and the category of observation groups among studies after excluding those with length of stay < 7 days differed significantly (*p* = 0.012; Fisher’s exact test)

^d^The counts of *Pseudomonas* bacteremias among the three categories of control groups and the category of observation groups among studies after excluding those with length of stay < 7 days differed significantly (*p* = 0.010; Fisher’s exact test)

^e^The counts of *Pseudomonas* bacteremias among the three categories of intervention groups and the category of observation groups among studies after excluding those with length of stay < 7 days were not significantly different (*p* = 0.90; Fisher’s exact test)

^f^Among intervention groups of TAP-based prevention studies excluding those with a LOS less than 7 days, the count of *Pseudomonas* bacteremias was 53/5908 (0.9%; 16 studies) versus 41/6623 (0.62%; 6 studies) for those using versus not including PPAP in the intervention (*p* = 0.07; Fisher’s exact test)

Whether as incidence percentages within individual studies as noted in the caterpillar plots and scatter plots or as counts tallied across all studies, the incidences of infection were generally higher among the control and intervention groups of TAP studies with respect to both *Pseudomonas* and *Acinetobacter* (Fig. 2). There was one exception to this in that the *Pseudomonas* VAP incidences among TAP intervention groups were mostly below the *Pseudomonas* VAP benchmark as was the *Pseudomonas* VAP tallied count among groups with LOS < 7 days excluded (*p* = 0.05; Table 3).

Of note, among the TAP intervention groups, the *Acinetobacter* bacteremia tallied count among groups also exposed to PPAP (12/6609; 0.18%) was higher versus the tallied count among those exposed to TAP alone (3/6681; 0.04%; *p* = 0.02, Fisher’s exact test). Likewise, for *Pseudomonas* bacteremia among the TAP intervention groups after excluding those groups with LOS < 7 days (Table 4), there was a marginally higher tallied count among groups also exposed to PPAP (53/5908; 0.9%) versus the tallied count among those exposed to TAP alone (41/6623; 0.62%; *p* = 0.07, Fisher’s exact test).

## Discussion

Generally accepted risk factors towards the acquisition of Gram-negative bacilli in the ICU include LOS > 7 days, exposure to invasive devices such as MV, and exposure to antibiotics together with acquisition by cross infection within the ICU environment [10]. A GSEM model founded on COGO concepts is used to evaluate these risk factors versus other group-level exposures. This GSEM model enables the component groups of studies of the various infection prevention methods to be considered as a natural experiment with various group-wide exposures among over two hundred ICU populations in the literature. This enables a novel perspective on the COGO concept that would not be possible within any one study examined in isolation nor within several studies examined collectively as within a systematic review [22].

The data used here to confront the COGO model is drawn mostly from studies located in systematic reviews. The extracted data is provided in sufficient detail in the ESM to enable replication of the analysis. In this regard, the summary effect sizes here for each of the three broad categories of TAP, anti-septic, and non-decontamination methods, against both overall VAP and against overall bacteremia, are similar to prior published estimates [1–10]. As has previously been noted, TAP (moreso when in combination with PPAP [23]) appears to have the strongest prevention effect against both overall VAP and against overall bacteremia.

In confronting the COGO model with the *Pseudomonas* and *Acinetobacter* infection data, the COGO model is robust with several factors remaining consistent over the evolution through seven candidate versions of the GSEM. There are several expected observations. Length of stay and admission to a trauma ICU are strong positive factors, and non-decontamination interventions appear not to mediate significant effects on either *Pseudomonas* gut overgrowth or *Acinetobacter* gut overgrowth. TAP exposure is associated with a negative coefficient towards *Pseudomonas* gut overgrowth, albeit weaker than that associated with anti-septic interventions. These negative coefficients in association with TAP and anti-septic exposures towards *Pseudomonas* gut overgrowth reflect the generally lower *Pseudomonas* VAP among the intervention groups of these studies.

On the other hand, the various components of the SOD/SDD regimens, TAP, EAP, and PPAP, have mixed effects within the GSEM models. Neither TAP nor EAP has negative coefficients towards *Acinetobacter* gut overgrowth. This is surprising as in nearly all instances these contain polymyxin and/or an aminoglycoside. Moreover, PPAP is associated with a strong positive correlation with *Pseudomonas* bacteremia.

Finally, patient groups exposed to the full SDD regimen (i.e., all of TAP, EAP, and PPAP) have *Pseudomonas* and *Acinetobacter* bacteremia incidences that are either higher than or else not lower than patient groups receiving TAP alone. This is possibly not paradoxical as antibiotics used for PPAP typically lack activity against *Pseudomonas* and *Acinetobacter.* In this regard, the cumulative days of exposure to antibiotics without activity against *Pseudomonas* have been reported as being a risk factor for acquiring *P. aeruginosa* and *Acinetobacter* in the ICU [24–26]. Moreover, concomitant systemic antibiotic therapy fails to prevent the acquisition of respiratory tract colonization with Gram-negative bacteria [27] and more than triples the risk of subsequent infection among ICU patients receiving an enteral decolonization regimen with gentamicin against KPC-producing *Klebsiella pneumonia* [28] and CRE-producing *Acinetobacter* [29].

The exact relationship between gut colonization, PPAP use, and subsequent bacteremia remains controversial amid conflicting reports that PPAP use may or may not be important for some Gram-negative bacteremias versus others [30–33]. In studying the relative prevention effects of SDD versus SOD each versus standard care in the prevention of Gram-negative bacteremias (i.e., not limited to *Pseudomonas* bacteremia), the majority of bacteremias occur after 4 days in the ICU (the typical duration of PPAP) and indeed the daily risk peaks after day 30 [11, 31]. Moreover, among patients receiving SDD or SOD, *Pseudomonas* accounts for one third of GN bacteremia episodes with most episodes not preceded by enteral colonization.

Defining the separate effects of EAP, TAP, and PPAP on the *Acinetobacter* and *Pseudomonas* bacteremia incidences is difficult as these exposures are confounded with each other among the multiple SDD/SOD regimens under investigation in the different studies. Also, the duration of the application of the regimens and the duration of follow up varied among the studies. In this regard, a non-significant increase in hospital-acquired infections post discharge from the ICU as great as 50% was noted in a small SDD sub-study [34].

In critical care research, SEM is emerging as a method to model the relationships among multiple simultaneously observed variables in order to provide a quantitative test of any theoretical model proposed within the literature [35]. The use of latent variables within the model enables the ability to test the validity of concepts that can only be indirectly quantified through their inferred relationship to observed variables [36]. GSEM allows generalized linear response functions in addition to the linear response functions allowed by SEM.

### Limitations

There are five key limitations to this analysis, the first being that this analysis is a group-level modeling of two latent variables, *Pseudomonas* gut overgrowth and *Acinetobacter* gut overgrowth, within a GSEM founded on the COGO construct. These latent variables and the coefficients derived in the GSEM are indicative and intended for internal reference only. They have no counterpart at the level of any one patient or study and cannot be directly measured. There was no ability nor purpose to adjust for the underlying patient-level risk. There was considerable heterogeneity in the interventions, populations, and study designs among the studies here as the inclusion criteria for the various studies have been intentionally broadly specified. In this regard, a strength of the analysis is that the heterogeneity among the studies here generally resembles that expected among ICU populations to which these interventions might be targeted.

The second limitation is that the analysis is inherently observational. Only a limited number of key group-level factors were entered into the GSEM models. Moreover, the GSEM modeling is deliberately simplistic with exposures entered as only binary variables and without the use of interaction terms. In reality, the relationships between exposures and outcomes will likely be complex and exposure interactions could have great importance.

Thirdly, the analysis is likely underpowered to examine the *Acinetobacter* infection data, being a relatively rare end point. Likewise, the incidences of resistant infections with *Acinetobacter* and *Pseudomonas* are of great interest. However, examination of the incidence of these resistant infections is difficult as these end points are generally uncommon or rare and have been inconsistently reported among these studies here.

Fourthly, only those studies for which *Pseudomonas* and *Acinetobacter* infection data were available were able to be included in this analysis. However, the effect of the interventions on overall VAP and bacteremia incidences among the studies included here (Fig S2-S7) resembles that in the broader literature.

Finally, it should be noted that the various interventions among the studies here targeted a range of sites which may or may not have included the oropharynx and gastrointestinal tract. In this regard, it is surprising that the TAP and EAP interventions, which most directly target the oropharynx and gastrointestinal tract, had weaker effects than did anti-septic interventions, several of which, such as chlorhexidine body washes, target other sites.

Can the paradoxical findings of the GSEM model be reconciled with the apparent superior summary prevention effects of TAP against VAP and bacteremia? TAP exposure and control group concurrency have associations with *Pseudomonas* gut overgrowth that are each similar in size but contrary in direction to each other. In this regard, the incidences of overall VAP, overall bacteremia and also mortality [37] among the concurrent control groups within studies of SOD/SDD are as much as ten percentage points higher than the repsective incidences of these end points among the control groups within studies of equivalent ICU populations. This higher overall VAP incidence can partly be accounted for by incidences of VAP with specific bacteria such as *Acinetobacter* [38], *Pseudomonas* [39], and *Staphylococcus aureus* [40] being each 3 to 5 percentage points higher among CC (but not NCC) control groups and these incidences are generally each up to 2 percentage points higher for the intervention groups of SOD/SDD studies*.*

Likewise, the higher overall bacteremia incidence can partly be accounted for by noting that the incidences of bacteremia with specific bacteria are generally 1 to 4 percentage points higher among CC (but not NCC) control groups. Even among intervention groups, these bacteremia incidences may on average be up to 3 percentage points higher for *Acinetobacter* (Fig. 2), *Pseudomonas* (Fig. 2) [41], *Staphylococcus aureus* [42], *Enterococci* [43], and *coagulase-negative Staphylococci* [44]*.*

In each case, the increased incidence within control groups of CC design studies of topical antibiotics remains apparent in meta-regression models adjusting for other recognized associations. The influence of topical placebo use, concurrent colonization with *Candida*, and other influences may also have influences in this process [45–47].

Hence, reconciling the findings of the GSEM model founded on COGO concepts on the one hand, with the apparent superior summary prevention effects of TAP against VAP and bacteremia, on the other, is possible by noting that the incidences of VAP and bacteremia are generally higher among CC (but not NCC) control groups of studies of TAP. These higher incidences within CC (but not NCC) control groups of studies of TAP remain to be explained.

## Conclusion

TAP-based decontamination regimens appear superior versus other methods at reducing incidences of overall VAP and bacteremia infections among ICU patients. GSEM modeling of *Pseudomonas* gut overgrowth and *Acinetobacter* gut overgrowth as latent variables versus group-level exposures demonstrates complex and paradoxical relationships which would not be apparent in any single study examined in isolation nor within a summary effect of the collective studies as derived by a conventional meta-analysis. Paradoxically, despite the superior apparent infection prevention effect observed among studies of TAP, *Acinetobacter* bacteremia incidences are unusually high among studies of TAP. Moreover, in TAP-exposed groups, the additional exposure to PPAP is associated with higher *Acinetobacter* bacteremia incidences and PPAP is a strongly positive factor towards *Pseudomonas* bacteremia in the GSEM model. In the light of these paradoxical findings, crucially, is TAP safe within the ICU context [13]?

## Supplementary information

**Additional file 1 Table S1.** Pseudomonas and Acinetobacter data: observational studies (Benchmark groups). **Table S2.** Pseudomonas and Acinetobacter data: Groups of non-decontamination studies. **Table S3.** Pseudomonas and Acinetobacter data: Groups of anti-septic studies. **Table S4.** Pseudomonas and Acinetobacter data: Groups of TAP studies. **Table S5.** Development of GSEM model. **Table S6.** VAP count data. **Table S7.** Bacteremia count data. **Figure S1.** Search, screening, triage and decant of studies and groups. **Figure S2.** VAP prevention effect size; non decontamination studies. **Figure S3.** VAP prevention effect size; anti-septic studies. **Figure S4.** VAP prevention effect size; studies of TAP. **Figure S5.** Bacteremia prevention effect size; non-decontamination studies. **Figure S6.** Bacteremia prevention effect size; anti-septic studies. **Figure S7.** Bacteremia prevention effect size; studies of TAP. **Figure S8 - S14.** GSEM models 1–7. **Figure S15.** Pseudomonas bacteremia incidence: observational studies. **Figure S16.** Pseudomonas bacteremia incidence: non-decontamination & anti-septic studies. **Figure S17.** Pseudomonas bacteremia incidence: studies of TAP: control. **Figure S18.** Pseudomonas bacteremia incidence: studies of TAP: intervention. **Figure S19.** Acinetobacter bacteremia incidence: observational studies. **Figure S20.** Acinetobacter bacteremia incidence: non-decontamination & anti-septic studies. **Figure S21.** Acinetobacter bacteremia incidence: studies of TAP: control. **Figure S22.** Acinetobacter bacteremia incidence: studies of TAP: intervention.

## Data Availability

The datasets analyzed during the current study are provided in the online appendix.

## Abbreviations

COGO: Control of gut overgrowth
EAP: Enteral antibiotic prophylaxis
GSEM: Generalized structural equation models
ICU: Intensive care unit
MV: Mechanical ventilation
NCC: Non-concurrent control
CC: Concurrent control
SOD/SDD: Selective oral decontamination/selective digestive decontamination
TAP: Topical antibiotic prophylaxis

## References

[1] Liberati A, D'Amico R, Pifferi S, Torri V, Brazzi L, Parmelli E (2009). Antibiotic prophylaxis to reduce respiratory tract infections and mortality in adults receiving intensive care (review). Cochrane Database Syst Rev.

[2] Pileggi C, Bianco A, Flotta D, Nobile CG, Pavia M (2011). Prevention of ventilator-associated pneumonia, mortality and all intensive care unit acquired infections by topically applied antimicrobial or antiseptic agents: a meta-analysis of randomized controlled trials in intensive care units. Crit Care.

[3] Silvestri L, Van Saene HK, Casarin A, Berlot G, Gullo A (2008). Impact of selective decontamination of the digestive tract on carriage and infection due to Gram-negative and Gram-positive bacteria: a systematic review of randomised controlled trials. Anaesth Intensive Care.

[4] Hurley JC (1995). Prophylaxis with enteral antibiotics in ventilated patients: selective decontamination or selective cross-infection?. Antimicrob Agents Chemother.

[5] Silvestri L, Van Saene HK, Milanese M, Gregori D, Gullo A (2007). Selective decontamination of the digestive tract reduces bacterial bloodstream infection and mortality in critically ill patients. Systematic review of randomized, controlled trials. J Hosp Infect..

[6] Silvestri L, Weir WI, Gregori D, Taylor N, Zandstra DF, van Saene JJ, van Saene HK (2017). Impact of oral chlorhexidine on bloodstream infection in critically ill patients: systematic review and meta-analysis of randomized controlled trials. J Cardiothoracic Vasc Anesthesia.

[7] Labeau SO, Van de Vyver K, Brusselaers N, Vogelaers D, Blot SI (2011). Prevention of ventilator-associated pneumonia with oral antiseptics: a systematic review and meta-analysis. Lancet Infect Dis.

[8] Klompas M, Speck K, Howell MD, Greene LR, Berenholtz SM (2014). Reappraisal of routine oral care with chlorhexidine gluconate for patients receiving mechanical ventilation: systematic review and meta-analysis. JAMA Intern Med.

[9] Alhazzani W, Smith O, Muscedere J, Medd J, Cook D (2013). Toothbrushing for critically ill mechanically ventilated patients: a systematic review and meta-analysis of randomized trials evaluating ventilator-associated pneumonia. Crit Care Med.

[10] Silvestri L, Miguel A, van Saene HK (2012). Selective decontamination of the digestive tract: the mechanism of action is control of gut overgrowth. Intensive Care Med.

[11] Frencken JF, Wittekamp BH, Plantinga NL, Spitoni C, van de Groep K, Cremer OL, Bonten MJ (2017). Associations between enteral colonization with gram-negative bacteria and intensive care unit–acquired infections and colonization of the respiratory tract. Clin Infect Dis.

[12] Hurley JC (2008). Profound effect of study design factors on ventilator-associated pneumonia incidence of prevention studies: benchmarking the literature experience. J Antimicrob Chemother.

[13] Hurley JC (2019). Is selective decontamination (SDD/SOD) safe in the ICU context?. J Antimicrob Chemother.

[14] Agusti C, Pujol M, Argerich MJ, Ayats J, Badia M, Dominguez MA, Corbella X, Ariza J (2002). Short-term effect of the application of selective decontamination of the digestive tract on different body site reservoir ICU patients colonized by multi-resistant *Acinetobacter baumannii*. J Antimicrob Chemother.

[15] Halaby T, al Naiemi N, Kluytmans J, van der Palen J, Vandenbroucke-Grauls CM (2013). Emergence of colistin resistance in *Enterobacteriaceae* after the introduction of selective digestive tract decontamination in an intensive care unit. Antimicrob Agents Chemother.

[16] Lübbert C, Faucheux S, Becker-Rux D (2013). Rapid emergence of secondary resistance to gentamicin and colistin following selective digestive decontamination in patients with KPC-2-producing *Klebsiella pneumoniae*: a single-centre experience. Int J Antimicrob Agents.

[17] Hurley JC (2019). Worldwide variation in Pseudomonas associated ventilator associated pneumonia. A meta-regression. J Crit Care.

[18] Hurley JC (2016). World-wide variation in incidence of *Acinetobacter* associated ventilator associated pneumonia: a meta-regression. BMC Infect Dis.

[19] Goodman L. Snowball sampling. Ann Math Statistics. 1961;32:148–70.

[20] Hurley JC. Forrest plots or caterpillar plots? J Clin Epidemiol. 2020 [in press]. 10.1016/j.jclinepi.2020.01.017 32014534

[21] Stata corporation (2109): Stata structural equation modelling reference manual, in Stata 16 documentation. College Station, TX, USA. https://www.stata.com/bookstore/structural-equation-modeling-reference-manual/. Accessed 6 Jan 2020.

[22] Hurley JC (2020). How the cluster randomized trial ‘works’. Clin Infect Dis.

[23] Oostdijk EAN, Kesecioglu J, Schultz MJ (2014). Notice of retraction and replacement: Oostdijk et al. effects of decontamination of the oropharynx and intestinal tract on antibiotic resistance in ICUs: a randomized clinical trial. JAMA.

[24] Venier AG, Leroyer C, Slekovec C, Talon D, Bertrand X, Parer S, Alfandari S, Guerin JM, Megarbane B, Lawrence C, Clair B (2014). Risk factors for *Pseudomonas aeruginosa* acquisition in intensive care units: a prospective multicentre study. J Hosp Infect.

[25] Hoang S, Georget A, Asselineau J, Venier AG, Leroyer C, Rogues AM, Thiébaut R (2018). Risk factors for colonization and infection by *Pseudomonas aeruginosa* in patients hospitalized in intensive care units in France. PLoS One.

[26] Medina J, Formento C, Pontet J, Curbelo A, Bazet C, Gerez J, Larrañaga E (2007). Prospective study of risk factors for ventilator-associated pneumonia caused by *Acinetobacter* species. J Crit Care.

[27] Jongerden IP, Speelberg B, Satizábal CL, Buiting AG, Leverstein-van Hall MA, Kesecioglu J, Bonten MJ (2015). The role of systemic antibiotics in acquiring respiratory tract colonization with gram-negative bacteria in intensive care patients: a nested cohort study. Crit Care Med.

[28] Tascini C, Sbrana F, Flammini S, Tagliaferri E, Arena F, Leonildi A, Ciullo I, Amadori F, Di Paolo A, Ripoli A, Lewis R (2014). Oral gentamicin gut decontamination for prevention of KPC-producing *Klebsiella pneumoniae* infections: relevance of concomitant systemic antibiotic therapy. Antimicrob Agents Chemother.

[29] Munoz-Price LS, Rosa R, Castro JG, Laowansiri P, Latibeaudiere R, Namias N, Tarima S (2016). Evaluating the impact of antibiotic exposures as time-dependent variables on the acquisition of carbapenem-resistant *Acinetobacter baumannii*. Crit care Med..

[30] Boukadida J, De Montalembert M, Gaillard JL, Gobin J, Grimont F, Girault D, Véron M, Berche P (1991). Outbreak of gut colonization by *Pseudomonas aeruginosa* in immunocompromised children undergoing total digestive decontamination: analysis by pulsed-field electrophoresis. J Clin Microbiol.

[31] Corbella X, Pujol M, Ayats J, Sendra M, Ardanuy C, Dominguez MA, Liñares J, Ariza J, Gudiol F (1996). Relevance of digestive tract colonization in the epidemiology of nosocomial infections due to multiresistant *Acinetobacter baumannii*. Clin Infect Dis.

[32] Oostdijk EA, de Smet AM, Kesecioglu J, Bonten MJ (2011). The role of intestinal colonization with gram-negative bacteria as a source for intensive care unit-acquired bacteremia. Crit Care Med.

[33] Timsit JF, Garrait V, Misset B, Goldstein FW, Renaud B, Carlet J (1993). The digestive tract is a major site for *Acinetobacter baumannii* colonization in intensive care unit patients. J Infect Dis.

[34] de Smet AM, Hopmans TE, Minderhoud AL, Blok HE, Gossink-Franssen A, Bernards AT, Bonten MJ (2009). Decontamination of the digestive tract and oropharynx: hospital acquired infections after discharge from the intensive care unit. Intensive Care Med.

[35] Ricci Z, Romagnoli S, Di Chiara L (2016). Latent AKI is… still AKI: the quantification of the burden of renal dysfunction. Crit Care.

[36] Bojan M, Duarte MC, Ermak N, Lopez-Lopez V, Mogenet A, Froissart M (2016). Structural equation modelling exploration of the key pathophysiological processes involved in cardiac surgery-related acute kidney injury in infants. Crit Care.

[37] Hurley JC. Discrepancies in Control Group Mortality Rates Within Studies Assessing Topical Antibiotic Strategies to Prevent Ventilator-Associated Pneumonia: An Umbrella Review. Critical care explorations. 2020;2(1). 10.1097/CCE.0000000000000076 PMC7063908 32166296

[38] Hurley JC (2018). Paradoxical *Acinetobacter* associated ventilator associated pneumonia incidences within prevention studies using respiratory tract applications of topical polymyxin: benchmarking the evidence base. J Hosp Infect..

[39] Hurley JC (2018). Incidences of *Pseudomonas* aeruginosa-associated ventilator-associated pneumonia within studies of respiratory tract applications of polymyxin: testing the Stoutenbeek concurrency postulates. Antimicrob Agents Chemother.

[40] Hurley J (2018). Unusually high incidences of *Staphylococcus aureus* infection within studies of ventilator associated pneumonia prevention using topical antibiotics: benchmarking the evidence base. Microorganisms..

[41] Hurley JC (2018). Unusually high incidences of *Pseudomonas* bacteremias within topical polymyxin based decolonization studies of mechanically ventilated patients: benchmarking the literature. Open Forum Infect Dis..

[42] Hurley JC (2000). Concordance of endotoxemia with gram-negative bacteremia: a meta-analysis using receiver operating characteristic curves. Arch Pathol & Lab Med.

[43] Hurley JC (2019). Studies of selective digestive decontamination as a natural experiment to evaluate topical antibiotic prophylaxis and cephalosporin use as population-level risk factors for enterococcal bacteraemia among ICU patients. J Antimicrob Chemother.

[44] Hurley JC. Incidence of coagulase-negative staphylococcal bacteremia among ICU patients: decontamination studies as a natural experiment. Eur J Clin Micro Infect Dis. 2019. 10.1007/s10096-019-03763-0. 10.1007/s10096-019-03763-0 PMC7223507 31802335

[45] Hurley JC (2015). ICU-acquired candidemia within selective digestive decontamination studies: a meta-analysis. Intensive Care Med.

[46] Hurley JC (2014). Ventilator-associated pneumonia prevention methods using topical antibiotics: herd protection or herd peril?. Chest..

[47] Hurley JC (2013). The perfidious effect of topical placebo: calibration of Staphylococcus aureus ventilator-associated pneumonia incidence within selective digestive decontamination studies versus the broader evidence base. Antimicrob Agents Chemother.

