# Supplementary material for: Structural equation modeling the “control of gut overgrowth” in the prevention of ICU-acquired Gram-negative infection
Source: Crit Care. 2020 May 4;24:189. doi: 10.1186/s13054-020-02906-6 (PMC7199305; doi:10.1186/s13054-020-02906-6)
Supplement: Supplementary file 1 — Additional file 1 Table S1. Pseudomonas and Acinetobacter data: observational studies (Benchmark groups). Table S2. Pseudomonas and Acinetobacter data: Groups of non-decontamination studies. Table S3. Pseudomonas and Acinetobacter data: Groups of anti-septic studies. Table S4. Pseudomonas and Acinetobacter data: Groups of TAP studies. Table S5. Development of GSEM model. Table S6. VAP count data. Table S7. Bacteremia count data. Figure S1. Search, screening, triage and decant of studies and groups. Figure S2. VAP prevention effect size; non decontamination studies. Figure S3. VAP prevention effect size; anti-septic studies. Figure S4. VAP prevention effect size; studies of TAP. Figure S5. Bacteremia prevention effect size; non-decontamination studies. Figure S6. Bacteremia prevention effect size; anti-septic studies. Figure S7. Bacteremia prevention effect size; studies of TAP. Figure S8 - S14. GSEM models 1–7. Figure S15. Pseudomonas bacteremia incidence: observational studies. Figure S16. Pseudomonas bacteremia incidence: non-decontamination & anti-septic studies. Figure S17. Pseudomonas bacteremia incidence: studies of TAP: control. Figure S18. Pseudomonas bacteremia incidence: studies of TAP: intervention. Figure S19. Acinetobacter bacteremia incidence: observational studies. Figure S20. Acinetobacter bacteremia incidence: non-decontamination & anti-septic studies. Figure S21. Acinetobacter bacteremia incidence: studies of TAP: control. Figure S22. Acinetobacter bacteremia incidence: studies of TAP: intervention. [file 13054_2020_2906_MOESM1_ESM.pdf]

Supplemental file contents:

Table S1: *Pseudomonas* and *Acinetobacter* data: observational studies (Benchmark groups) 2 - 5
Table S2: *Pseudomonas* and *Acinetobacter* data: Groups of non-decontamination studies 6 - 9
Table S3: *Pseudomonas* and *Acinetobacter* data: Groups of anti-septic studies 10 – 11
Table S4: *Pseudomonas* and *Acinetobacter* data: Groups of TAP studies 12 – 15
Table S5: Development of GSEM model 16-17
Table S6: VAP count data 18
Table S7: Bacteremia count data 19-20
Additional details of methods used and additional analyses 21
References 22 – 35
Fig S1. Search, screening, triage and decant of studies and groups 35- 36
Fig S2. VAP prevention effect size; non decontamination studies 37
Fig S3. VAP prevention effect size; anti-septic studies 38
Fig S4. VAP prevention effect size; studies of TAP 39
Fig S5. Bacteremia prevention effect size; non-decontamination studies 40
Fig S6. Bacteremia prevention effect size; anti-septic studies 41
Fig S7. Bacteremia prevention effect size; studies of TAP 42
Fig S8 - S14. GSEM models 1 - 7 43-49
Fig S15. *Pseudomonas* bacteremia incidence: observational studies 50
Fig S16. *Pseudomonas* bacteremia incidence: non-decontamination & anti-septic studies 51
Fig S17. *Pseudomonas* bacteremia incidence: studies of TAP: control 52
Fig S18. *Pseudomonas* bacteremia incidence: studies of TAP: intervention 53
Fig S19. *Acinetobacter* bacteremia incidence: observational studies 54
Fig S20.*Acinetobacter* bacteremia incidence: non-decontamination & anti-septic studies 55
Fig S21. *Acinetobacter* bacteremia incidence: studies of TAP: control 56
Fig S22. *Acinetobacter* bacteremia incidence: studies of TAP: intervention 57

Supplemental file abbreviations: TAP = Topical antibiotic prophylaxis; PPAP = Protocolized parenteral antibiotic prophylaxis; EAP = enteral antibiotic prophylaxis;

Table S1: *Pseudomonas* and *Acinetobacter* data: observational studies (Benchmark groups) [a]

author	Year	Ref	Notes	MV%	LOS	Patients	VAP		Bacteremia	
					d	n	v_ps_n	v_ac_n	b_ps_n	b_ac_n
**A'court**	1993	1	T	100	12	150	17	0	6	
**Alvarez-Lerma**	1996	2		93	9	6494	174	56		
**Antonelli**	1994	3		70	17	124	5	6		
**Apostolopoulou**	2003	4		100	16	175	17	6		
**Baraibar**	1997	5		100	8	707		12		
**Beck-Sague**	1996	6		100	5	145	5	0		
**Bekaert**	2011	7		100	8	4479	155	24		
**Bercault_IHT**	2005	8		100	9	118	9	2		
**Bercault_all**	2005	8		100	10	236	12	5		
**Berrouane_all**	1998	9		83	13	565	40			
**Bochicchio**	2004	10	T	100	13	678	22	10		
**Bonten'94**	1994	11		100	25	64	6	0		
**Boots'06_All**	2006	12		100	13	381	14	14		
**Boots**	2008	13		100	13	412	15	14		
**Bornstain**	2004	14		100	12	747	23	1		
**Braun**	1986	15		100	6	66	0			
**Bregeon**	1997	16		100	10	660	33	10		
**Bronchard**	2004	17	T	100	23	109	0			
**Cade**	1993	18		98	16	98	4	0	1	0
**Cavalcanti**	2006	19		100	5	190	9	1		
**Cenderero**	1999	20		100	7	123	4	2		
**Chaari**	2015	21		100	8	175	20	12		
**Chastre**	1998	22		100	19	243	36	13		
**Chevret**	1993	23		100	5	255	21	5		
**Cook_non-trauma**	2010	24		100	6	2080	7	1		
**Cook_trauma**	2010	24	T	100	7	511	16	8		
**Craven-medical**	1988	25		100	6	277	9	0	1	
**Craven-surgical**	1988	25		100	6	521	17	0	6	
**Daschner**	1988	26		100	6	116	9	1		
**de_Latorre**	1995	27		100	15	80	7	0		
**El-Masri**	2004	28		NS	11	361			1	3
**Ensminger**	2006	29	C	100	7	92	2	1		
**Esteve**	2007	30		80	16	395			8	1
**Esteve**	2007	30		78	17	404			4	6
**Evans**	2010	31		100	8	416	18			
**Ewig**	1999	32		100	10	48	4	0		
**Fabian_all**	1993	33		100	11	278	10	15		
**Fagon'89**	1989	34		100	13	567	16	8		

Table S1 (continued): *Pseudomonas* VAP data: observational studies (Benchmark groups)

author	Year	Ref	Notes	MV%	LOS	Patients	VAP		Bacteremia	
					d	n	v_ps_n	v_ac_n	b_ps_n	b_ac_n
Gacouin	2009	35		100	11	361	21	4		
Garrouste-Orgeas	1997	36		100	11	86	9	11		
Garrouste-Orgeas	2006	37		75	11	3247			19	1
George	1998	38		100	6	223	6	1		
Georges	2000	39		100	18	135	19	8		
Giamarellos-Bourboulis	2009	40	T	100	12	72	5	17	1	2
Giard	2008	41		100	8	7236	168			
Gruson-97-98	2000	42	I	100	12	1029	47	7		
Gruson-95-96	2000	42		100	12	1004	62	20		
Gruson-99-01	2003	43		100	12	823	41	15		
Guérin	1997	44		100	19	260	14	1		
Heyland	1999	45		100	16	1014	38	6		
Holzapfel_93	1993	46		100	10	300	0	1	0	
Hortal	2009	47	C	100	9	231	40	4		
Huang_1SC	2013	48		NS	3	23480			14	7
Huang_1pre	2013	48		NS	3	15816			5	9
Hugonnet	2007	49		100	6	936	31	8		
Hyllienmark	2007	50		100	4	221	0	1		
Ibáñez	2000	51		100	8	30	1	1		
Ibrahim'00	2000	52		69	11	4913			22	8
Ibrahim'00	2000	52		100	5	1882	130	16		
Jaillette	2011	53		100	15	439	59	22		
Jimenez	1989	54		100	6	77	7	6		
Kallel	2005	55		100	14	241	34	31		
Ko	2013	56		100	23	1453			21	11
Kollef' 93	1993	57		100	7	277	4	1		
Kollef '95	1995	58		100	15	300	23	6		
Kollef '97	1997	59		100	8	521	15	2		
Kollef'14_Europe	2014	60		100	11	495	24			
Kollef'14_USA	2014	60		100	11	502	17			
Koss– N	2001	61		100	10	87	4	2		
Koss– P	2001	61	I	100	8	66	9	2		
Kunac	2014	62		100	5	716	23	13	2	1
Laggner	1989	63		100	11	32	0	0		
Lambert	2011	64		NS	5	119699			389	143
Laupland	2002	65		NS	5	1017			2	0
Laupland	2004	66		84	5	4473			11	4

Table S1 (continued): ***Pseudomonas*** **VAP data: observational studies (Benchmark groups)**

author	Year	Ref	Notes	MV%	LOS	Patients	VAP		Bacteremia	
					d	n	v_ps_n	v_ac_n	b_ps_n	b_ac_n
**Lepelletier**	2010	67	T	100	13	161	10			
**Luyt**	2005	68		100	35	290	11	3		
**Magnason**	2008	69		100	8	280	5	0	0	0
**Magret_trauma**	2010	70	T	100	5	354	17	30		
**Magret_non-trauma**	2010	70		100	5	2082	64	42		
**Mahul**	1992	71		100	17	145	8	3		
**Makris**	2011	72	I	100	22	152	7			
**Markowicz**	2000	73		100	12	744	58	13		
**Moine**	2002	74		80	14	764	27	1		
**Montecalvo_G**	1992	75		100	10	19			0	
**Myny**	2005	76		100	6	385	28	13		
**Nguile-Makao**	2010	77		100	7	2873	130			
**Nielsen**	1992	78		100	5	242	3	0		
**Nseir**	2005	79		100	24	1241	32	20		
**Osmon**	2003	80		72	8	893			9	
**Papazian**	1996	81		100	16	586	26	5		
**Potgieter**	1987	82		78	9	250	26	32		
**Rello'91**	1991	83		100	8	264	14	2		
**Rello'92**	1992	84		67	9	161	4			
**Rello'94**	1994	85		72	7				16	5
**Rello'94**	1994	86		100	13	568	18			
**Rello'96**	1996	87		100	8	83	4			
**Rello'02**	2002	88		100	8	9080	119			
**Rello'03**	2003	89		100	7	99	8	2		
**Reusser**	1989	90		100	7	40	2	1	0	0
**Rincón-Ferrari**	2004	91		100	10	310	6	27		
**Rodriguez**	1991	92	T	100	14	294	31	11		
**Ruiz-Santana**	1987	93		100	7	1005	56	0		
**Salata**	1987	94		100	14	51	7	0		
**Shahin**	2013	95		100	6	267	4			
**Sligl**	2006	96		NS	6	6544			10	1
**Sofianou**	2000	97		100	36	198	19	35		
**Stéphan**	2006	98	T	100	16	175	14	8		
**Tejada-Artigas**	2001	99		100	12	103	5	10		
**Thompson**	2008	100		NS	6	4270			13	1
**Timsit**	1996	101		100	19	387	11	8		
**Torres**	1990	102		100	4	322	5	9		

Table S1 (continued): ***Pseudomonas*** **VAP data: observational studies (Benchmark groups)**

author	Year	Ref	Notes	MV%	LOS	Patients	**VAP**		**Bacteremia**	
					d	n	v_ps_n	v_ac_n	b_ps_n	b_ac_n
**Trouillet**	1998	103		100	17	498	39	22		
**Urli**	2002	104		95	21	178	27		3	
**Valles**	2007	105		100	22	60	15	0		
**Vanhems**	2011	106		100	6	3387	24			
**Verhamme**	2007	107		84	8	4000	54	3		
**Violan**	1998	108		100	16	314	25	1		
**Warren**	2001	109		28	4	3163			3	0
**Woske**	2001	110		100	15	103	8	1		
**Zahar**	2009	111		100	9	1233	62			

Table S1 footnotes

T – Data originating from a study for which the majority of ICU admission were for trauma

I – Infection control intervention to entire ICU

NS – Not stated; LOS is mean or median length of ICU stay

v_ps_n is the count of *Pseudomonas* VAP and v_ac_n is the count of *Acinetobacter* VAP

b_ps_n is the count of *Pseudomonas* bacteremia and b_ac_n is the count of *Acinetobacter* bacteremia

Several (n = 43) of these studies were cited in the following source systematic reviews.

- Melsen WG, Rovers MM, Bonten MJM: Ventilator-associated pneumonia and mortality: A systematic review of observational studies. *Crit Care Med* 2009, 37:2709–2718.
- Safdar N, Dezfulian C, Collard HR, Saint S: Clinical and economic consequences of ventilator-associated pneumonia: a systematic review. *Crit Care Med* 2005, 33:2184–93.
- Agrafiotis M, Siempos II, Ntaidou TK, Falagas ME. Attributable mortality of ventilator-associated pneumonia: a meta-analysis. *Intern J Tub Lung Dis.* 2011;15(9):1154-1163.

Table S2: ***Pseudomonas*** **and** ***Acinetobacter*** **data: Groups of non decontamination studies** [a]

author	Year	Ref	Notes	MV%	LOS	Patients	VAP		Bacteremia	
					d	n	v_ps_n	v_ac_n	b_ps_n	b_ac_n
**Acosta-escribano**	2010	112	T	100	18	54	4	0		
**Acosta-escribano**	2010	112	T	100	16	50	3	0		
**Bonten '95**	1995	113		100	17	67	11	0		
**Bonten '95**	1995	113		100	19	74	7	1		
**Combes**	2000	114	T	100	16	50	0			
**Combes**	2000	114	T	100	20	54	0			
**Cook**	1998	115		100	9	596	20			
**Cook**	1998	115		100	9	604	21			
**Dreyfuss**	1991	116		100	13	35	3	3		
**Dreyfuss**	1991	116		100	10	28	1	2		
**Daumal**	1999	117		100	7	174	11			
**Daumal**	1999	117		100	7	187	12			
**Drakulovic**	1999	118		100	10	47	3	1		
**Drakulovic**	1999	118		100	9	39	1	0		
**Dreyfuss**	1995	119		100	13	70	1	2		
**Dreyfuss**	1995	119		100	10	61	0	3		
**Driks**	1987	120		100	14	69	5			
**Driks**	1987	120		100	11	61	1			
**Forestier**	2008	121		100	13	106	8			
**Forestier**	2008	121		100	13	102	3			
**Heyland**	1999	122		100	12	46	0			
**Heyland**	1999	122		100	13	49	0			
**Holzapfel_C_99**	1999	123		100	15	200	6	3	0	0
**Holzapfel_I_99**	1999	123		100	17	199	10	4	2	1
**Kirschenbaum**	2002	124		100	12	20	5			
**Kirschenbaum**	2002	124		100	12	17	1			
**Kirton**	1997	125		100	16	140	6			
**Kirton**	1997	125		100	20	140	6			
**Knight**	2009	126		100	7	129	1	1		
**Knight**	2009	126		100	6	130	0	3		
**Kollef '97_pre**	1997	127		90	3	353	7	2	2	1
**Kollef '97_post**	1997	127		90	2	327	3	1	1	0
**Kollef**	2008	128		100	4	743	11	5		
**Kollef_silverETT**	2008	128		100	4	766	8	1		
**Kortbeek**	1999	129	T	100	7	43	0	1		
**Kortbeek**	1999	129	T	100	10	37	0	1		

Table S2 (continued): ***Pseudomonas*** **and** ***Acinetobacter*** **data: Groups of non decontamination studies** [a]

author	Year	Ref	Notes	MV%	LOS	Patients	VAP		Bacteremia	
					d	n	v_ps_n	v_ac_n	b_ps_n	b_ac_n
**Lacherade '05**	2005	130		100	25	184	14	0		
**Lacherade '05**	2005	130		100	21	185	9	2		
**Lacherade '10**	2010	131		100	11	164	16	2		
**Lacherade '10**	2010	131		100	11	169	9	2		
**Laueny**	2014	132		100	10	91	0	0		
**Laueny**	2014	132		100	15	98	0	1		
**Lorente '03**	2003	133		100	18	116	10	1		
**Lorente '03**	2003	133		100	16	114	9	0		
**Lorente '04**	2004	134		100	16	143	8	1		
**Lorente '04**	2004	134		100	20	161	9	3		
**Lorente'05**	2006	135		100	13	233	12	2		
**Lorente'05**	2006	135		100	13	210	12	1		
**Lorente'06a**	2005	136		100	10	221	7	1		
**Lorente'06a**	2005	136		100	10	236	9	1		
**Lorente'06b**	2006	137		100	21	51	5			
**Lorente'06b**	2006	137		100	20	53	2			
**Lorente'07**	2007	138		100	16	140	4	1		
**Lorente'07**	2007	138		100	14	140	4	3		
**Lorente'14**	2014	139		100	16	150	6	1		
**Lorente'14**	2014	139		100	15	134	3	0		
**Manzano**	2008	140		100	12	63	0	4		
**Manzano**	2008	140		100	9	64	0	2		
**Martin**	1993	141		100	4	66	2	0		
**Martin**	1993	141		100	4	65	0	1		
**Morrow**	2010	142		100	15	73	6	2		
**Morrow**	2010	142		100	15	73	0	3		
**Nseir**	2011	143		100	10	61	2	2		
**Nseir**	2011	143		100	12	61	0	1		
**Pickworth**	1993	144	T	100	7	44	1			
**Pneumatikos**	2006	145		100	15	40	1	1		
**Pneumatikos**	2006	145		100	16	39	0	1		
**Prod'hom_A**	1994	146		100	6	81	4	0		
**Prod'hom_R**	1994	146		100	6	80	1	1		
**Prod'hom_S**	1994	146		100	5	83	1	0		
**Reigneir**	2013	147		100	7	222	9			
**Reigneir**	2013	147		100	7	227	12			
**Ryan_C**	1993	148		100	5	56	2	1		
**Ryan_S**	1993	148		100	6	58	1	0		

Table S2 (continued): *Pseudomonas* and *Acinetobacter* data: Groups of non decontamination studies [a]

author	Year	Ref	Notes	MV%	LOS	Patients	VAP		Bacteremia	
					d	n	v_ps_n	v_ac_n	b_ps_n	b_ac_n
**Smulders**	2002	149		100	14	75	3			
**Smulders**	2002	149		100	12	75	1			
**Staudinger**	2010	150		100	14	75	5			
**Staudinger**	2010	150		100	8	75	3			
**Thomachot**	1998	151		100	12	66	3	1		
**Thomachot**	1998	151		100	12	70	2	1		
**Thomachot**	1999	152		100	11	77	1	0		
**Thomachot**	1999	152		100	12	63	2	0		
**Thomachot**	2002	153		100	10	84	2	0		
**Thomachot**	2002	153		100	9	71	0	1		
**Valencia**	2007	154		100	13	69	1	0		
**Valencia**	2007	154		100	13	73	3	1		
**Valles**	1995	155		100	6	77	12			
**Valles**	1995	155		100	12	76	12			
**Walaszek**	2017	156		100	5	804	5	39		
**Walaszek**	2017	156		100	5	1003	10	22		

Table S2 footnotes

T – Data originating from a study for which the majority of ICU admission were for trauma

NS – Not stated; LOS is mean or median length of ICU stay

v_ps_n is the count of *Pseudomonas* VAP and v_ac_n is the count of *Acinetobacter* VAP

b_ps_n is the count of *Pseudomonas* bacteremia and b_ac_n is the count of *Acinetobacter* bacteremia

Several (n = 47) of these studies were cited in the following source systematic reviews.

- Messori A, Trippoli S, Vaiani M, Gorini M, Corrado A: Bleeding and pneumonia in intensive care patients given ranitidine and sucralfate for prevention of stress ulcer: meta-analysis of randomised controlled trials. *BMJ* 2000, 321:1103–1106.
- Huang J, Cao Y, Liao C, Wu L, Gao F: Effect of histamine-2-receptor antagonists versus sucralfate on stress ulcer prophylaxis in mechanically ventilated patients: a meta-analysis of 10 randomized controlled trials. *Crit Care* 2010, 14:R194.
- Alhazzani W, Almasoud A, Jaeschke R, Lo BW, Sindi A, Altayyar S, Fox-Robichaud A: Small bowel feeding and risk of pneumonia in adult critically ill patients: a systematic review and meta-analysis of randomized trials. *Crit Care* 2013, 17:R127.
- Melsen WG, Rovers MM, Bonten MJM: Ventilator-associated pneumonia and mortality: A systematic review of observational studies. *Crit Care Med* 2009, 37:2709–2718.
- Safdar N, Dezfulian C, Collard HR, Saint S: Clinical and economic consequences of ventilator-associated pneumonia: a systematic review. *Crit Care Med* 2005, 33:2184–93.
- Han J, Liu Y. Effect of ventilator circuit changes on ventilator-associated pneumonia: a systematic review and meta-analysis. *Respiratory care*, 2010; 55: 467-474.

- Subirana M, Solà I, Benito S: Closed tracheal suction systems versus open tracheal suction systems for mechanically ventilated adult patients. *Cochrane Database Syst Rev* 2007, 4: CD004581;
- Siempos II, Vardakas KZ, Kopterides P, Falagas ME. Impact of passive humidification on clinical outcomes of mechanically ventilated patients: A meta-analysis of randomized controlled trials. *Crit Care Med* 2007; 35: 2843-51;
- Muscedere J, Rewa O, McKechnie K, Jiang X, Laporta D, Heyland DK. Subglottic secretion drainage for the prevention of ventilator-associated pneumonia: a systematic review and meta-analysis. *Crit Care Med* 2011; 39:1985–1991.
- Delaney A, Gray H, Laupland KB, Zuege DJ. Kinetic bed therapy to prevent nosocomial pneumonia in mechanically ventilated patients: a systematic review and meta-analysis. *Crit Care* 2006; 10:R70;
- Sud S, Friedrich JO, Taccone P, Polli F, Adhikari NK, Latini R, Gattinoni L. Prone ventilation reduces mortality in patients with acute respiratory failure and severe hypoxemia: systematic review and meta-analysis. *Inten Care Med* 2010; 36(4); 585-599.
- Siempos II, Vardakas KZ, Falagas ME. Closed tracheal suction systems for prevention of ventilator-associated pneumonia. *Brit J Anaesthesia*, 2008; 100(3): 299-306.

Table S3: *Pseudomonas* and *Acinetobacter* data: Groups of anti-septic studies [a]

author	Year	Ref	Notes	MV%	LOS	Patients	VAP		Bacteremia	
					d	n	v_ps_n	v_ac_n	b_ps_n	b_ac_n
Cabov	2010	157		57	6	30	4	0	0	0
Cabov Chlx	2010	157		77	6	30	0	0	0	0
Climo_NC	2013	158		NS	6	1398			2	2
Climo	2013	158		NS	6	1410			4	1
Fourrier'00	2000	159		100	24	30	4	2	0	0
Fourrier'00 Chlx	2000	159		100	18	30	1	2	0	1
Fourrier'05	2005	160		100	13	114	5	0	0	0
Fourrier'05 Chlx	2005	160		100	14	114	6	1	1	0
Huang_2pre	2013	48		NS	3	15218			8	5
Huang_3pre	2013	48		NS	3	17356			11	10
Huang_3UD	2013	48		NS	3	26024			14	3
Huang_2TD	2013	48		NS	3	24752			13	2
Koeman	2006	161		100	13	130	4			
Koeman-Ch	2006	161		100	14	127	0			
Koeman ChC	2006	161		100	13	128	2			
Kollef	2006	162		100	14	347	9	2		
Kollef Iseganan	2006	162		100	14	362	8	2		
Lorente	2012	163		100	9	219	5	0		
Lorente Chlx	2012	163		100	10	217	5	2		
Noto_pre	2015	164		NS	3	4852			2	0
Noto_Chlx BW	2015	164		NS	3	4488			4	0
Seguin – CC	2006	165		100	19	31	0			
Seguin – SC	2006	165	T	100	14	31	1			
Seguin-PVI	2006	165	T	100	15	36	0			
Seguin	2014	166	T	100						
Seguin-PVI	2014	166	T	100						
Swan	2016	167		100	7	70	1	0	0	0
Swan Chlx BW	2016	167		100	7	63	1	1	0	0
Wittekamp	2018	168		100	10	2251			21	
Wittekamp Chlx	2018	168		100	10	2108			16	

Table S3: Footnotes

T – Data originating from a study for which the majority of ICU admission were for trauma

NS – Not stated; LOS is mean or median length of ICU stay

v_ps_n is the count of *Pseudomonas* VAP and v_ac_n is the count of *Acinetobacter* VAP

b_ps_n is the count of *Pseudomonas* bacteremia and b_ac_n is the count of *Acinetobacter* bacteremia

Several (n = 5) of these studies were cited in the following source meta-analyses.

- Chan EY, Ruest A, Meade MO, Cook DJ: Oral decontamination for prevention of pneumonia in mechanically ventilated adults: systematic review and meta-analysis. *BMJ* 2007, 334:889–900.
- Labeau SO, Van de Vyver K, Brusselaers N, Vogelaers D, Blot SI: Prevention of ventilator-associated pneumonia with oral antiseptics: a systematic review and meta-analysis. *Lancet Infect Dis* 2011, 11:845-854.
- Pileggi C, Bianco A, Flotta D, *et al*. Prevention of ventilator-associated pneumonia, mortality and all intensive care unit acquired infections by topically applied antimicrobial or antiseptic agents: a meta-analysis of randomized controlled trials in intensive care units. *Crit Care.* 2011; **15**: R155.
- Price R, MacLennan G, Glen J. Selective digestive or oropharyngeal decontamination and topical oropharyngeal chlorhexidine for prevention of death in general intensive care: systematic review and network meta-analysis. *BMJ*. 2014; **348**: g2197.
- Klompas M, Speck K, Howell MD, *et al*. Reappraisal of routine oral care with chlorhexidine gluconate for patients receiving mechanical ventilation: systematic review and meta-analysis. *JAMA Intern Med.* 2014; **174**: 751-61.

Intervention regimens abbreviations

Chlx = chlorhexidine; Chlx BW = chlorhexidine body wash; ChC = chlorhexidine and colisitn; TD = targetted decolonization; UD = universal decolonization; PVI = povidone iodine; CC = concurrent control; SC = saline control; iseganan, is a synthetic variant of a porcine protegrin, which is a natural antibiotic peptide released by neutrophils in response to invasion by microbes [Kollef 2006, 162].

Table S4: *Pseudomonas* and *Acinetobacter* data: Groups of studies of topical antibiotics [a]

author	PPAP	Year	Ref	Notes	MV%	LOS	Patients	VAP		Bacteremia	
						d	n	v_ps_n	v_ac_n	b_ps_n	b_ac_n
groups with NCC											
**Wittekamp PTNy**		2018	168		100	10	2224			15	
**Wittekamp PTNy**		2018	168		100	11	2082			9	
**Bergmans NC**		2001	169		100	12	61	5			
**Bergmans_PGV**		2001	169		100	15	78	8			
**Bergmans**		2001	169		100	13	87	3			
**Bonten NC**		1994	170		91	16	54	4			
**Bonten CC**		1994	170		86	9	21	0			
**Bonten_PTA**		1994	170		100	13	22	0			
**Camus**		2014	171		28	4	925			0	1
**Camus_PTA**		2014	171		28	4	1022			3	0
**de Smet**		2009	172		88	9	1990			36*	
**de Smet_PTA**		2009	172		94	9	1904			17*	
**de Smet_PTA-Ctx**	+	2009	172		93	9	2045			16*	
**Godard**		1990	173		80	13	84	5			
**Godard_PT**		1990	173		81	11	97	0			
**Hartenauer_Ctx**	+	1991	174		100	12	40	7	1	1	
**Hartenauer_Ctx**	+	1991	174		100	13	61	13	3	0	
**Hartenauer_PTA-Ctx**	+	1991	174		100	17	50	0	0	0	
**Hartenauer_PTA-Ctx**	+	1991	174		100	13	49	0	0	1	1
**Oostdijk**		2011	175		88	9	1945			25*	
**Oostdijk_PTA**		2011	175		94	9	2166			16*	
**Oostdijk_PTA-Ctx**	+	2011	175		93	9	2667			14*	
**Stoutenbeek'84 NC**		1984	176		100	14	59	5	1	1	0
**Stoutenbeek_PTA-Ctx**	+	1984	176		100	11	63	3	1	0	0
**Stoutenbeek'87 NC**		1987	177		100	14	59	5	1		0
**Stoutenbeek_PTA**	+	1987	177		100	18	42	1	2	1	2
**Winter NC**		1992	178		92	7	84	2	0		

Table S4(continued): ***Pseudomonas*** **and** ***Acinetobacter*** **data: Groups of studies of topical antibiotics** [a]

author	PPAP	Year	Ref	Notes	MV%	LOS	Patients	**VAP**		**Bacteremia**	
						d	n	v_ps_n	v_ac_n	b_ps_n	b_ac_n
**groups with CC**											
**Winter CC**		1992	178		92	8	92	8	2		
**Winter_PTA-Cz**	+	1992	178		92	6	91	3	0		
**Abele-Horn**		1997	179	T	100	22	30	3	0		
**Abele-Horn_PTA-Ctx**	+	1997	179	T	100	18	58	2	0		
**Acquarolo**		2005	180		100	13	19	2	0	0	
**Acquarolo_AmpSul**	+	2005	180		100	13	19	2	0	0	
**Aerdts**		1991	181		100	28	39	10	3		
**Aerdts_PNoA-Ctx**	+	1991	181		100	23	17	0	0		
**Bion_-Ctx**	+	1991	182		50	2	31	3	0	0	0
**Bion_PTA-Ctx**	+	1991	182		50	2	21	0	0	2	0
**Blair**		1991	183		93	8	130	9		3	
**Blair_PTA-Ctx**	+	1991	183		93	8	126	1		1	
**Cockerill**		1992	184		85	12	75	4			
**Cockerill_PGNy-Ctx**	+	1992	184		85	10	75	1			
**de la Cal**		2005	185	T	80	34	54	7	5	7	3
**de la Cal_PTA-Ctx**	+	2005	185	T	74	31	53	2	6	9	6
**Ferrer_Ctx**	+	1994	186		100	14	41	4	0	0	0
**Ferrer_PTA-Ctx**	+	1994	186		100	15	39	1	0	1	0
**Georges**		1994	187		100	16	33		0		
**Georges_PNeA**		1994	187		100	16	31		1		
**Hammond_Ctx**	+	1993	188		100	21	20		1		
**Hammond_PTA-Ctx**	+	1993	188		100	30	13		0		
**Hammond_Ctx**	+	1994	189		100	6	33	0	0		
**Hammond_PTA-Ctx**	+	1994	189		100	7	39	0	1		
**Jacobs**		1992	190		100	10	43	0	0	0	0
**Jacobs_PTA-Ctx**	+	1992	190		100	9	36	0	0	0	1
**Karvouniaris**		2015	191		100	13	84		11		
**Karvouniaris_P**		2015	191		100	16	84		2		
**Korinek**		1993	192		100	27	60	3	1		
**Korinek_PTA-V**		1993	192		100	25	63	0	1		
**Laggner**		1994	193		100	20	34	1		1	0
**Laggner_GA**		1994	193		100	16	33	0		0	0
**Palomar**		1997	194		100	6	42	6	0		
**Palomar Ctx**	+	1997	194		100	8	46	1	2		
**Palomar_PTA-Ctx**	+	1997	194		100	11	41	1	0		
**Pneumatikos**		2002	195	T	100	23	30	1	1		
**Pneumatikos_PTA**		2002	195	T	100	16	31	0	1		

Table S4(continued): *Pseudomonas* and *Acinetobacter* data: Groups of studies of topical antibiotics

author	PPAP	Year	Ref	Notes	MV%	LOS	Patients	VAP		Bacteremia	
						d	n	v_ps_n	v_ac_n	b_ps_n	b_ac_n
**groups with CC**											
**Quinio**		1995	196	T	100	16	72	12		0	0
**Quinio_PGA**		1995	196	T	100	16	76	5		0	0
**Rocha**		1992	197	T	100	18	54	8	4	1	3
**Rocha__PTA-Ctx**	+	1992	197	T	100	19	47	1	1	0	0
**Rodríguez-Roldán**		1990	198		100	10	15	5	2		
**Rodríguez-Roldán__PTNeA**		1990	198		100	10	13	0	0		
**Rolando_CtxFlux**		1996	199		73	7	61	2	0	0	0
**Rolando_PTA_CtxFlux**	+	1996	199		73	7	47	1	0	0	0
**Rolando '93**	+	1993	200		75	8	31	2	0		
**Rolando__PTAM-Cfu**	+	1993	200		75	8	28	1	0		
**Sirvent**		1997	201		100	16	50	1	2		
**Sirvent_Cef**	+	1997	201		100	13	50	3	1		
**Smith_CtxAmp**	+	1993	202		100	8	18	1			
**Smith__PTA_CtxAmp**	+	1993	202		100	7	18	0			
**Stoutenbeek**		2007	203	T	100	12	200	28	23	2	3
**Stoutenbeek_PTA-Ctx**	+	2007	203	T	100	13	201	11	15	3	0
**Unertl**		1987	204		100	23	20	2			
**Unertl_PGA**		1987	204		100	18	19	0			
**Verwaest**		1997	205		100	19	185	7	4	1	2
**Verwaest OA_O**	+	1997	205		100	17	193	2	4	2	2
**Verwaest_PTA-Ctx**	+	1997	205		100	22	200	10	4	6	2
**Wiener**		1995	206		100	11	31	0	0		
**Wiener_PGNy**		1995	206		100	11	30	2	1		
**Studies without control groups**											
**Frencken_PTA-Ctx**	+	2014	207		100	7	1874			15	
**Garbino_PNeV**		2002	208		100	9	204	4			
**Leone_PTA-Cef**	+	2002	209		100	6	324	3	5		
**Nardi_ PTA**		2001	210	T	100	12	104	4	1		
**Nardi_PTAM**		2001	210	T	100	11	119	3	0		
**Oostdijk_PTA**		2014	211		52	6	5508			23	3
**Oostdijk_PTA-Ctx**	+	2014	211		51	6	5483			20	1
**Rouby_P**		1994	212		100	12	347	12			
**Rouby E**		1994	212		100	18	251	26			
**Silvestri_PTA-Ctx**	+	1999	213		100	9	117	2		0	
**Steffen_PTNy-Ctx**	+	1994	214		100	14	127			1	0

Table S4: Footnotes

T – Data originating from a study for which the majority of ICU admission were for trauma

NS – Not stated; LOS is mean or median length of ICU stay; PPAP is use of protocolized parenteral antibiotic prophylaxis

v_ps_n is the count of *Pseudomonas* VAP and v_ac_n is the count of *Acinetobacter* VAP

b_ps_n is the count of *Pseudomonas* bacteremia and b_ac_n is the count of *Acinetobacter* bacteremia

* De Smet 2009 [172] The *Pseudomonas* and *Acinetobacter* counts in this study are available only as a combined category count for gram negative non-fermentative GNB and are provided here for reference but are not used in the analysis. The Pseudomonas counts in the study of Oostdijk [175] includes counts from the study by De Smet 2009 [172]. The Pseudomonas counts in the study of Oostdijk [175] are taken as given in Table 2 of Oostdijk [175] even though this Table appears to be mislabelled in comparison to the text and table 1 of Oostdijk [175] and in relation to the data in De Smet 2009 [172].

The control group in one study by Stoutenbeek [176] appears also as the control group in another study by this author [177] and is used only once in the analysis here.

Several (n = 24) of these studies were cited in the following source systematic reviews.

- Liberati A, D'Amico R, Pifferi S, Torri V, Brazzi L, Parmelli E: Antibiotic prophylaxis to reduce respiratory tract infections and mortality in adults receiving intensive care. *Cochrane Database Syst Rev* 2009, 4.
- Pileggi C, Bianco A, Flotta D, Nobile CG, Pavia M. Prevention of ventilator-associated pneumonia, mortality and all intensive care unit acquired infections by topically applied antimicrobial or antiseptic agents: a meta-analysis of randomized controlled trials in intensive care units. *Crit Care* 2011; 15:R155.
- Silvestri L, Van Saene HK, Milanese M, Gregori D. Impact of selective decontamination of the digestive tract on fungal carriage and infection: systematic review of randomized controlled trials. *Intensive Care Med* 2005, 31:898-910.
- Chan EY, Ruest A, Meade MO, Cook DJ. Oral decontamination for prevention of pneumonia in mechanically ventilated adults: systematic review and meta-analysis. *BMJ.* 2007; 334:889–900.

TAP intervention regimens abbrevations; PTA (=P, topical polymyxin; T, topical tobramycin; A, topical amphotericin); PTA-Ctx (=P, topical polymyxin; T, topical tobramycin; A, topical amphotericin; Ctx, parenteral cephalosporin); P (P = polymyxin either aerosolized or topical); PNeV (P = polymyxin; Ne = Neomycin; V = Vancomycin); PGA-Ctx (=P, topical polymyxin; G, topical gentamicin; A, topical amphotericin; Ctx, parenteral cephalosporin); PTAM (=P, topical polymyxin; T, topical tobramycin; A, topical amphotericin; topical mupirocin); E (=E, topical erythromycin); PNoA-Ctx (=P, topical polymyxin; No, topical norfloxacin; A, topical amphotericin; Ctx, parenteral cephalsporin); PGV (=P, topical polymyxin; G, topical gentamicin; V, topical vancomycin); PGNy-Ctx (=P, topical polymyxin; G, topical gentamicin; Ny, topical nystatin; Ctx, parenteral cephalsporin); P-Ctx (=P, topical polymyxin; Ctx, parenteral cephalosporin); PTAV (=P, topical polymyxin; T, topical tobramycin; A, topical amphotericin; V, topical vancomycin); PGA (=P, topical polymyxin; G, topical gentamicin; A, topical amphotericin); PTNeA (=P, topical polymyxin; T, topical tobramycin; Ne, topical Neomycin; A, topical amphotericin); PGNy (=P, topical polymyxin; G, topical gentamicin; Ny, topical nystatin); PTA-Cz (=P, topical polymyxin; T, topical tobramycin; A, topical amphotericin; Cz, parenteral Ceftazidime).

Table S5: Development of GSEM model [a]

	Model 1	Model 2	Model 3	Model 4	Model 5	Model 7	Model 6	
	Fig S8	Fig S9	Fig S10	Fig S11	Fig S12	Fig S12	Fig S13	95%CI
Factor [b-j]								
b_Ps_n								
Pseudomonas	1	1	1	1	1	1	1	(constrained)
ppap			1.11**	0.97**	0.97**	1.00**	0.95**	0.27 to 1.61
_cons	-5.18***	-5.19***	-5.38***	-6.00***	-6.00***	-6.05***	-6.05***	-6.6 to -5.4
b_Ac_n								
Acinetobacter	1	1	1	1	1	1	1	(constrained)
ppap			0.6	0.46	0.48	0.44	0.47	-0.51 to 4639
_cons	-6.74***	-6.74***	-6.83***	-7.38***	-7.44***	-7.47***	-7.47***	-8.0 to -7.0
v_Ps_n								
Pseudomonas	0.67***	0.67***	0.71***	0.80***	0.80***	0.81***	0.81***	0.51 to 1.09
mvp90	0.55*	0.54*	0.49*	0.43	0.43	0.48*	0.49*	0.03 to 0.92
non_D	-0.37*	-0.58***	-0.61***	-0.60***	-0.60***	-0.54***	-0.54***	-0.79 to -0.31
_cons	-3.63***	-3.63***	-3.56***	-4.17***	-4.17***	-4.24***	-4.25***	-4.7 to -3.7
v_Ac_n								
Acinetobacter	0.73***	0.73***	0.74***	0.83***	0.83***	0.83***	0.83***	+0.66 to 1.01
mvp90	0.79*	0.79*	0.73	0.71	0.69	0.71	0.7	-0.12 to 1.55
non_D	-0.35	-0.31	-0.33	-0.27	-0.21	-0.17	-0.17	-0.56 to 0.23
_cons	-5.13***	-5.13***	-5.06***	-5.79***	-5.85***	-5.88***	-5.87***	-6.8 to -4.9
Pseudomonas								
TAP	-0.65**	-0.65**	-0.67***	-0.68***	-0.68***	-0.47*	-0.57***	-0.91 to -0.29
a_S	-1.34***	-1.33***	-1.20***	-1.01***	-1.00***	-0.94***	-0.93***	-1.46 to -0.46
eap						-0.21		
ppap	0.27	0.27						
non_D	-0.33							
los7				1.03***	1.03***	0.96***	0.97***	0.53 to 1.45
trauma50					0.04	0.03	0.02	-0.33 to 0.36
CC						0.56**	0.56**	0.08 to 1.10

Table S5: Development of GSEM model (continued) [a]

	Model 1	Model 2	Model 3	Model 4	Model 5	Model 7	Model 6	
	Fig S8	Fig S9	Fig S10	Fig S11	Fig S12	Fig S12	Fig S13	95%CI
**Factor** [b-j]								
**Acinetobacter**								
**TAP**	-0.25	-0.25	-0.27	-0.27	-0.5	-0.58	-0.43	-1.04 to 0.15
**a_S**	-1.26*	-1.27*	-1.21*	-1.04*	-0.85	-0.8	-0.82	-1.83 to 0.19
**eap**						0.25		
**ppap**	0.1	0.1						
**non_D**	0.06							
**los7**				1.15***	1.01***	0.99***	0.98***	0.41 to 1.54
**trauma50**					1.09***	1.04***	1.04***	0.47 to 1.62
**CC**						0.42	0.42	-0.22 to 1.22
**Error terms**								
**var(e.Ps_GO)**	1.32*	1.32*	1.17**	0.76**	0.76**	0.71**	0.72**	0.36 to 1.47
**var(e.Ac_GO)**	2.66***	2.66***	2.56***	1.92***	1.62***	1.60***	1.60***	1.01 to 2.48
**Model fit** [k]								
**AIC**	3345.94	3344.15	3329.29	3274.57	3261.55	3259.1	3255.53	
**N**	22	20	20	22	24	28	26	
**Groups (n)**	334	334	334	334	334	334	334	
**Clusters (n)**	213	213	213	213	213	213	213	

Footnotes

a. Legend: * p<0.05; ** p<0.01; *** p<0.001

b. v_ps_n is the count of *Pseudomonas* VAP; v_ac_n is the count of *Acinetobacter* VAP; b_ps_n is the count of *Pseudomonas* bacteremia and b_ac_n is the count of *Acinetobacter* bacteremia

c. PPAP is the group wide use of protocolized parenteral antibiotic prophylaxis; tap is topical antibiotic prophylaxis; eap is enteral antibiotic prophylaxis

d. Acinetobacter GO is the Acinteobacter gut overgrowth latent variable

e. Pseudomonas GO is the Pseudomonas gut overgrowth latent variable

f. MVP90 is use of mechanical ventilation by more than 90% of the group

g. LOS7 is a mean or median length of ICU stay for the group of 7 days or greater

h. Trauma ICU arbitrarily defined as an ICU for which >50% of admissions were for trauma

i. CC is concurrency of control groups with an intervention group receiving TAP

j. Less than 90% of the group receiving prolonged mechanical ventilation.

k. Model fit; AIC is Akaike's information criteria. This indicates model fit taking into account the statistical goodness of fit and the number of parameters in the model. Lower values of AIC indicate a better model fit. N is the number of parameters in the model.

Table S6: VAP count data [a]

	Observational studies	Infection prevention studies		
	(no intervention)	Non-dec	Anti-septic	TAP ±PPAP
Excluding groups with LOS<7 days				
Acinetobacter				
CC or observational groups	586/37026 [b, c]<br>1.6% (67)	30/2620 [b]<br>1.1% (25)	4/780 [b]<br>0.5% (5)	67/1521 [b]<br>4.4% (25)
Intervention groups		34/2429 [c]<br>1.4% (24)	8/786 [c]<br>1.0% (5)	41/1721 [c]<br>2.4% (26)
Pseudomonas				
CC or observational groups	2217/60131 [d, e]<br>3.7% (81)	200/4288 [d]<br>4.7% (38)	27/914 [d]<br>3.0% (8)	179/2161 [d]<br>8.3% (34)
Intervention groups		167/4169 [e]<br>4.0% (37)	24/1027 [e]<br>2.3% (8)	106/3193 [e]<br>3.3% (37)

Footnotes to table S6

a. Non-dec = Non-decontamination studies; TAP = Topical antibiotic prophylaxis; PPAP = Protocolized parenteral antibiotic prophylaxis.

b. The counts of *Acinetobacter* VAP among the three categories of control groups and the category of observation groups among studies after excluding those with length of stay <7 days differed significantly ($p < 0.001$; Fisher's exact test)

c. The counts of *Acinetobacter* VAP among the three categories of intervention groups and the category of observation groups among studies after excluding those with length of stay <7 days differed significantly ($p = 0.038$; Fisher's exact test)

d. The counts of *Pseudomonas* VAP among the three categories of control groups and the category of observation groups among studies after excluding those with length of stay <7 days differed significantly ($p < 0.001$; Fisher's exact test)

e. The counts of *Pseudomonas* VAP among the three categories of intervention groups and the category of observation groups among studies after excluding those with length of stay <7 days was differed marginally ($p = 0.05$; Fisher's exact test)

Table S7: Bacteremia count data [a]

	Observational studies	Infection prevention studies		
	(no intervention)	Non-dec	Anti-septic	TAP ±PPAP
**All groups**				
Acinetobacter				
CC or observational groups	203/189338<br>0.11% (20)	1/553<br>0.18% (2)	17/39162<br>0.04% (8)	15/1860<br>0.8% (13)
Intervention groups		1/526<br>0.19% (2)	7/57009<br>0.01% (8)	15/13290 [b]<br>0.11% (18)
Pseudomonas				
CC or observational groups	567/192203<br>0.30% (27)	2/553<br>0.36% (2)	23/39162<br>0.06% (8)	63/5280<br>1.2% (16)
Intervention groups		3/526<br>0.57% (2)	52/59117<br>0.09% (9)	139/23543<br>0.59% (25)
**Excluding groups with LOS<7 days**				
Acinetobacter				
CC or observational groups	37/12913 [c, d]<br>0.29% (11)	0/200 [c]<br>0% (1)	0/308 [c]<br>0% (3)	14/904 [c]<br>1.5% (11)
Intervention groups		1/199 [d]<br>0.5% (1)	1/305 [d]<br>0.33% (3)	11/1256 [d]<br>0.88% (14)
Pseudomonas				
CC or observational groups	111/14453 [e, f]<br>0.77% (16)	0/200 [e]<br>0% (1)	0/308 [e]<br>0.0% (3)	63/5249 [e]<br>1.2% (15)
Intervention groups		2/199 [f]<br>1.0% (1)	17/2413 [f]<br>0.7% (4)	94/12531 [f, g]<br>0.75% (22)

Footnotes to table S7

a. Non-dec = Non-decontamination studies; TAP = Topical antibiotic prophylaxis; PPAP = Protocolized parenteral antibiotic prophylaxis.
b. Among intervention groups of TAP based prevention studies, the count of *Acinetobacter* bacteremias was 12/6609 (0.18%; 13 studies) versus 3/6681 (0.04%; 4 studies) for those using versus not including PPAP in the intervention (p = 0.02, Fisher's exact test)

c. The counts of *Acinetobacter* bacteremias among the three categories of control groups and the category of observation groups among studies after excluding those with length of stay <7 days differed significantly ($p < 0.001$; Fisher's exact test)

d. The counts of *Acinetobacter* bacteremias among the three categories of intervention groups and the category of observation groups among studies after excluding those with length of stay <7 days differed significantly ($p = 0.012$; Fisher's exact test)

e. The counts of *Pseudomonas* bacteremias among the three categories of control groups and the category of observation groups among studies after excluding those with length of stay <7 days differed significantly ($p = 0.010$; Fisher's exact test)

f. The counts of *Pseudomonas* bacteremias among the three categories of intervention groups and the category of observation groups among studies after excluding those with length of stay <7 days was not significantly different ($p = 0.90$; Fisher's exact test)

g. Among intervention groups of TAP based prevention studies excluding those with a LOS less than 7 days, the count of *Pseudomonas* bacteremias was 53/5908 (0.9%; 16 studies) versus 41/6623 (0.62%; 6 studies) for those using versus not including PPAP in the intervention ($p = 0.07$, Fisher's exact test)

Details of methods used in additional analyses

Benchmarking: visual

Caterpillar plots were generated to facilitate a visual benchmark of the *Pseudomonas* bacteremia and *Acinetobacter* bacteremia incidence proportion and these were generated as follows. The data for each bacteremia incidence proportion were logit transformed to generate caterpillar plots using the 'metan' command in STATA (release 12.0, STATA Corp., College Station, TX, USA). For *Pseudomonas* bacteremia this transformation proceeds as follows; with the number of mechanically ventilated patients as the denominator (D), the number of patients with *Pseudomonas* bacteremia as the numerator (N), and R being the *Pseudomonas* bacteremia proportion (N/D), the logit(*Pseudomonas* bacteremia) is log(N/(D-N)) and its variance is 1/(D*R*(1-R)). Note that for any group with a zero event rate (N=0), the addition of the continuity correction (i.e. N+0·5) is required to avoid indeterminate transformations of the logit proportion and its variance. The visual benchmarks are the summary incidences for each of the *Pseudomonas* bacteremia and *Acinetobacter* bacteremia as derived using the observational studies. These visual benchmarks were then used in the respective caterpillar plots of the component groups from the VAP prevention studies as a reference line.

Dot plots were used to provide an 'at a glance' summary of the entire evidence base. These were derived as above for caterpillar plots but without the confidence limits. In dot plots, any group with a zero event rate (N=0) was arbitrarily assigned a low finite value (either 0.1%, 0.02% or 0.01%, depending on the spread of values). In the dot plots, a benchmark derived from the caterpillar plot as described above is used.

Meta-analysis: Effect sizes

The study specific and overall summary effect sizes and associated 95% confidence interval for each of the antibiotic, antiseptic and non-decontamination based interventions against either VAP overall or bacteremia overall were calculated using the Der Simonian-Laird random-effect methods of meta-analysis using the 'metan' command in Stata 14.2 (Stata Corp., College Station, TX, USA).

References

S1. A'Court CH, Garrard CS, Crook D, Bowler I, Conlon C, Peto T, Anderson E: Microbiological lung surveillance in mechanically ventilated patients, using non-directed bronchial lavage and quantitative culture. Q J Med. 1993;86:635-48.

S2. Alvarez-Lerma F, ICU-acquired Pneumonia Study Group. Modification of empiric antibiotic treatment in patients with pneumonia acquired in the intensive care unit. *Intens Care Med.* 1996;22(5):387-94.

S3. Antonelli M, Moro ML, Capelli O, De Blasi RA, D'Errico RR, Conti G, Bufi M, Gasparetto A: Risk factors for early onset pneumonia in trauma patients. Chest. 1994;105:224-228

S4. Apostolopoulou E, Bakakos P, Katostaras T, Gregorakos L: Incidence and risk factors for ventilator-associated pneumonia in 4 multidisciplinary intensive care units in Athens, Greece. Respir Care. 2003;48: 681-688.

S5. Baraibar J, Correa H, Mariscal D, Gallego M, Valles J, Rello J. Risk factors for infection by *Acinetobacter baumannii* in intubated patients with nosocomial pneumonia. *Chest*. 1997; 112:1050-1054.

S6. Beck-Sague CM, Sinkowitz RL, Chinn RY, Vargo J, Kaler W, Jarvis WR: Risk factors for ventilator-associated pneumonia in surgical intensive-care-unit patients. Infect Control Hosp Epidemiol. 1996;17:374-6.

S7. Bekaert M, Timsit JF, Vansteelandt S, Depuydt P, Vésin A, Garrouste-Orgeas M, Decruyenaere J, Clec'h C, Azoulay E, Benoit D. Attributable mortality of ventilator-associated pneumonia: a reappraisal using causal analysis. American journal of respiratory and critical care medicine. 2011;184(10):1133-9.

S8. Bercault N, Boulain T: Mortality rate attributable to ventilator-associated nosocomial pneumonia in an adult intensive care unit: a prospective case-control study. Crit Care Med. 2001;29:2303-2309

S9. Berrouane Y, Daudenthun I, Riegel B, Emery MN, Martin G, Krivosic R, Grandbastien B. Early onset pneumonia in neurosurgical intensive care unit patients. J Hosp Infect. 1998;40(4):275-80.

S10. Bochicchio GV, Joshi M, Bochicchio K, Tracy K, Scalea TM: A time-dependent analysis of intensive care unit pneumonia in trauma patients. J Trauma. 2004;56:296-301.

S11. Bonten MJ, Gaillard CA, van Tiel FH, Smeets HG, van der Geest S, Stobberingh EE: The stomach is not a source for colonization of the upper respiratory tract and pneumonia in ICU patients. Chest. 1994;105(3):878-84.

S12. Boots RJ, Phillips GE, George N, Faoagali JL. Surveillance culture utility and safety using low-volume blind bronchoalveolar lavage in the diagnosis of ventilator- associated pneumonia. Respirology. 2008;13:87-96.

S13. Boots RJ, Phillips GE, George N, Faoagali JL: Surveillance culture utility and safety using low-volume blind bronchoalveolar lavage in the diagnosis of ventilator- associated pneumonia. Respirology. 2008;13:87-96

S14. Bornstain C, Azoulay E, De Lassence A, Cohen Y, Costa MA, Mourvillier B, Descorps-Declere A, Garrouste-Orgeas M, Thuong M, Schlemmer B, Timsit JF: Sedation, sucralfate, and antibiotic use are potential means for protection against early-onset ventilator-associated pneumonia. Clin Infect Dis. 2004;38(10):1401-8.

S15. Braun SR, Levin AB, Clark KL. Role of corticosteroids in the development of pneumonia in mechanically ventilated head-trauma victims. Crit Care Med 1986;14:198-201

S16. Bregeon F, Papazian L, Visconti A, Gregoire R, Thirion X, Gouin F: Relationship of microbiologic diagnostic criteria to morbidity and mortality in patients with ventilator-associated pneumonia. JAMA. 1997;277: 655-662

S17. Bronchard R, Albaladejo P, Brezac G, et al. Early onset pneumonia: risk factors and consequences in head trauma patients. *Anesthesiology* 2004;100:234-9.

S18. Cade JF, McOwat E, Siganporia R, Keighley C, Presneill J, Sinickas V: Uncertain relevance of gastric colonization in the seriously ill. Intensive Care Med. 1992;18:210-217

S19. Cavalcanti M, Ferrer M, Ferrer R, Morforte R, Garnacho A, Torres A: Risk and prognostic factors of ventilator-associated pneumonia in trauma patients. Crit Care Med. 2006;34:1067-1072

S20. Cendrero JA, Solé-Violán J, Benitez AB, Catalán JN, Fernández JA, Santana PS, de Castro FR: Role of different routes of tracheal colonization in the development of pneumonia in patients receiving mechanical ventilation. Chest. 1999;116:462-470

S21. Chaari A, El Habib M, Ghdhoun H, Algia NB, Chtara K, Hamida CB, Chelly H, Bahloul M, Bouaziz M. Does low-dose hydrocortisone therapy prevent ventilator-associated pneumonia in trauma patients?. Am J Therap. 2015;22(1):22-8.

S22. Chastre J, Trouillet JL, Vuagnat A, Joly-Guillou ML, Clavier H, Dombret MC, Gibert C: Nosocomial pneumonia in patients with acute respiratory distress syndrome. Am J Respir Crit Care Med. 1998;157:1165-1172

S23. Chevret S, Hemmer M, Carlet J: Incidence and risk factors of pneumonia acquired in intensive care units. Results from a multicenter prospective study on 996 patients. European Cooperative Group on Nosocomial Pneumonia. Intensive Care Med. 1993;19:256-264

S24. Cook A, Norwood S, Berne J: Ventilator-associated pneumonia is more common and of less consequence in trauma patients compared with other critically ill patients. J Trauma Acute Care Surg. 2010;69(5):1083-91.

S25. Craven DE, Kunches LM, Lichtenberg DA, Kollisch NR, Barry MA, Heeren TC, McCabe WR: Nosocomial infection and fatality in medical and surgical intensive care unit patients. Arch Intern Med. 1988;148:1161-1168

S26. Daschner F, Kappstein I, Schuster F, Scholz R, Bauer E, Jooβens D, Just H: Influence of disposable ('Conchapak') and reusable humidifying systems on the incidence of ventilation pneumonia. J Hosp Infect. 1988;11:161-168

S27. De Latorre FJ, Pont T, Ferrer A, Rosselló J, Palomar M, Planas M: Pattern of tracheal colonization during mechanical ventilation. Am J Respir Crit Care Med. 1995;152:1028-1033

S28. El-Masri MM, Hammad TA, McLeskey SW, Joshi M, Korniewicz DM. Predictors of nosocomial bloodstream infections among critically ill adult trauma patients. *Infect Cont & Hosp Epidemiol.* 2004;25(8):656-63.

S29. Ensminger SA, Wright RS, Baddour LM, Afess B: Suspected ventilator-associated pneumonia in cardiac patients admitted to the coronary care unit. Mayo Clin Proc. 2006;81:32–35

S30. Esteve F, Pujol M, Limon E, Saballs M, Argerich MJ, Verdaguer R, Manez R, Ariza X, Gudiol F. Bloodstream infection related to catheter connections: a prospective trial of two connection systems. *J Hosp Infect*. 2007;67(1):30-4.

S31. Evans HL, Zonies DH, Warner KJ, Bulger EM, Sharar SR, Maier RV, Cuschieri J. Timing of intubation and ventilator-associated pneumonia following injury. Arch Surg. 2010;145(11):1041-6.

S32. Ewig S, Torres A, El-Ebiary M, Fàbregas N, Hernandez C, Gonzalez J, Nicolas JM, Soto L: Bacterial colonization patterns in mechanically ventilated patients with traumatic and medical head injury. Incidence, risk factors, and association with ventilator-associated pneumonia. Am J Respir Crit Care Med. 1999;159:188-198

S33. Fabian TC, Boucher BA, Croce MA, Kuhl DA, Janning SW, Coffey BC, Kudsk KA: Pneumonia and stress ulceration in severely injured patients: a prospective evaluation of the effects of stress ulcer prophylaxis. Arch Surg. 1993;128(2):185-92.

S34. Fagon JY, Chastre J, Domart Y, Trouillet JL, Pierre J, Darne C, Gibert C: Nosocomial pneumonia in patients receiving continuous mechanical ventilation. Prospective analysis of 52 episodes

with use of a protected specimen brush and quantitative culture techniques. *Am Rev Respir Dis* 1989;139:877-884.

S35. Gacouin A, Barbarot N, Camus C, Salomon S, Isslame S, Marque S, Lavoué S, Donnio PY, Thomas R, Le Tulzo Y. Late-onset ventilator-associated pneumonia in nontrauma intensive care unit patients. Anesth Analg. 2009;109(5):1584-90.

S36. Garrouste-Orgeas M, Chevret S, Arlet G, Marie O, Rouveau M, Popoff N, Schlemmer B: Oropharyngeal or gastric colonization and nosocomial pneumonia in adult intensive care unit patients. A prospective study based on genomic DNA analysis. Am J Respir Crit Care Med. 1997;156(5):1647-56.

S37. Garrouste-Orgeas M, Timsit JF, Tafflet M, Misset B, Zahar JR, Soufir L, Carlet J: Excess risk of death from intensive care unit—acquired nosocomial bloodstream infections: a reappraisal. *Clin Infect Dis* 2006, 42:1118-1126.

S38. George DL, Falk PS, Wunderink RG, Leeper Jr KV, Meduri GU, Steere EL, Glen Mayhall C: Epidemiology of ventilator-acquired pneumonia based on protected bronchoscopic sampling. Am J Respir Crit Care Med. 1998;158:1839-1847

S39. Georges H, Leroy O, Guery B, Alfandari S, Beaucaire G: Predisposing factors for nosocomial pneumonia in patients receiving mechanical ventilation and requiring tracheotomy. Chest. 2000;118:767–774.

S40. Giamarellos-Bourboulis EJ, Bengmark S, Kannellakopoulou K, Kotzampassi K: Pro- and synbiotics to control inflammation and infection in patients with multiple injuries. *J Trauma* 2009;67:815-821.

S41. Giard M, Lepape A, Allaouchiche B, Guerin C, Lehot JJ, Robert MO, Vanhems P: Early-and late-onset ventilator-associated pneumonia acquired in the intensive care unit: comparison of risk factors. *J Crit Care* 2008, 23:27-33.

S42. Gruson D, Hilbert G, Vargas F, Valentino R, Bebear C, Allery A, Bebear C, Gbikpi-Benissan GE, Cardinaud JP: Rotation and restricted use of antibiotics in a medical intensive care unit: impact on the incidence of ventilator-associated pneumonia caused by antibiotic-resistant gram-negative bacteria. Am J Respir Crit Care Med. 2000, 162(3):837-43.

S43. Gruson D, Hilbert G, Vargas F, Valentino R, Bui N, Pereyre S, Bebear C, Bebear CM, Gbikpi-Benissan G: Strategy of antibiotic rotation: long-term effect on incidence and susceptibilities of Gram-negative bacilli responsible for ventilator-associated pneumonia. Crit Care Med. 2003;31:1908-1914.

S44. Guérin C, Girard R, Chemorin C, De Varax R, Fournier G: Facial mask noninvasive mechanical ventilation reduces the incidence of nosocomial pneumonia. Intens care Med. 1997;23(10):1024-32.

S45. Heyland DK, Cook DJ, Schoenfeld PS, Frietag A, Varon J, Wood G: The effect of acidified enteral feeds on gastric colonization in critically ill patients: results of a multicenter randomized trial. Canadian Critical Care Trials Group. Crit Care Med. 1999;27:2399-2406

S46. Holzapfel L, Chevret S, Madinier G, Ohen F, Demingeon G, Coupry A, Chaudet M: Influence of long-term oro- or nasotracheal intubation on nosocomial maxillary sinusitis and pneumonia: results of a prospective, randomized, clinical trial. Crit Care Med. 1993;21:1132-1138

S47. Hortal J, Giannella M, Pérez MJ, Barrio JM, Desco M, Bouza E, Muñoz P. Incidence and risk factors for ventilator-associated pneumonia after major heart surgery. Intens care med. 2009;35(9):1518-25.

S48. Huang SS, Septimus E, Kleinman K, Moody J, Hickok J, Avery TR, Lankiewicz J, Gombosev A, Terpstra L, Hartford F, Hayden MK. Targeted versus universal decolonization to prevent ICU infection. *N Engl J Med*. 2013;368(24):2255-65.

S49. Hugonnet S, Uçkay I, Pittet D Staffing level: a determinant of late-onset ventilator-associated pneumonia. Crit Care. 2007;11(4):R80

S50. Hyllienmark P, Brattström O, Larsson E, Martling CR, Petersson J, Oldner A: High incidence of post- injury pneumonia in intensive care- treated trauma patients. Acta Anaesthesiologica Scandinavica. 2013;57(7):848-54.

S51. Ibáñez J, Peñafiel A, Marsé P, Jordá R, Raurich JM, Mata F: Incidence of gastroesophageal reflux and aspiration in mechanically ventilated patients using small-bore nasogastric tubes. J Parenteral and Enteral Nutrition. 2000;24(2):103-6.

S52. Ibrahim EH, Ward S, Sherman G, Kollef MH: A comparative analysis of patients with early-onset vs late-onset nosocomial pneumonia in the ICU setting. Chest. 2000;117:1434-1442

S53. Jaillette E, Nseir S: Relationship between inhaled β2-agonists and ventilator-associated pneumonia: A cohort study. Critical Care Med. 2011;39(4):725-30.

S54. Jiménez P, Torres A, Rodríguez-Roisin R, de la Bellacasa JP, Aznar R, Gatell JM, Agustí-Vidal A: Incidence and etiology of pneumonia acquired during mechanical ventilation. Crit Care Med. 1989;17:882-5.

S55. Kallel H, Chelly H, Bahloul M, Ksibi H, Dammak H, Chaari A, Hamida CB, Rekik N, Bouaziz M. The effect of ventilator-associated pneumonia on the prognosis of head trauma patients. J Trauma Acute Care Surg. 2005;59(3):705-10.

S56. Ko HK, Yu WK, Lien TC, Wang JH, Slutsky AS, Zhang H, Kou YR. Intensive care unit-acquired bacteremia in mechanically ventilated patients: clinical features and outcomes. *PloS one*. 2013;8(12):e83298.

S57. Kollef MH: Ventilator-associated pneumonia. A multivariate analysis. JAMA. 1993;270:1965-70.

S58. Kollef MH, Silver P, Murphy DM, Trovillion E: The effect of late-onset ventilator-associated pneumonia in determining patient mortality. Chest. 1995;108: 1655-62.

S59. Kollef MH, Von Harz B, Prentice D, Shapiro SD, Silver P, John RS, Trovillion E: Patient transport from intensive care increases the risk of developing ventilator-associated pneumonia. Chest. 1997;112(3):765-773.

S60. Kollef MH, Chastre J, Fagon JY, François B, Niederman MS, Rello J, Torres A, Vincent JL, Wunderink RG, Go KW, Rehm C. Global prospective epidemiologic and surveillance study of ventilator-associated pneumonia due to Pseudomonas aeruginosa. Crit care med. 2014;42(10):2178-87.

S61. Koss WG, Khalili TM, Lemus JF, Chelly MM, Margulies DR, Shabot MM: Nosocomial pneumonia is not prevented by protective contact isolation in the surgical intensive care unit. Am Surg. 2001;67:1140-4.

S62. Kunac A, Sifri ZC, Mohr AM, Horng H, Lavery RF, Livingston DH: Bacteremia and Ventilator-Associated Pneumonia: A Marker for Contemporaneous Extra-Pulmonic Infection. Surg Infect. 2014;15:77-83.

S63. Laggner AN, Lenz K, Base W, Druml W, Schneeweiss B, Grimm G: Prevention of upper gastrointestinal bleeding in long-term ventilated patients. Sucralfate versus ranitidine. Am J Med. 1989;86:81-4.

S64. Lambert ML, Suetens C, Savey A, Palomar M, Hiesmayr M, Morales I, Agodi A, Frank U, Mertens K, Schumacher M, Wolkewitz M. Clinical outcomes of health-care-associated infections and antimicrobial resistance in patients admitted to European intensive-care units: a cohort study. Lancet Infect Dis. 2011;11(1):30-8.

S65. Laupland KB, Zygun DA, Davies HD, Church DL, Louie TJ, Doig CJ Population-based assessment of intensive care unit-acquired bloodstream infections in adults: incidence, risk factors, and associated mortality rate. *Crit Care Med* 2002;30:2462-2467.

S66. Laupland KB, Kirkpatrick AW, Church DL, Ross T, Gregson DB Intensive-care-unit-acquired bloodstream infections in a regional critically ill population. *J Hosp Infect* 2004;58(2): 137-145.

S67. Lepelletier D, Roquilly A, Mahe PJ, Loutrel O, Champin P, Corvec S, Naux E, Pinaud M, Lejus C, Asehnoune K. Retrospective analysis of the risk factors and pathogens associated with early-onset ventilator-associated pneumonia in surgical-ICU head-trauma patients. J Neurosurg Anesthesiol. 2010;22(1):32-7.

S68. Luyt CE, Guérin V, Combes A, Trouillet JL, Ayed SB, Bernard M, Gibert C, Chastre J: Procalcitonin kinetics as a prognostic marker of ventilator-associated pneumonia. Am J Respir Crit Care Med. 2005;171:48-53.

S69. Magnason S, Kristinsson KG, Stefansson T, Erlendsdottir H, Jonsdottir K, Kristjansson M, Gudmundsson S: Risk factors and outcome in ICU- acquired infections. Acta Anaesthesiologica Scandinavica. 2008;52:1238-1245

S70. Magret M, Amaya-Villar R, Garnacho J, Lisboa T, Diaz E, DeWaele J, Deja M, Manno E, Rello J, EU-VAP/CAP Study Group: Ventilator-associated pneumonia in trauma patients is associated with lower mortality: results from EU-VAP study. J Trauma Acute Care Surg. 2010;69(4):849-854.

S71. Mahul P, Auboyer C, Jospe R, Ros A, Guerin C, el Khouri Z, Galliez M, Dumont A, Gaudin O: Prevention of nosocomial pneumonia in intubated patients respective role of mechanical subglottic secretions drainage and stress ulcer prophylaxis. Intensive Care Med. 1992;18:20-25

S72. Makris D, Manoulakas E, Komnos A, Papakrivou E, Tzovaras N, Hovas A, Zintzaras E, Zakynthinos E. Effect of pravastatin on the frequency of ventilator-associated pneumonia and on intensive care unit mortality: open-label, randomized study. Crit care med. 2011;39(11):2440-6.

S73. Markowicz P, Wolff M, Djedaini K, Cohen Y, Chastre J, Delclaux C: Multicenter prospective study of ventilator-associated pneumonia during acute respiratory distress syndrome. Incidence, prognosis, and risk factors. ARDS Study Group. Am J Respir Crit Care Med. 2000;161:1942-8.

S74. Moine P, Timsit JF, De Lassence A, Troché G, Fosse JP, Alberti C, Cohen Y: Mortality associated with late-onset pneumonia in the intensive care unit: results of a multi-center cohort study. Intensive Care Med. 2002;28:154-163

S75. Montecalvo MA, Steger KA, Farber HW: Nutritional outcome and pneumonia in critical care patients randomized to gastric versus jejunal tube feedings. The Critical Care Research Team. *Crit Care Med* 1992, 20:1377-1387.

S76. Myny D, Depuydt P, Colardyn F, Blot S: Ventilator-associated pneumonia in a tertiary care ICU analysis of risk factors for acquisition and mortality. Acta Clin Belg. 2005;60:114-121.

S77. Nguile-Makao M, Zahar JR, Français A, Tabah A, Garrouste-Orgeas M, Allaouchiche B, Goldgran-Toledano D, Azoulay E, Adrie C, Jamali S, Clec'h C. Attributable mortality of ventilator-associated pneumonia: respective impact of main characteristics at ICU admission and VAP onset using conditional logistic regression and multi-state models. Intens care med. 2010;36(5):781-9.

S78. Nielsen SL, Røder B, Magnussen P, Engquist A, Frimodt-møller N. Nosocomial pneumonia in an intensive care unit in a Danish university hospital: incidence, mortality and etiology. Scand J Infect Dis. 1992;24:65-70.

S79. Nseir S, Di Pompeo C, Soubrier S, Cavestri B, Jozefowicz E, Saulnier F, Durocher A: Impact of ventilator-associated pneumonia on outcome in patients with COPD. Chest. 2005;128(3):1650-1656.

S80. Osmon S, Warren D, Seiler SM, Shannon W, Fraser VJ, Kollef MH: The influence of infection on hospital mortality for patients requiring >48 h of intensive care. *Chest* 2003, 124:1021-1029.

S81. Papazian L, Bregeon F, Thirion X, Gregoire R, Saux P, Denis JP, Perin G, Charrel J, Dumon JF, Affray JP, Gouin F: Effect of ventilator-associated pneumonia on mortality and morbidity. Am J Respir Crit Care Med. 1996;154:91-7.

S82. Potgieter PD, Linton DM, Oliver S, Forder AA: Nosocomial infections in a respiratory intensive care unit. Crit Care Med. 1987;15:495-498

S83. Rello J, Quintana E, Ausina V, Castella J, Luquin M, Net A, Prats G: Incidence, etiology, and outcome of nosocomial pneumonia in mechanically ventilated patients. Chest. 1991;100:439-444

S84. Rello J, Ausina V, Castella J, et al Nosocomial respiratory tract infections in multiple trauma patients. Influence of level of consciousness with implications for therapy. Chest 1992;102:525-529

S85. Rello J, Ricart M, Mirelis B, Quintana E, Gurgui M, Net A, Prats, G: Nosocomial bacteremia in a medical-surgical intensive care unit: epidemiologic characteristics and factors influencing mortality in 111 episodes. *Intensive Care Med* 1994;20:94-98.

S86. Rello J, Ausina V, Ricart M, Puzo C, Quintana E, Net A, Prats G. Risk factors for infection by *Pseudomonas aeruginosa* in patients with ventilator-associated pneumonia. *Intens Care Med*. 1994;20(3):193-8.

S87. Rello J, Sonora R, Jubert P, et al. Pneumonia in intubated patients: role of respiratory airway Care *Am J Respir Crit Care Med* 1996;154:111-5.

S88. Rello J, Ollendorf DA, Oster G, et al. Epidemiology and outcomes of ventilator-associated pneumonia in a large US database. Chest 2002;122:2115-2121

S89. Rello J, Lorente C, Diaz E, et al. Incidence, etiology, and outcome of nosocomial pneumonia in ICU patients requiring percutaneous tracheotomy for mechanical ventilation. *Chest*. 2003;124:2239-2243.

S90. Reusser P, Zimmerli W, Scheidegger D, Marbet GA, Buser M, Gyr K: Role of gastric colonization in nosocomial infections and endotoxemia: a prospective study in neurosurgical patients on mechanical ventilation. J Infect Dis. 1989;160:414-421

S91. Rincón-Ferrari MD, Flores-Cordero JM, Leal-Noval SR, Murillo-Cabezas F, Cayuelas A, Muñoz-Sánchez MA, Sánchez-Olmedo JI: Impact of ventilator-associated pneumonia in patients with severe head injury. J Trauma Acute Care Surg. 2004;57(6):1234-40.

S92. Rodriguez JL, Gibbons KJ, Bitzer LG, Dechert RE, Steinberg SM, Flint LM: Pneumonia: incidence, risk factors, and outcome in injured patients. J Trauma. 1991;31: 907-12.

S93. Ruiz-Santana S, Garcia Jimenez A, Esteban A, et al. ICU pneumonias: a multi-institutional study. *Crit Care Med*. 1987;15:930-932.

S94. Salata RA, Lederman MM, Shlaes DM, Jacobs MR, Eckstein E, Tweardy D, Toossi Z, Chmielewski R, Marino J, King CH: Diagnosis of nosocomial pneumonia in intubated, intensive care unit patients. Am Rev Respir Dis. 1987;135:426-432

S95. Shahin J, Bielinski M, Guichon C, Flemming C, Kristof AS Suspected ventilator-associated respiratory infection in severely ill patients: a prospective observational study. Crit Care 2013;17(5): R251

S96. Sligl W, Taylor G, Brindley PG. Five years of nosocomial Gram-negative bacteremia in a general intensive care unit: epidemiology, antimicrobial susceptibility patterns, and outcomes. Int J Infect Dis. 2006;10(4):320-5.

S97. Sofianou DC, Constandinidis TC, Yannacou M, Anastasiou H, Sofianos E: Analysis of risk factors for ventilator-associated pneumonia in a multidisciplinary intensive care unit. *Eur J Clin Microbiol Infect Dis* 2000, 19:460-463.

S98. Stéphan F, Mabrouk N, Decailliot F, Delclaux C, Legrand P: Ventilator-associated pneumonia leading to acute lung injury after trauma: importance of *Haemophilus influenzae*. Anesthesiology. 2006;104: 235-41.

S99. Tejada Artigas AT, Dronda SB, Vallés EC, Marco JM, Usón MC, Figueras P, Suarez FJ, Hernandez A: Risk factors for nosocomial pneumonia in critically ill trauma patients. Crit Care Med. 2001;29:304-9.

S100. Thompson DS. Estimates of the rate of acquisition of bacteraemia and associated excess mortality in a general intensive care unit: a 10 year study. *J Hosp Infect*. 2008;69(1):56-61.

S101. Timsit JF, Chevret S, Valcke J, Misset B, Renaud B, Goldstein FW, Vaury P, Carlet J: Mortality of nosocomial pneumonia in ventilated patients: influence of diagnostic tools. Am J Respir Crit Care Med. 1996;154:116-23.

S102. Torres A, Aznar R, Gatell JM, Jiménez P, González J, Ferrer A, Celis R, Rodriguez-Roisin R: Incidence, risk, and prognosis factors of nosocomial pneumonia in mechanically ventilated patients. Am Rev Respir Dis. 1990;142:523-8.

S103. Trouillet JL, Chastre J, Vuagnat A, Joly-Guillou ML, Combaux D, Dombret MC, Gibert C: Ventilator-associated pneumonia caused by potentially drug-resistant bacteria. Am J Respir Crit Care Med. 1998;157(2):531-9.

S104. Urli T, Perone G, Acquarolo A, Zappa S, Antonini B, Ciani A: Surveillance of infections acquired in intensive care: usefulness in clinical practice. *J Hosp Infect* 2002, 52:130-5.

S105. Valles J, Pobo A, Garcia-Esquirol O, Mariscal D, Real J, Fernández R. Excess ICU mortality attributable to ventilator-associated pneumonia: the role of early vs late onset. *Intensive care medicine*, 2007;33(8):1363-1368.

S106. Vanhems P, Bénet T, Voirin N, Januel JM, Lepape A, Allaouchiche B, Argaud L, Chassard D, Guérin C. Early-onset ventilator-associated pneumonia incidence in intensive care units: a surveillance-based study. BMC Infect Dis. 2011;11(1):236.

S107. Verhamme KM, De Coster W, De Roo L, De Beenhouwer H, Nollet G, Verbeke J, Demeyer I, Jordens P: Pathogens in early-onset and late-onset intensive care unit–acquired pneumonia. Infection Control Hospital Epidemiol. 2007;28(4):389-397.

S108. Violan JS, Sanchez-Ramirez C, Mujica AP, Cendrero JC, Fernandez JA, de Castro FR: Impact of nosocomial pneumonia on the outcome of mechanically-ventilated patients. Crit Care (Lond). 1998;2:19-23.

S109. Warren DK, Zack JE, Elward AM, Cox MJ, Fraser VJ. Nosocomial primary bloodstream infections in intensive care unit patients in a nonteaching community medical center: a 21-month prospective study. *Clin Infect Dis.* 2001;33(8):1329-35.

S110. Woske HJ, Röding T, Schulz I, Lode H: Ventilator-associated pneumonia in a surgical intensive care unit Epidemiology, etiology and comparison of three bronchoscopic methods for microbiological specimen sampling. Crit Care. 2001;5:167–173.

S111. Zahar JR, Nguile-Makao M, Français A, Schwebel C, Garrouste-Orgeas M, Goldgran-Toledano D, Azoulay E, Thuong M, Jamali S, Cohen Y, De Lassence A. Predicting the risk of documented ventilator-associated pneumonia for benchmarking: construction and validation of a score. Crit care med. 2009;37(9):2545-51.

S112. Acosta-Escribano J, Fernández-Vivas M, Carmona TG, Caturla-Such J, Garcia-Martinez M, Menendez-Mainer A, Sanchez-Payá J (2010) Gastric versus transpyloric feeding in severe traumatic brain injury: a prospective, randomized trial. Intensive Care Med 36:1532-1539

S113. Bonten MJ, Gaillard CA, Van der Geest S, Van Tiel FH, Beysens AJ, Smeets HG, Stobberingh EE: The role of intragastric acidity and stress ulcer prophylaxis on colonization and infection in mechanically ventilated ICU patients. A stratified, randomized, double-blind study of sucralfate versus antacids. Am J Respir Crit Care Med. 1995;152:1825-1834.

S114. Combes P, Fauvage B, Oleyer C. Nosocomial pneumonia in mechanically ventilated patients, a prospective randomised evaluation of the Stericath closed suctioning system. *Intensive Care Med* 2000;26:878-82.

S115. Cook D, Guyatt G, Marshall J, et al A comparison of sucralfate and ranitidine for the prevention of upper gastrointestinal bleeding in patients requiring mechanical ventilation. Canadian Critical Care Trials Group. N Engl J Med 1998;338:791-797

S116. Dreyfuss D, Djedaini K, Weber P, Brun P, Lanore JJ, Rahmani J, Coste F: Prospective study of nosocomial pneumonia and of patient and circuit colonization during mechanical ventilation with circuit changes every 48 hours versus no change. Am Rev Respir Dis. 1991;143(4 Pt 1), 738-743.

S117. Daumal F, Colpart E, Manoury B, Mariani M, Daumal M. Changing heat and moisture exchangers every 48 hours does not increase the incidence of nosocomial pneumonia. Infection Control & Hospital Epidemiology. 1999;20(5):347-9.

S118. Drakulovic MB, Torres A, Bauer TT, Nicolas JM, Nogué S, Ferrer M: Supine body position as a risk factor for nosocomial pneumonia in mechanically ventilated patients: a randomised trial. Lancet. 1999;354(9193):1851-1858

S119. Dreyfuss D, Djedaïni K, Gros I, Mier L, Le Bourdellés G, Cohen Y, Estagnasié P, Coste F, Boussougant Y: Mechanical ventilation with heated humidifiers or heat and moisture exchangers: effects on patient colonization and incidence of nosocomial pneumonia. Am J Respir Crit Care Med. 1995;151:986-92.

S120. Driks MR, Craven DE, Celli BR, et al (1987) Nosocomial pneumonia in intubated patients given sucralfate as compared with antacids or histamine type 2 blockers. The role of gastric colonization. N Engl J Med 317:1376-1382

S121. Forestier C, Guelon D, Cluytens V, Guillart T, Sirot J, De champs C: Oral probiotic and prevention of *Pseudomonas aeruginosa* infections: a randomized, double-blind, placebo controlled pilot study in intensive care unit patients. *Crit Care* 2008;12:R69.

S122. Heyland DK, Cook DJ, Schoenfeld PS, Frietag A, Varon J, Wood G The effect of acidified enteral feeds on gastric colonization in critically ill patients: results of a multicenter randomized trial. Canadian Critical Care Trials Group. Crit Care Med 1999;27:2399-2406

S123. Holzapfel L, Chastang C, Demingeon G, Bohe J, Piralla B, Coupry A: A randomized study assessing the systematic search for maxillary sinusitis in nasotracheally mechanically ventilated patients. Influence of nosocomial maxillary sinusitis on the occurrence of ventilator-associated pneumonia. Am J Respir Crit Care Med. 1999;159:695-701

S124. Kirschenbaum L, Azzi E, Sfeir T, et al. Effect of continuous lateral rotational therapy on the prevalence of ventilator-associated pneumonia in patients requiring long-term ventilatory care *Crit Care Med* 2002;30:1983-6.

S125. Kirton OC, DeHaven B, Morgan J, et al. A prospective, randomized comparison of an in-line heat moisture exchange filter and heated wire humidifiers: rates of ventilator-associated early-onset (community-acquired) or late-onset (hospital-acquired) pneumonia and incidence of endotracheal tube occlusion. *Chest* 1997;112:1055-9.

S126. Knight DJ, Gardiner D, Banks A, Snape SE, Weston VC, Bengmark S, Girling KJ: Effect of synbiotic therapy on the incidence of ventilator associated pneumonia in critically ill patients: a randomised, double-blind, placebo-controlled trial. Intensive Care Med. 2009;35:854-861.

S127. Kollef MH, Vlasnik J, Sharpless L, Pasque C, Murphy D, Fraser V (1997) Scheduled change of antibiotic classes: A strategy to decrease the incidence of ventilator-associated pneumonia. Am J Respir Crit Care Med 156:1040–1048

S128. Kollef MH, Afessa B, Anzueto A, Veremakis C, Kerr KM, Margolis BD, Schinner R: Silver-coated endotracheal tubes and incidence of ventilator-associated pneumonia: the NASCENT randomized trial. JAMA. 2008;300(7):805-813

S129. Kortbeek JB, Haigh PI, Doig C. Duodenal versus gastric feeding in ventilated blunt trauma patients: a randomized controlled trial. *J Trauma* 1999;46:992-6.

S130. Lacherade JC, Auburtin M, Cerf C, Van de Louw A, Soufir L, Rebufat Y, Rezaiguia S, Ricard JD, Lellouche F, Brun-Buisson C, Brochard L: Impact of humidification systems on ventilator-associated pneumonia: a randomized multicenter trial. Am J Respir Crit Care Med. 2005;172:1276-1282

S131. Lacherade JC, De Jonghe B, Guezennec P, Debbat K, Hayon J, Monsel A, Bastuji-Garin S: Intermittent subglottic secretion drainage and ventilator-associated pneumonia A multicenter trial. Am J Respir Crit Care Med. 2010;182:910-917.

S132. Launey Y, Nesseler N, Le Cousin A, Feuillet F, Garlantezec R, Mallédant Y, Seguin P: Effect of a fever control protocol-based strategy on ventilator-associated pneumonia in severely brain-injured patients. Crit Care. 2014;18(6):1.

S133. Lorente L, Lecuona M, Málaga J, Revert C, Mora ML, Sierra A: Bacterial filters in respiratory circuits: an unnecessary cost? Crit Care Med 2003;31:2126-2130

S134. Lorente L, Lecuona M, Galván R, Ramos MJ, Mora ML, Sierra A: Periodically changing ventilator circuits is not necessary to prevent ventilator-associated pneumonia when a heat and moisture exchanger is used. Infect Control Hosp Epidemiol. 2004;25:1077-1082

S135. Lorente L, Lecuona M, Martín MM, García C, Mora ML, Sierra A: Ventilator-associated pneumonia using a closed versus an open tracheal suction system. Crit Care Med. 2005;33:115-119

S136. Lorente L, Lecuona M, Jiménez A, Mora ML, Sierra A: Tracheal suction by closed system without daily change versus open system. Intensive Care Med. 2006;32:538-44.

S137. Lorente L, Lecuona M, Jimenez A, Mora ML, Sierra A: Ventilator-associated pneumonia using a heated humidifier or a heat and moisture exchanger: a randomized controlled trial [ISRCTN88724583]. Crit Care 2006;10:R116

S138. Lorente L, Lecuona M, Jimenez A, Mora ML, Sierra: Influence of an endotracheal tube with polyurethane cuff and subglottic secretion drainage on pneumonia. Am J Respir Crit Care Med. 2007;176:1079-1083

S139. Lorente L, Lecuona M, Jiménez A, Lorenzo L, Roca I, Cabrera J, Llanos C, Mora ML: Continuous endotracheal tube cuff pressure control system protects against ventilator-associated pneumonia. Crit Care. 2014;18(2):1.

S140. Manzano F, Fernandez-Mondejar E, Colmenero M, Poyatos ME, Rivera R, Machado J, Catalan I, Artigas A: Positive-end expiratory pressure reduces incidence of ventilator-associated pneumonia in nonhypoxemic patients. Crit Care Med: 2008;36(8):2225-31.

S141. Martin C, Perrin G, Gevaudan MJ, Saux P, Gouin F. Heat and moisture exchangers and vaporizing humidifiers in the intensive care unit. Chest. 1990;97(1):144-9.

S142. Morrow LE, Kollef MH, Casale TB: Probiotic prophylaxis of ventilator-associated pneumonia: a blinded, randomized, controlled trial. Am J Respir Crit Care Med. 2010;182:1058-1064

S143. Nseir S, Zerimech F, Fournier C, Lubret R, Ramon P, Durocher A, Balduyck M: Continuous control of tracheal cuff pressure and microaspiration of gastric contents in critically ill patients. Am J Respir Crit Care Med. 2011;184(9):1041-7.

S144. Pickworth KK, Falcone RE, Hoogeboom JE, et al Occurrence of nosocomial pneumonia in mechanically ventilated trauma patients: a comparison of sucralfate and ranitidine. Crit Care Med 1993;21:1856-1862

S145. Pneumatikos I, Konstantonis D, Tsagaris I, Theodorou V, Vretzakis G, Danielides V, Bouros D: Prevention of nosocomial maxillary sinusitis in the ICU: the effects of topically applied alpha-adrenergic agonists and corticosteroids. Intensive Care Med. 2006;32:532-537

S146. Prod'hom G, Leuenberger P, Koerfer J, Blum A, Chiolero R, Schaller MD, Perret C, Spinnler O, Blondel J, Siegrist H, Saghafi L: Nosocomial pneumonia in mechanically ventilated patients receiving antacid, ranitidine, or sucralfate as prophylaxis for stress ulcer. A randomized controlled trial. Ann Intern Med. 1994;120:653-62.

S147. Reignier J, Mercier E, Le Gouge A, Boulain T, Desachy A, Bellec F, Lascarrou JB: Effect of Not Monitoring Residual Gastric Volume on Risk of Ventilator-Associated Pneumonia in Adults Receiving

Mechanical Ventilation and Early Enteral Feeding. A Randomized Controlled Trial. *JAMA* 2013, 309;249-256.

S148. Ryan P, Dawson J, Teres D, Celoria G, Navab F: Nosocomial pneumonia during stress ulcer prophylaxis with cimetidine and sucralfate. Arch Surg. 1993;128(12):1353-7.

S149. Smulders K, van der Hoeven H, Weers-Pothoff I, Vandenbroucke-Grauls C A randomized clinical trial of intermittent subglottic secretion drainage in patients receiving mechanical ventilation. Chest 2002;121:858-862

S150. Staudinger T, Bojic A, Holzinger U, Meyer B, Rohwer M, Mallner F, Locker GJ Continuous lateral rotation therapy to prevent ventilator-associated pneumonia Crit Care Med 2010;38(2):486-490

S151. Thomachot L, Viviand X, Arnaud S, Boisson C, Martin CD: Comparing two heat and moisture exchangers, one hydrophobic and one hygroscopic, on humidifying efficacy and the rate of nosocomial pneumonia. Chest. 1998;114:1383-1389

S152. Thomachot L, Leone M, Razzouk K, Antonini F, Vialet R, Martin C: Do the components of heat and moisture exchanger filters affect humidifying efficacy and the incidence of nosocomial pneumonia? Crit Care Med. 1999;27:923–928

S153. Thomachot L, Leone M, Razzouk K, Antonini F, Vialet R, Martin C: Randomized Clinical Trial of Extended Use of a Hydrophobic Condenser Humidifier: 1 vs 7 Days. Crit Care Med. 2002;30:232-7

S154. Valencia M, Ferrer M, Farre R, Navajas D, Badia JR, Nicolas JM, Torres A: Automatic control of tracheal tube cuff pressure in ventilated patients in semirecumbent position: a randomized trial. Crit Care Med. 2007;35: 1543-9.

S155. Valles J, Artigas A, Rello J, et al. Continuous aspiration of subglottic secretions in preventing ventilator-associated pneumonia. *Ann Intern Med* 1995;122:179-86.

S156. Walaszek M, Gniadek A, Kolpa M, Wolak Z, Kosiarska A. The effect of subglottic secretion drainage on the incidence of ventilator associated pneumonia. *Biomed Pap Med Fac Univ Palacky Olomouc Czech Repub*. 2017;161(4):374-80.

S157. Ćabov T, Macan D, Husedžinović I, Škrlin-Šubić J, Bošnjak D, Šestan-Crnek S, Perić B, Kovač Z, Golubović V. The impact of oral health and 0.2% chlorhexidine oral gel on the prevalence of nosocomial infections in surgical intensive-care patients: a randomized placebo-controlled study. Einfluss von Mundgesundheit und von 0, 2% Chlorhexidin-Gel auf die Entwicklung von nosokomialen Infektionen bei Patienten auf einer chirurgischen Intensivstation. Wiener klinische Wochenschrift. 2010;122(13-14):397-404.

S158. Climo MW, Yokoe DS, Warren DK et al. Effect of daily chlorhexidine bathing on hospital-acquired infection. *N Engl J Med* 2013; 368: 533–542.

S159. Fourrier FE, Cau-Pottier H, Boutigny M, Roussel-Delvallez M, Jourdain, Chopin C: Effects of dental plaque antiseptic decontamination on bacterial colonization and nosocomial infections in critically ill patients. Intensive Care Med. 2000;26:1239-1247

S160. Fourrier F, Dubois D, Pronnier P, Herbecq P, Leroy O, Desmettre T, Roussel-Delvallez M: Effect of gingival and dental plaque antiseptic decontamination on nosocomial infections acquired in the intensive care unit a double-blind placebo-controlled multicenter study. Crit Care Med. 2005;33:1728-1735

S161. Koeman M, van der Ven AJ, Hak E, et al. Oral decontamination with chlorhexidine reduces the incidence of ventilator-associated pneumonia. Am J Respir Crit Care Med 2006;173:1348-1355

S162. Kollef M, Pittet D, Sanchez Garcia M, et al. A randomized double-blind trial of iseganan in prevention of ventilator-associated pneumonia. *Am J Respir Crit Care Med* 2006: 173:91-7.

S163. Lorente L, Lecuona M, Jiménez A, Palmero S, Pastor E, Lafuente N, Ramos MJ, Mora ML, Sierra A: Ventilator-associated pneumonia with or without toothbrushing a randomized controlled trial. Eur J Clin Microbiol Infect Dis. 2012;31:1-9

S164. Noto MJ, Domenico HJ, Byrne DW, Talbot T, Rice TW, Bernard GR, Wheeler AP. Chlorhexidine bathing and health care–associated infections: a randomized clinical trial. *JAMA*. 2015;313(4):369-78.

S165. Seguin P, Tanguy M , Laviolle B, Tirel O, Malledant Y: Effect of oropharyngeal decontamination by povidone-iodine on ventilator-associated pneumonia in patients with head trauma. *Crit Care Med* 2006, 34:1514-1519.

S166. Seguin P, Laviolle B, Dahyot-Fizelier C, Dumont R, Veber B, Gergaud S, Asehnoune K, Mimoz O, Donnio PY, Bellissant E, Malledant Y. Effect of oropharyngeal povidone-iodine preventive oral care on ventilator-associated pneumonia in severely brain-injured or cerebral hemorrhage patients: a multicenter, randomized controlled trial. Crit care med. 2014;42(1):1-8.

S167. Swan JT, Ashton CM, Bui LN, Pham VP, Shirkey BA, Blackshear JE, Bersamin JB, Pomer RM, Johnson ML, Magtoto AD, Butler MO. Effect of chlorhexidine bathing every other day on prevention of hospital-acquired infections in the surgical ICU: a single-center, randomized controlled trial. Crit Care Med. 2016;44(10):1822-32.

S168. Wittekamp BH, Plantinga NL, Cooper BS, Lopez-Contreras J, Coll P, Mancebo J, Wise MP, Morgan MP, Depuydt P, Boelens J, Dugernier T. Decontamination strategies and bloodstream infections with antibiotic-resistant microorganisms in ventilated patients: a randomized clinical trial. *JAMA*. 2018.

S169. Bergmans DC, Bonten MJ, Gaillard CA, et al Prevention of ventilator-associated pneumonia by oral decontamination: a prospective, randomized, double-blind, placebo-controlled study. Am J Respir Crit Care Med 2001;164:382-388

S170. Bonten MJ, Gaillard CA, Johanson Jr WG, Van Tiel FH, Smeets HG, Van Der Geest S, Stobberingh EE. Colonization in patients receiving and not receiving topical antimicrobial prophylaxis. *Am J Respir Crit Care Med* 1994;150(5):1332-1340.

S171. Camus C, Salomon S, Bouchigny C, Gacouin A, Lavoué S, Donnio PY, Javaudin L, Chapplain JM, Uhel F, Le Tulzo Y, Bellissant E. Short-term decline in all-cause acquired infections with the routine use of a decontamination regimen combining topical polymyxin, tobramycin, and amphotericin B with mupirocin and chlorhexidine in the ICU: a single-center experience. *Crit Care Med.* 2014;42(5):1121-30.

S172. de Smet AMGA, Kluytmans JAJW, Cooper BS, Mascini EM, Benus RFJ, van der Werf TS, van der Hoeven JG, Pickkers P, Bogaers-Hofman D, van der Meer NJ, Bernards AT, Kuijper EJ, Joore JC, Leverstein-van Hall MA, Bindels AJ, Jansz AR, Wesselink RM, de Jongh BM, Dennesen PJ, van Asselt GJ, te Velde LF, Frenay IH, Kaasjager K, Bosch FH, van Iterson M, Thijsen SF, Kluge GH, Pauw W, de Vries JW, Kaan JA, Arends JP, Aarts LP, Sturm PD, Harinck HI, Voss A, Uijtendaal EV, Blok HE, Thieme Groen ES, Pouw ME, Kalkman CJ, Bonten MJ: Decontamination of the digestive tract and oropharynx in ICU patients. *N Engl J Med* 2009, 360:20–31.

S173. Godard J, Guillaume C, Reverdy ME, Bachmann P, Bui-Xuan B, Nageotte A, Motin J: Intestinal decontamination in a polyvalent ICU. A double-blind study. *Intensive Care Med* 1990, 16:307-311.

S174. Hartenauer U, Thulig B, Diemer W, Lawin P, Fegeler W, Kehrel R, Ritzerfeld W Effect of selective flora suppression on colonization, infection, and mortality in critically ill patients: a one-year, prospective consecutive study. *Crit Care Med* 1991;19:463-473

S175. Oostdijk EA, de Smet AM, Kesecioglu J, Bonten MJ. The role of intestinal colonization with gram-negative bacteria as a source for intensive care unit-acquired bacteremia. *Crit Care. Med.* 2011;39(5):961-6.

S176. Stoutenbeek CP, Van Saene HK, Miranda DR, Zandstra DF. The effect of selective decontamination of the digestive tract on colonisation and infection rate in multiple trauma patients. Intens Care Med. 1984;10(4):185-92.

S177. Stoutenbeek CP, van Saene HK, Miranda DR, Zandstra DF, Langrehr D; The effect of oropharyngeal decontamination using topical nonabsorbable antibiotics on the incidence of nosocomial respiratory tract infections in multiple trauma patients. J Trauma 1987;27:357-364

S178. Winter R, Humphreys H, Pick A, MacGowan AP, Willatts SM, Speller DC: A controlled trial of selective decontamination of the digestive tract in intensive care and its effect on nosocomial infection. J Antimicrob Chemother. 1992;30:73-87

S179. Abele-Horn M, Dauber A, Bauernfeind A, Russwurm W, Seyfarth-Metzger I, Gleich P, Ruckdeschel G: Decrease in nosocomial pneumonia in ventilated patients by selective oropharyngeal decontamination (SOD). Intensive Care Med. 1997;23:187-95.

S180. Acquarolo A, Urli T, Perone G, Giannotti C, Candiani A, Latronico N. Antibiotic prophylaxis of early onset pneumonia in critically ill comatose patients. A randomized study. Intensive Care Med. 2005; 31(4):510-6.

S181. Aerdts SJ, van Dalen R, Clasener HA, Festen J, van Lier HJ, Vollaard EJ: Antibiotic prophylaxis of respiratory tract infection in mechanically ventilated patients. A prospective, blinded, randomized trial of the effect of a novel regimen. Chest. 1991;100:783-791

S182. Bion JF, Badger I, Crosby HA, Hutchings P, Kong KL, Baker J, Hutton P, McMaster P, Buckels JA, Elliott TSJ: Selective decontamination of the digestive tract reduces gram-negative pulmonary colonization but not systemic endotoxemia in patients undergoing elective liver transplantation. Crit Care Med. 1994;22:40-49

S183. Blair P, Rowlands BJ, Lowry K, Webb H, Armstrong P, Smilie J Selective decontamination of the digestive tract: a stratified, randomized, prospective study in a mixed intensive care unit. Surgery 1991;110:303-309

S184. Cockerill FR, 3rd, Muller SR, Anhalt JP, et al. Prevention of infection in critically ill patients by selective decontamination of the digestive tract. *Ann Intern Med* 1992;117:545-53.

S185. de La Cal MA, Cerdá E, Garcıa-Hierro P, Van Saene HK. G ómez-Santos D, Negro E & Lorente JA. Survival benefit in critically ill burned patients receiving selective decontamination of the digestive tract: a randomized, placebocontrolled, double-blind trial. Ann Surg. 2005;241:424-30.

S186. Ferrer M, Torres A, Gonzalez J, Puig de la Bellacasa J, el-Ebiary M, Roca M, Gatell JM, Rodriguez-Roisin R: Utility of selective digestive decontamination in mechanically ventilated patients. Ann Intern Med. 1994;120:389-395

S187. Georges B, Mazerolles M, Decun J-F, Rouge P, Pomies S, Cougot P Décontamination digestive sélective résultats d'une étude chez le polytraumatisé. *Réanimation Soins Intensifs Médecin d'Urgence* 1994;3:621-627

S188. Hammond JM, Potgieter PD. Neurologic disease requiring long-term ventilation: the role of selective decontamination of the digestive tract in preventing nosocomial infection. *Chest*. 1993; 104(2):547-51.

S189. Hammond JM, Potgieter PD, Saunders LG. Selective decontamination of the digestive tract in multiple trauma patients-Is there a role? Results of a prospective, double-blind, randomized trial. Crit Care Med. 1994;22(1):33-9.

S190. Jacobs S, Foweraker JE, Roberts SE: Effectiveness of selective decontamination of the digestive tract (SDD) in an ICU with a policy encouraging a low gastric pH. Clin Intensive Med. 1992;3:52-58

S191. Karvouniaris M, Makris D, Zygoulis P, Triantaris A, Xitsas S, Mantzarlis K, Petinaki E, Zakynthinos E. Nebulised colistin for ventilator-associated pneumonia prevention. Eur Resp J. 2015:46:1544-1547.

S192. Korinek AM, Laisne MJ, Nicolas MH, Raskine L, Deroin V, Sanson-lepors MJ: Selective decontamination of the digestive tract in neurosurgical intensive care unit patients: a double-blind, randomized, placebo-controlled study. Crit Care Med. 1993;21:1466-73.

S193. Laggner AN, Tryba M, Georgopoulos A, Lenz K, Grimm G, Graninger W, Schneeweiss B, Druml W: Oropharyngeal decontamination with gentamicin for long-term ventilated patients on stress ulcer prophylaxis with sucralfate? *Wien Klin Wochenschr* 1994, 106:15-19.

S194. Palomar M, Alvarez-Lerma F, Jorda R, Bermejo B, Catalan Study Group of Nosocomial Pneumonia Prevention: Prevention of nosocomial infection in mechanically ventilated patients: selective digestive decontamination versus sucralfate. Clin Intens Care. 1997;8:228-235

S195. Pneumatikos I, Koulouras V, Nathanail C, Goe D, Nakos G: Selective decontamination of subglottic area in mechanically ventilated patients with multiple trauma. Intensive Care Med. 2002;28:432-437

S196. Quinio B, Albanese J, Bues-Charbit M, Viviand X, Martin C; Selective decontamination of the digestive tract in multiple trauma patients. A prospective double-blind, randomized, placebo-controlled study. Chest 1996;109:765-772

S197. Rocha LA, Martin MJ, Pita S, Paz J, Seco C, Margusino L, Villanueva R, Duran MT: Prevention of nosocomial infection in critically ill patients by selective decontamination of the digestive tract. A randomized, double blind, placebo-controlled study. Intensive Care Med. 1992;18:398-404

S198. Rodríguez-Roldán JM, Altuna-Cuesta A, López A, Carrillo A, Garcia J, León J, Martínez-Pellús AJ: Prevention of nosocomial lung infection in ventilated patients: use of an antimicrobial pharyngeal nonabsorbable paste. Crit Care Med. 1990;18:1239-42

S199. Rolando N, Gimson A, Wade J, Philpott- Howard J, Casewell M, Williams R: Prospective controlled trial of selective parenteral and enteral antimicrobial regimen in fulminant liver failure. Hepatol. 1993;17:196-201

S200. Rolando N, Wade JJ, Stangou A, Gimson AE, Wendon J, Philpott- Howard J, Williams R. Prospective study comparing the efficacy of prophylactic parenteral antimicrobials, with or without enteral decontamination, in patients with acute liver failure. Liver transplantation and surgery. 1996; 2:8-13

S201. Sirvent JM, Torres A, El-Ebiary M, Castro P, de Batlle J, Bonet A. Protective effect of intravenously administered cefuroxime against nosocomial pneumonia in patients with structural coma. *Am J Respir Crit Care Med* 1997;155:1729-1734

S202. Smith SD, Jackson RJ, Hannakan CJ, Wadowsky RM, Tzakis AG, Rowe MI; Selective decontamination in pediatric liver transplants. Transplantation 1993;55:1306-1308

S203. Stoutenbeek CP, van Saene HKF, Little RA, Whitehead A: The effect of selective decontamination of the digestive tract on mortality in multiple trauma patients: a multicenter randomized controlled trial. Intensive Care Med. 2007;33:261-270

S204. Unertl K, Ruckdeschel G, Selbmann HK, et al; Prevention of colonization and respiratory infections in long-term ventilated patients by local antimicrobial prophylaxis. Intensive Care Med 1987;13:106-113

S205. Verwaest C, Verhaegen J, Ferdinande P, Schetz M, Van den Berghe G, Verbist L, Lauwers P: Randomized, controlled trial of selective digestive decontamination in 600 mechanically ventilated patients in a multidisciplinary intensive care unit. Crit Care Med. 1997;25:63-71

S206. Wiener J, Itokazu G, Nathan C, Kabins SA, Weinstein RA: A randomized, double-blind, placebo-controlled trial of selective digestive decontamination in a medical-surgical intensive care unit. Clin Infect Dis. 1995;20:861-867

S207. Frencken JF, Wittekamp BH, Plantinga NL, Spitoni C, van de Groep K, Cremer OL, Bonten MJ. Associations Between Enteral Colonization With Gram-Negative Bacteria and Intensive Care Unit–Acquired Infections and Colonization of the Respiratory Tract. *Clin Infect Dis*. 2017;66(4):497-503.

S208. Garbino J, Lew DP, Romand JA, Hugonnet S, Auckenthaler R, Pittet D: Prevention of severe Candida infections in nonneutropenic, high-risk, critically ill patients: a randomized, double-blind,

placebo-controlled trial in patients treated by selective digestive decontamination. Intensive Care Med 2002;28:1708-1717

S209. Leone M, Bourgoin A, Giuly E, et al. Influence on outcome of ventilator-associated pneumonia in multiple trauma patients with head trauma treated with selected digestive decontamination. *Crit Care Med* 2002; 30:1741-6.

S210. Nardi G, Di Silvestre A, De Monte A, Massarutti D, Proietti A, Troncon MG, Zussino M: Reduction in gram-positive pneumonia and antibiotic consumption following the use of a SDD protocol including nasal and oral mupirocin. Eur J Emerg Med 2001;8:203-214

S211. Oostdijk EAN, Kesecioglu J, Schultz MJ, et al. Notice of Retraction and Replacement: Oostdijk et al. Effects of Decontamination of the Oropharynx and Intestinal Tract on Antibiotic Resistance in ICUs: A Randomized Clinical Trial. *JAMA*. 2014;312(14):1429-1437. *JAMA* 2017

S212. Rouby JJ, Poete P, de Lassale EM, Nicolas MH, Bodin L, Jarlier V, Korinek AM, Viars P. Prevention of Gram negative noscomial bronchopneumonia by intratracheal colistin in critically ill patients. Intensive Care Med. 1994;20(3):187-92.

S213. Silvestri L, Bragadin CM, Milanese M, Gregori D, Consales C, Gullo A, Van Saene HK. Are most ICU infections really nosocomial? A prospective observational cohort study in mechanically ventilated patients. *J Hosp Infect.* 1999;42(2):125-33.

S214. Steffen R, Reinhartz O, Blumhardt G, Bechstein WO, Raakow R, Langrehr JM, Rossaint R, Slama K, Neuhaus P. Bacterial and fungal colonization and infections using oral selective bowel decontamination in orthotopic liver transplantations. *Transpl Inter.* 1994;7(2):101-8.

S215. Verhaegen J: Randomized study of selective digestive decontamination on colonization and prevention of infection in mechanically ventilated patients in the ICU. 1992. Doctor in Medical Sciences – thesis, University Hospital, Leuven, Belgium.

Fig S1. (p 36) Search method, screening criteria and resulting classification of eligible studies and subsequent decant of component groups. The four numbered arrows are as follows;

(1) An electronic search for systematic reviews or meta-analysis (SR/MA) containing potentially eligible studies using search terms; “ventilator associated pneumonia”, “mechanical ventilation”, “intensive care unit”, each combined with either “meta-analysis” or “systematic review” up to December 2018;

(2) The systematic reviews were then searched for studies of patient populations requiring prolonged (> 24 hours) ICU admission

(3) The studies were triaged from the systematic reviews into one of four categories; studies in which there was no intervention (observational studies), studies of various non-decontamination methods such as methods delivered either via the gastric route, the airway route or via the oral care route, studies of anti-septic methods and studies with a TAP (in any formulation) based intervention.

(4) All studies were reviewed for potentially eligible studies and screened against inclusion and exclusion criteria. Any duplicate or ineligible studies were removed and

(5) Studies identified outside of systematic reviews were included;

(6) The component groups were decanted from each study being control (rectangles), intervention (ovals) and observation (diamond) groups.

Note; the total numbers do not tally as some systematic reviews provided studies in more than one category and some studies provided groups in more than one category and some studies have unequal numbers of control and interventions groups.

TAP = Topical antibiotic prophylaxis

Figure 1: Flow chart of literature search and study and group decant

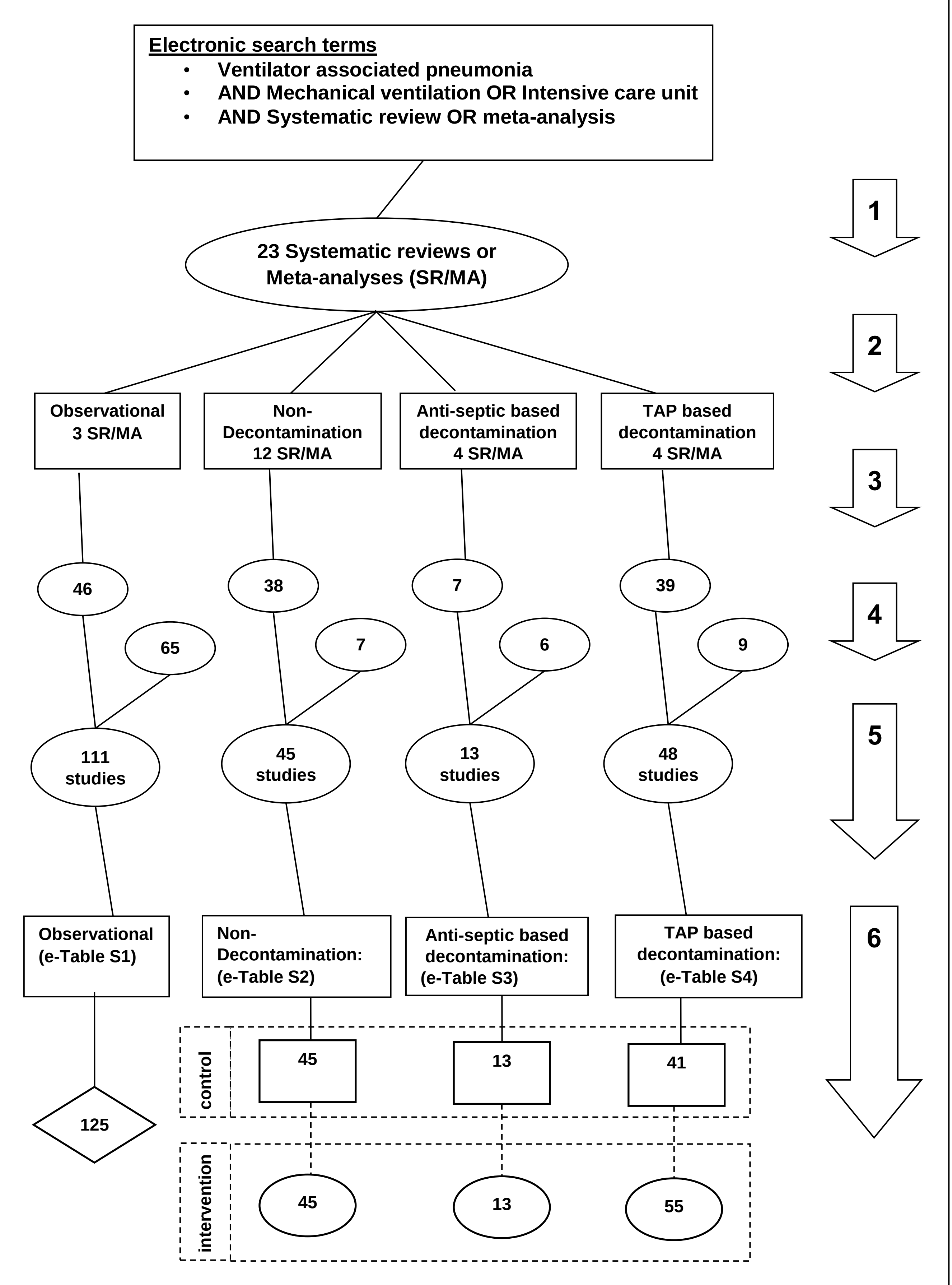

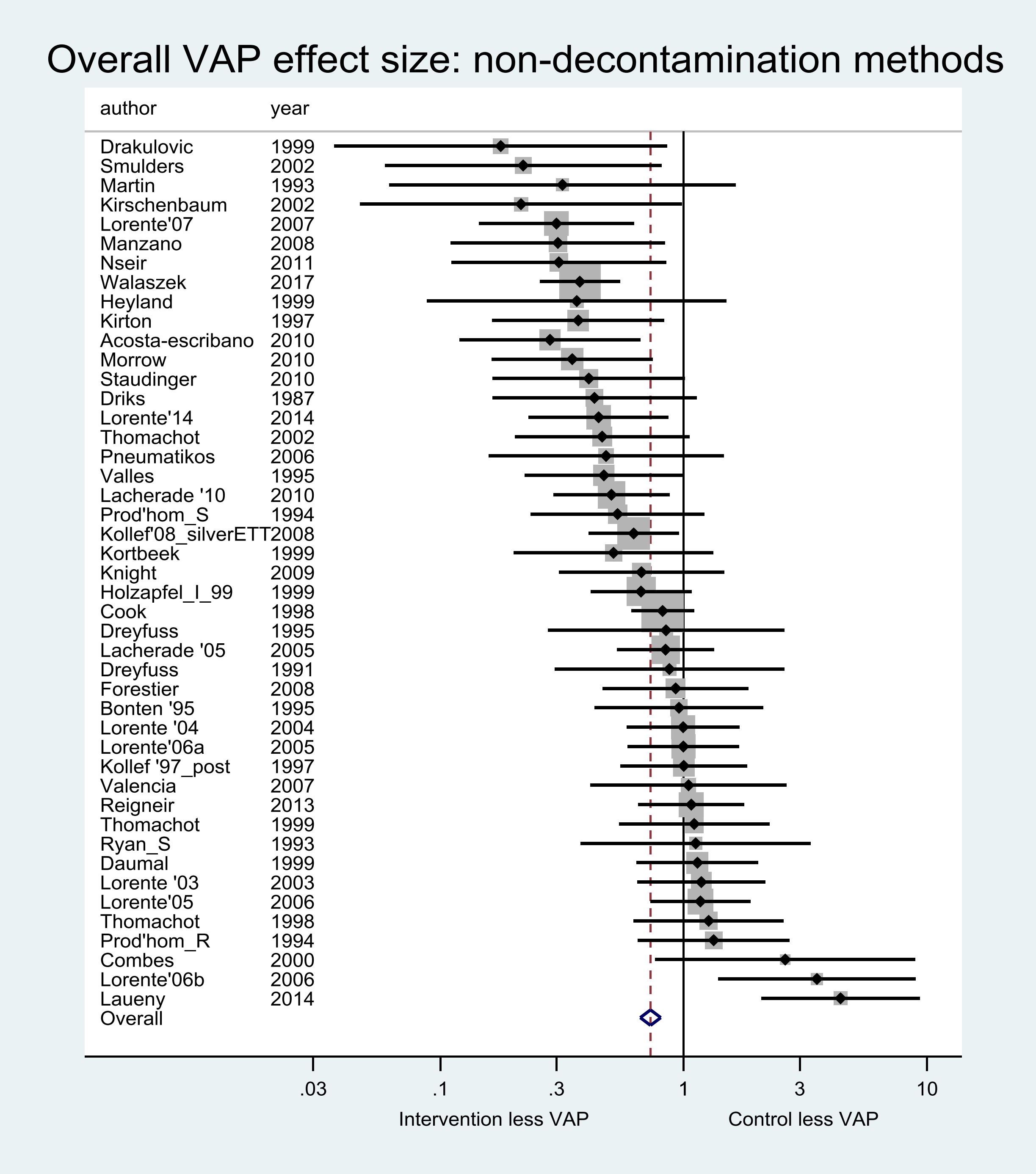

Fig S2

Caterpillar plots of the group specific (small squares) and summary (large open diamond, broken vertical line) effect size on the overall VAP incidence and 95 % CI among studies of non-decontamination methods of VAP prevention. Studies are listed in Table S2.

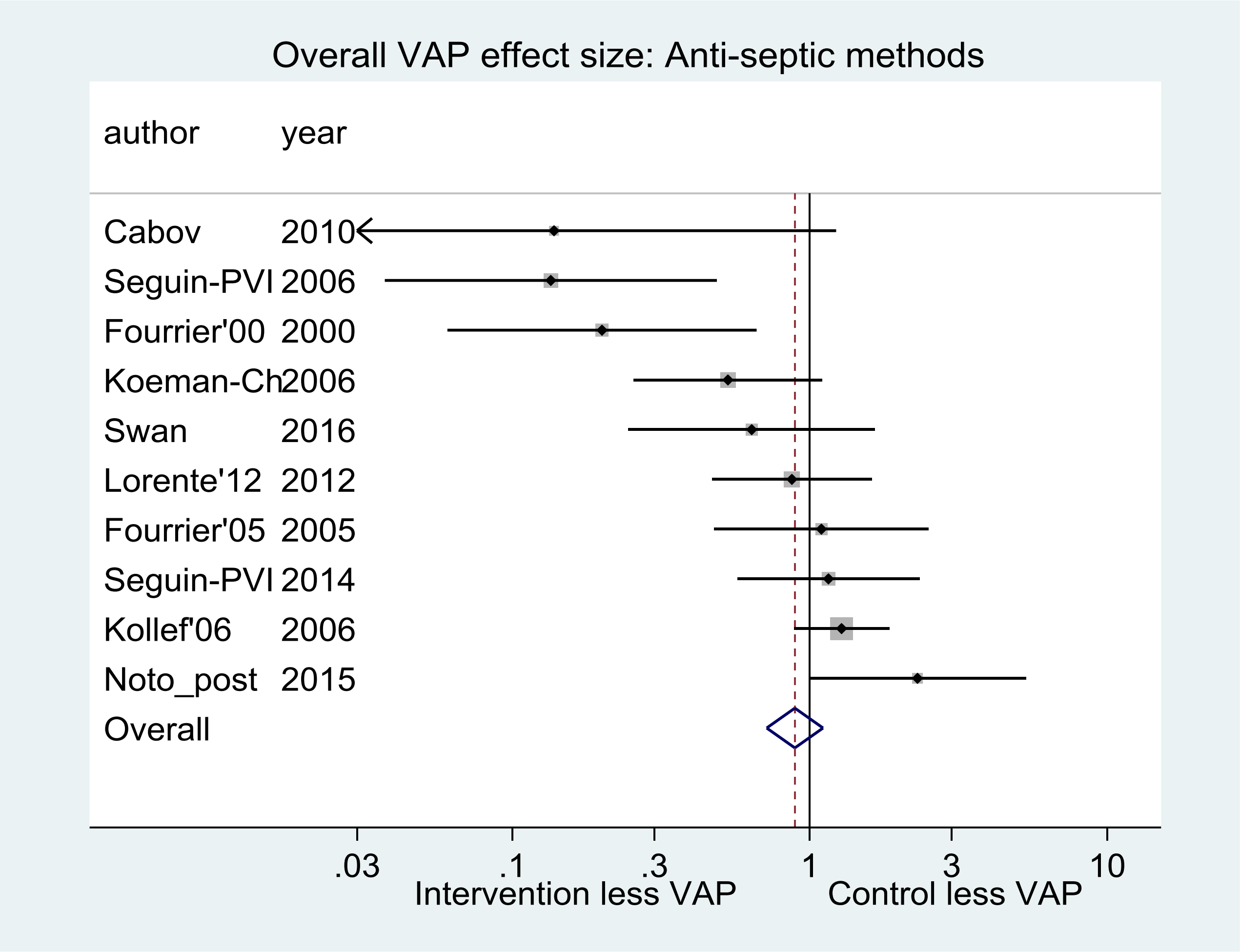

Fig S3

Caterpillar plots of the group specific (small squares) and summary (large open diamond, broken vertical line) effect size on the overall VAP incidence and 95 % CI among studies of anti-septic based methods of VAP prevention. Studies are listed in Table S3.

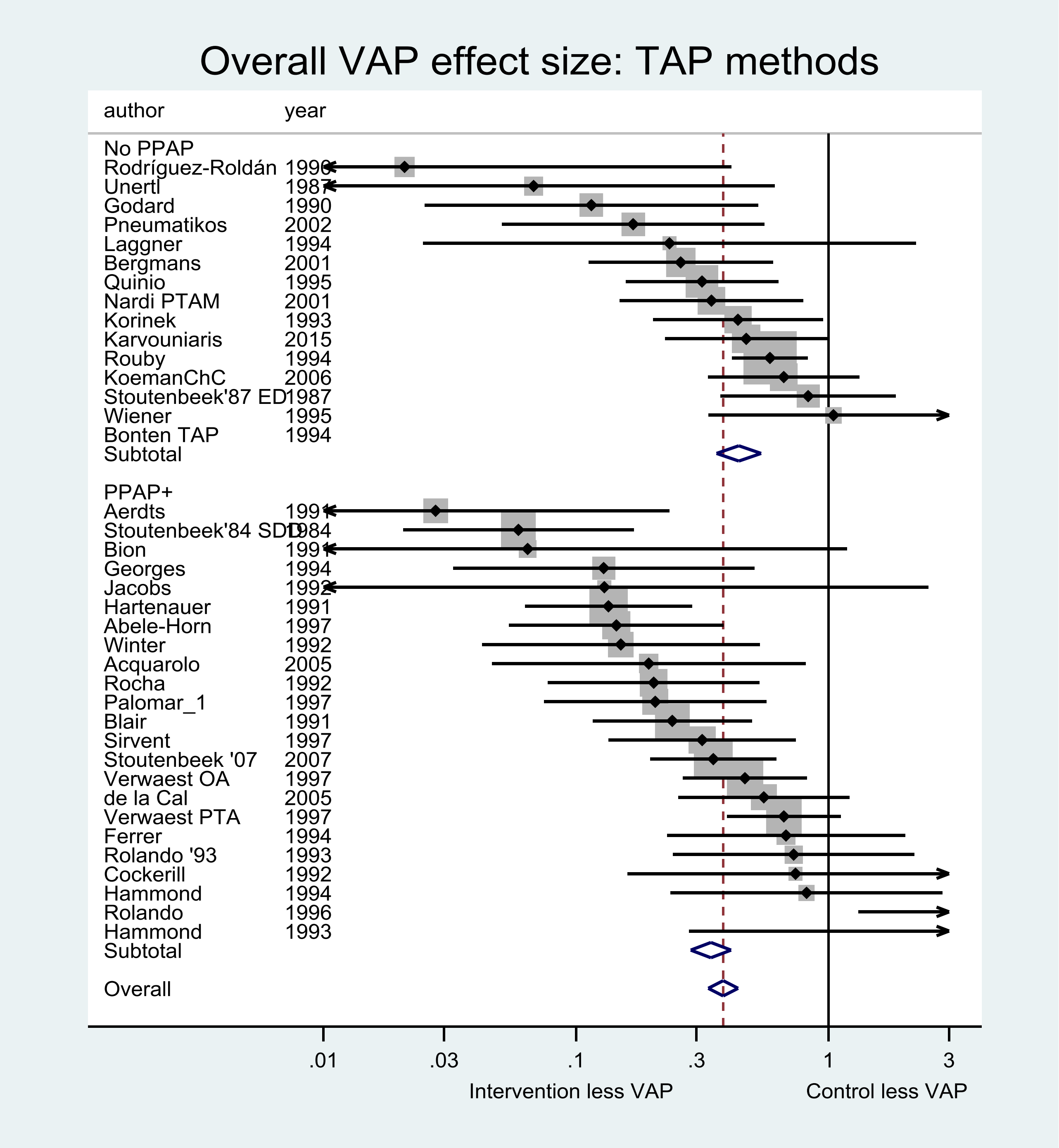

Fig S4

Caterpillar plots of the group specific (small squares) and summary (large open diamond, broken vertical line) effect size on the overall VAP incidence and 95 % CI among studies of TAP ±PPAP based methods of VAP prevention. TAP = Topical antibiotic prophylaxis. Studies are listed in Table S4.

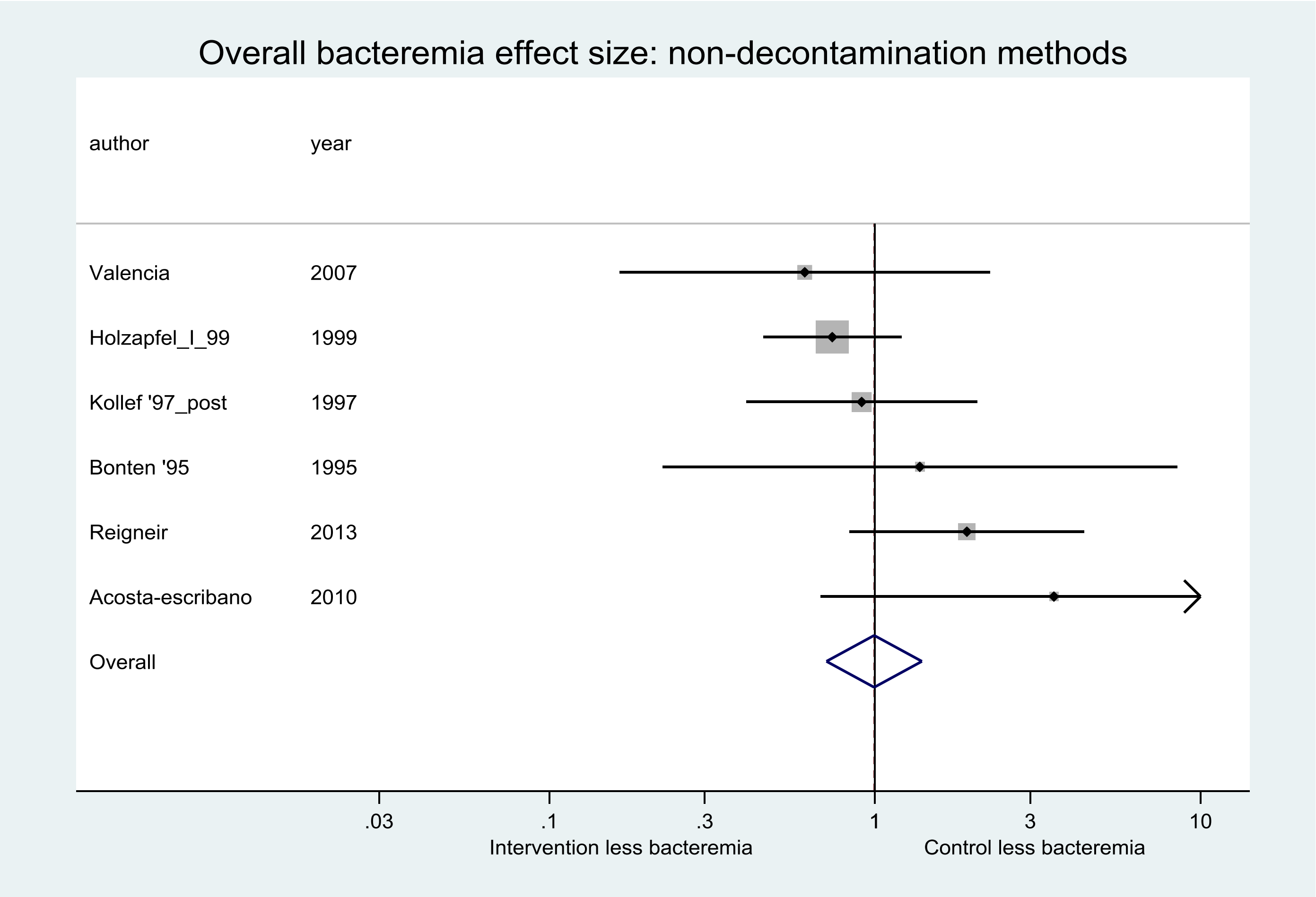

Fig S5

Caterpillar plots of the group specific (small squares) and summary (large open diamond, broken vertical line) effect size on the overall bacteremia incidence and 95 % CI among studies of non-decontamination methods of VAP prevention. Studies are listed in Table S2.

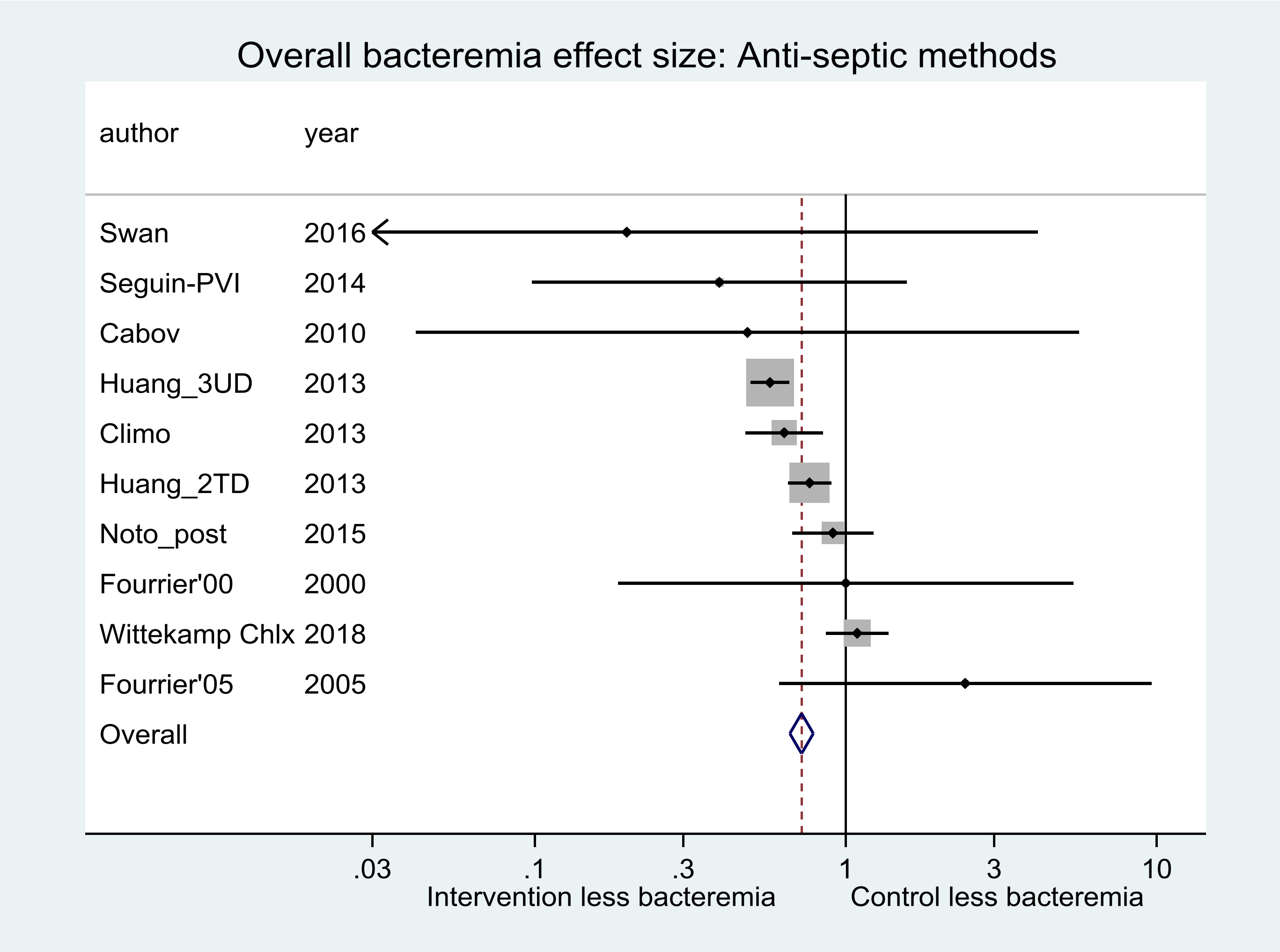

Fig S6

Caterpillar plots of the group specific (small squares) and summary (large open diamond, broken vertical line) effect size on the overall bacteremia incidence and 95 % CI among studies of anti-septic based methods of VAP prevention. Studies are listed in Table S3.

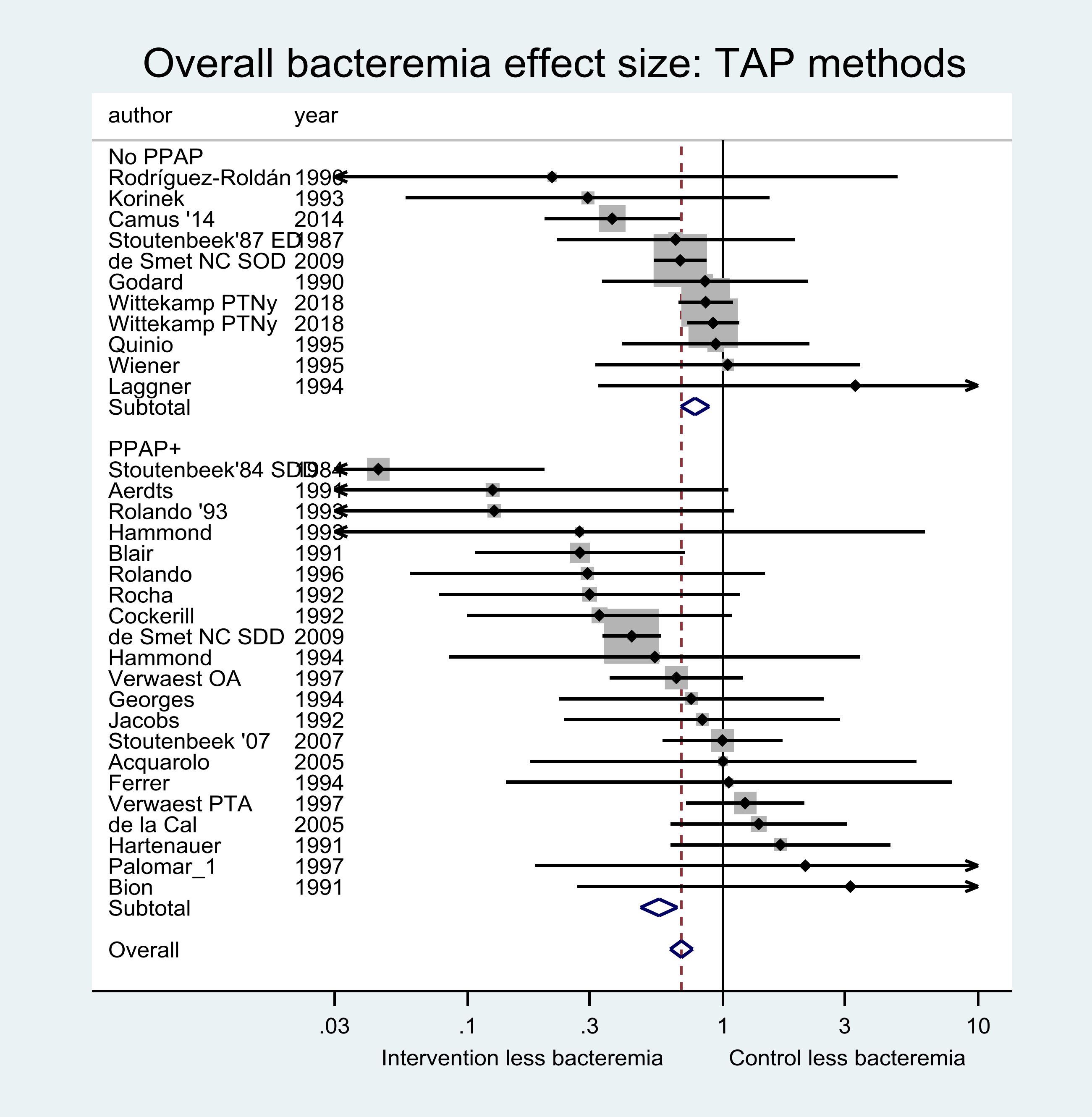

Fig S7

Caterpillar plots of the group specific (small squares) and summary (large open diamond, broken vertical line) effect size on the overall bacteremia incidence and 95 % CI among studies of TAP ±PPAP based methods of VAP prevention. TAP = Topical antibiotic prophylaxis. Studies are listed in Table S4.

Fig S8

GSEM model 1

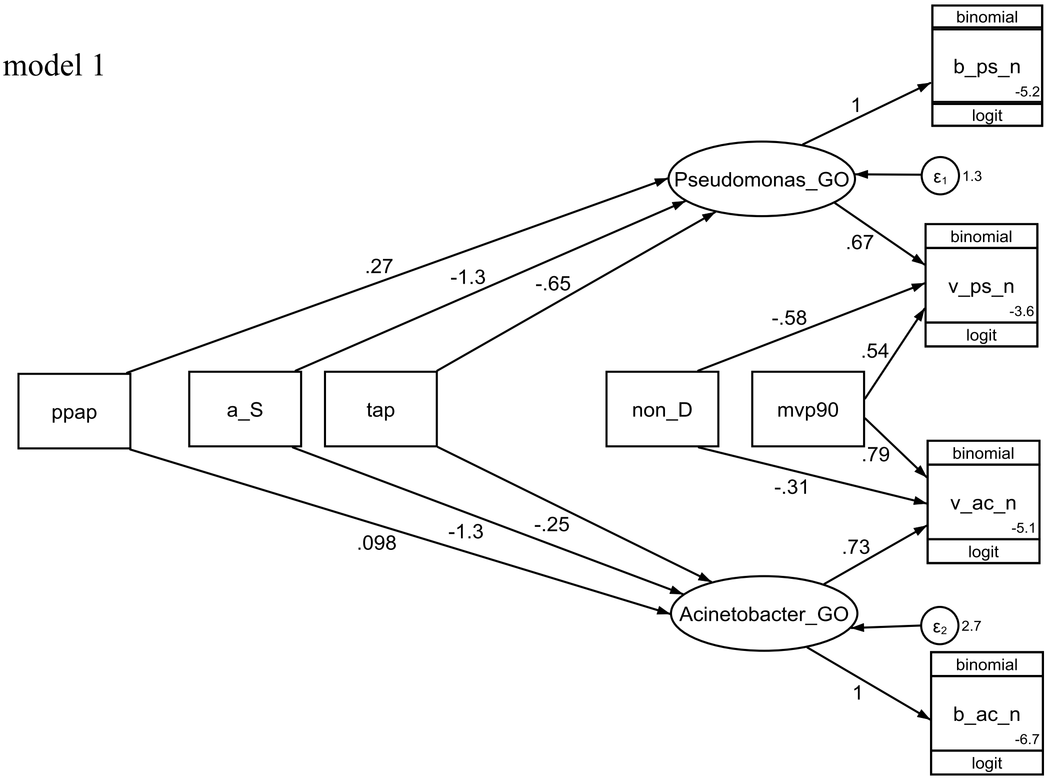

Fig S9

GSEM model 2

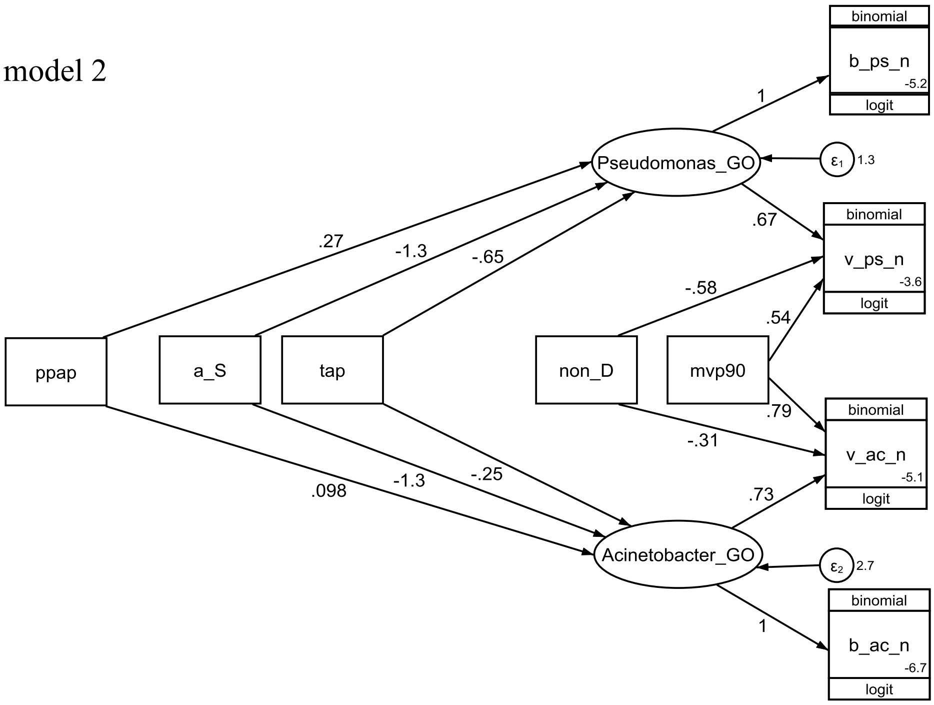

Fig S10

GSEM model 3

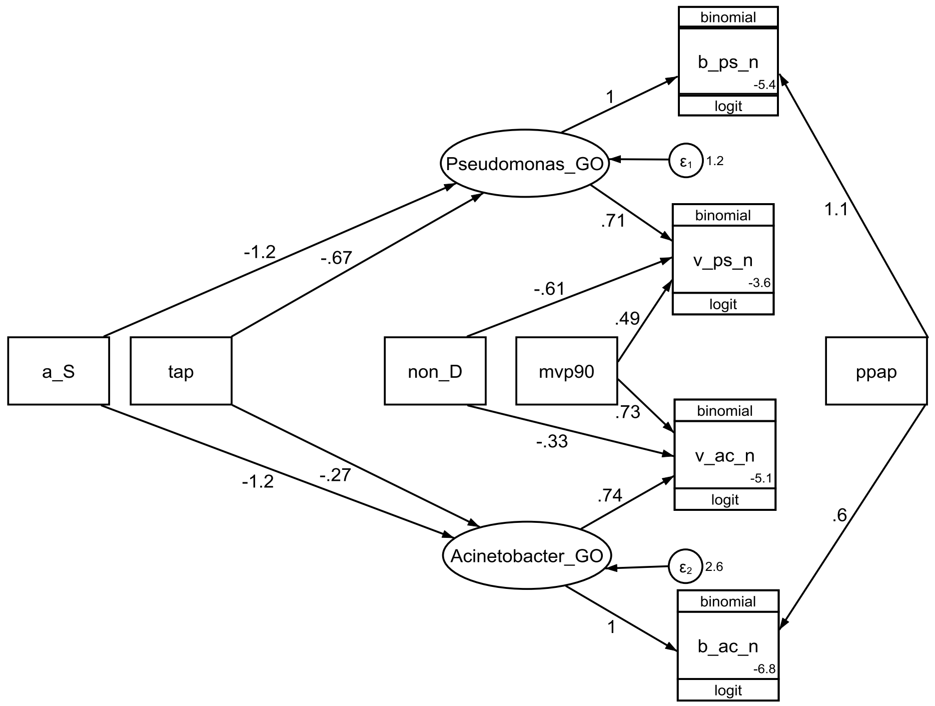

Fig S811

GSEM model 4

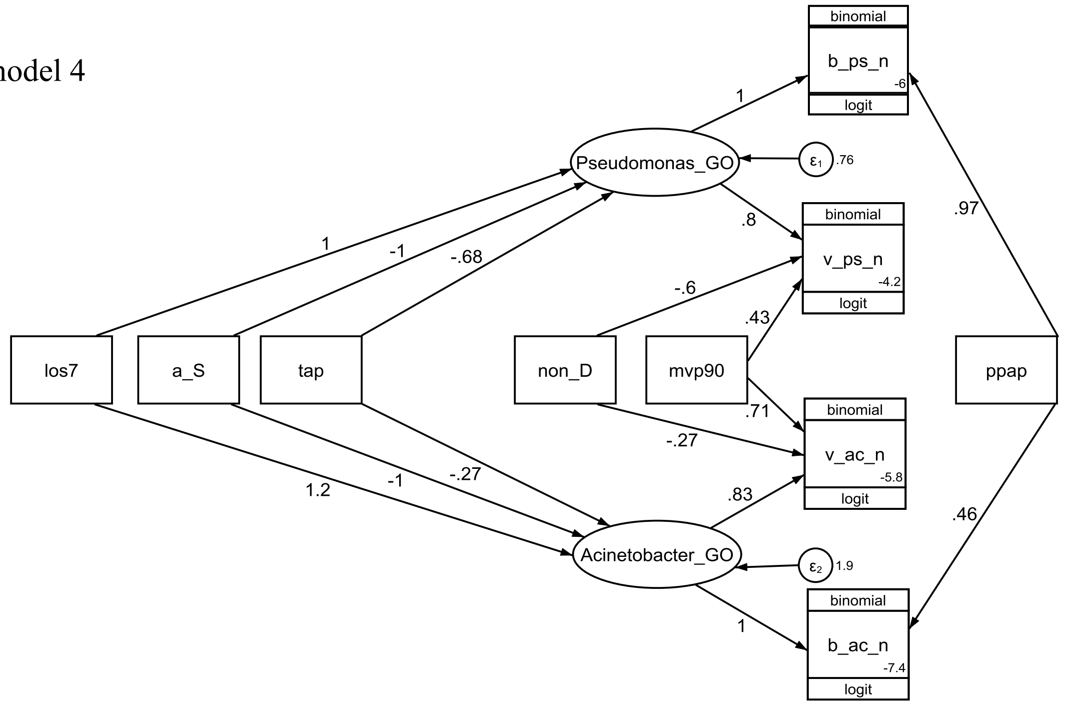

Fig S12

GSEM model 5

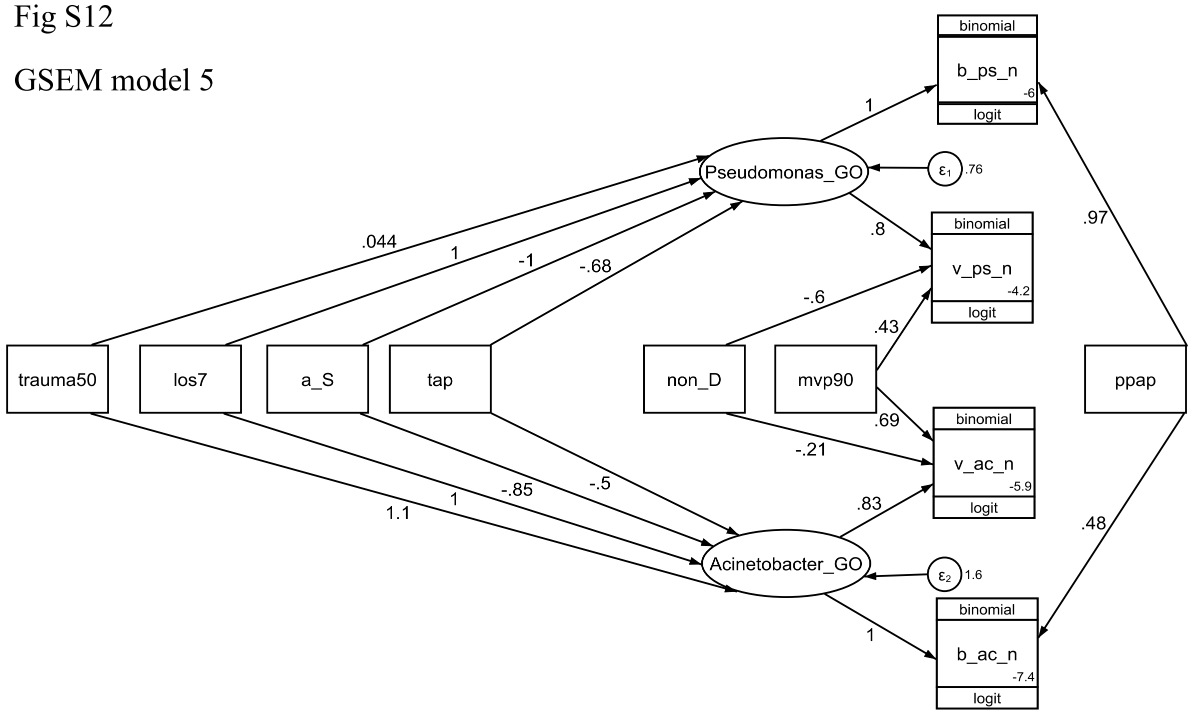

Fig S13

GSEM model 6

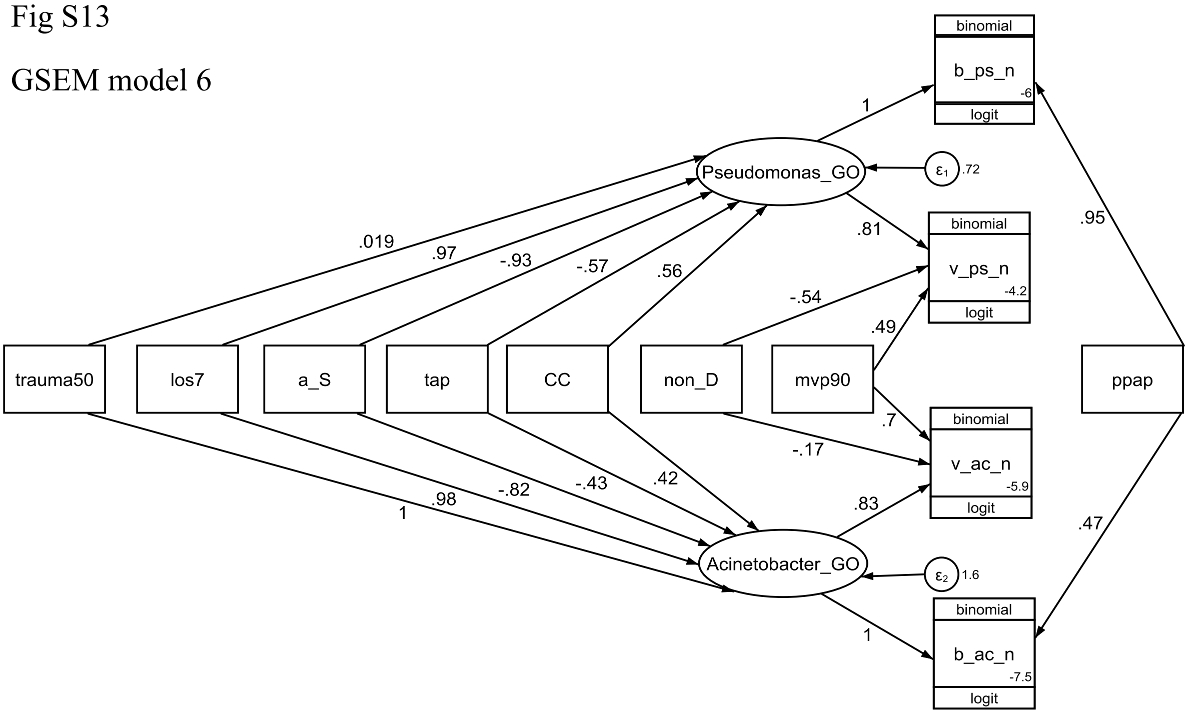

Fig S14

GSEM model 7

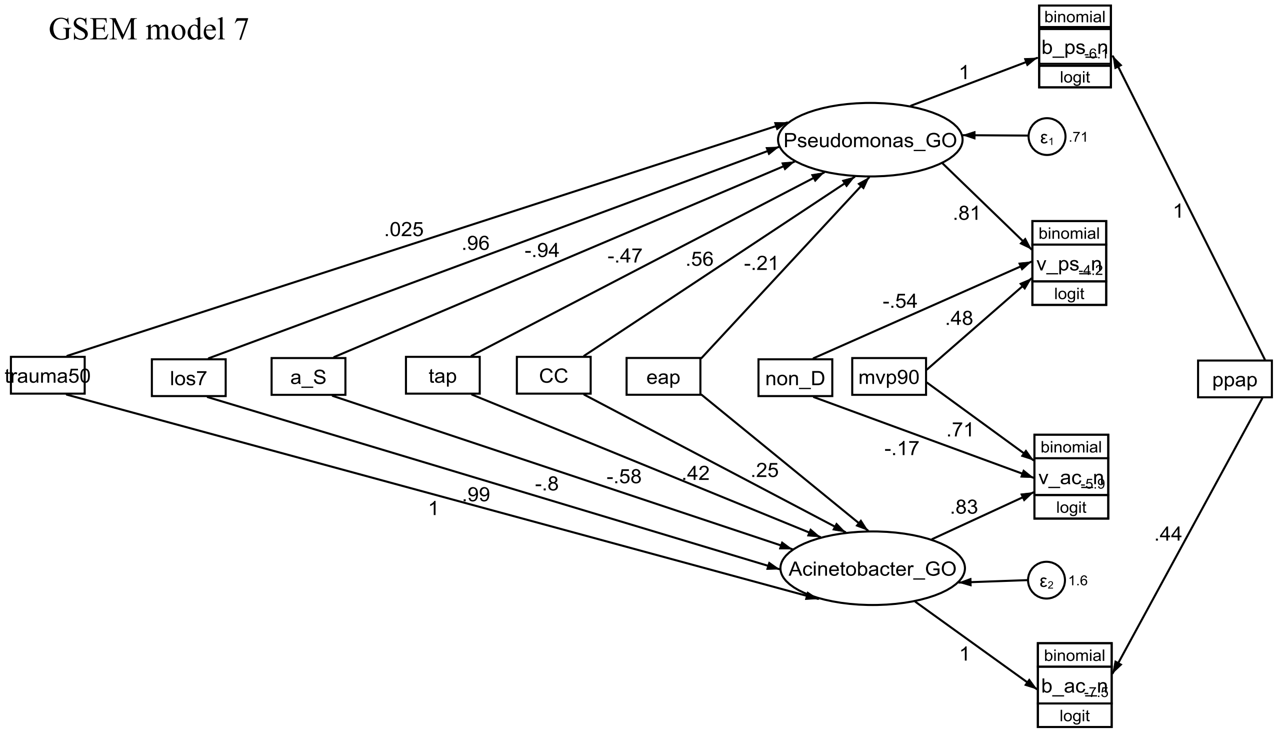

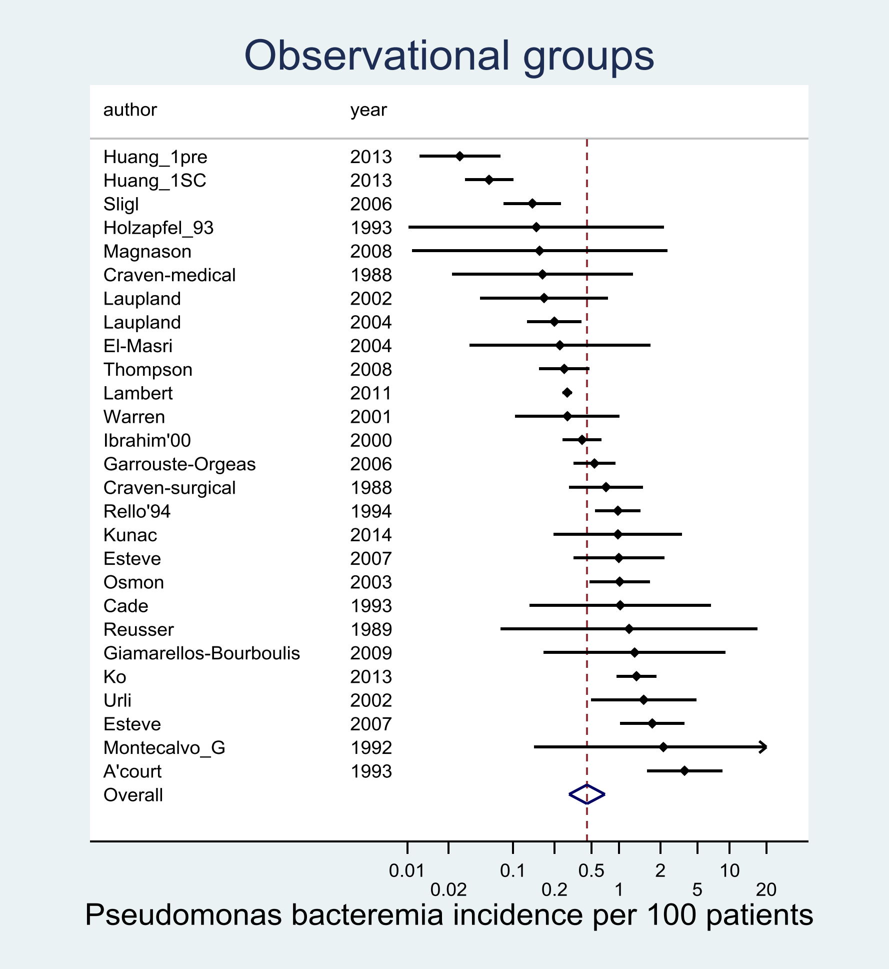

Fig S15

Pseudomonas bacteremia incidence among observational studies. Caterpillar plots of the group specific (small squares) and summary (large open diamonds) *Pseudomonas* bacteremia incidence proportion and 95 % CI. Groups are listed in Table S1. Note that the x axis is a logit scale and that groups with a zero event have a continuity correction (N+0.5) to enable them to appear in the plot. These zero count groups contribute < 1.5% in weight each towards the summary estimate. The central solid line is the *Pseudomonas* bacteremia benchmark.

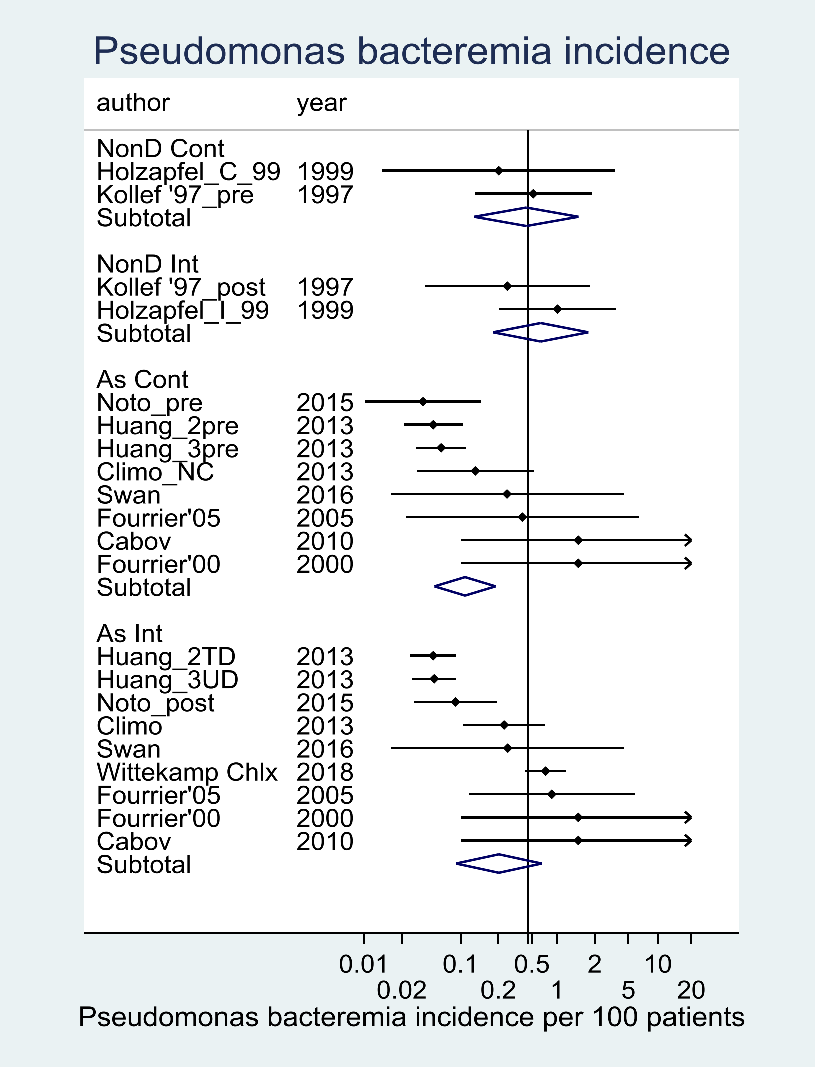

Fig S16

Pseudomonas bacteremia incidence among control and intervention groups of studies of non-decontamination methods & studies of anti-septic methods of infection prevention. Caterpillar plots of the group specific (small squares) and summary (large open diamonds) *Pseudomonas* bacteremia incidence proportion and 95 % CI. Groups are listed in Tables S2 & S3. Note that the x axis is a logit scale and that groups with a zero event have a continuity correction (N+0.5) to enable them to appear in the plot. These zero count groups contribute < 6% in weight each towards the summary estimate. The central solid line is the *Pseudomonas* bacteremia benchmark derived from Fig S15.

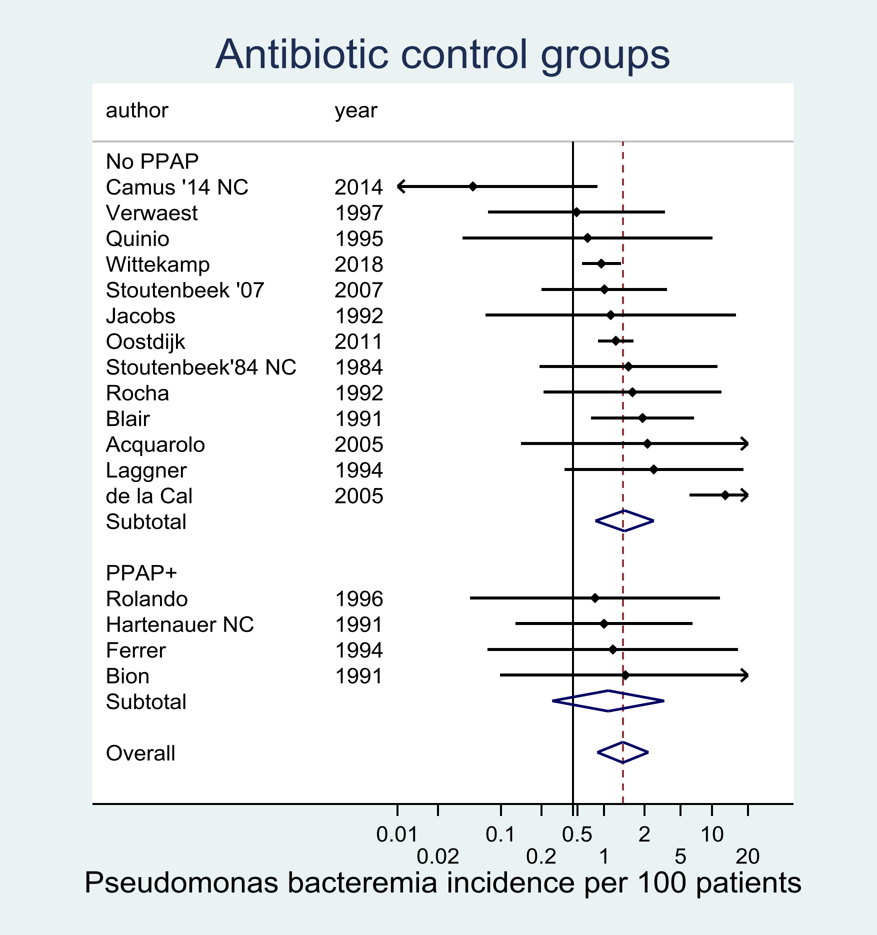

Fig S17

Pseudomonas bacteremia incidence among control groups of studies of TAP methods of infection prevention stratified by use of PPAP or not. Caterpillar plots of the group specific (small squares) and summary (large open diamonds) *Pseudomonas* bacteremia incidence proportion and 95 % CI. Groups are listed in Tables S4. Note that the x axis is a logit scale and that groups with a zero event have a continuity correction (N+0.5) to enable them to appear in the plot. These zero count groups contribute < 4% in weight each towards the summary estimate. The central solid line is the *Pseudomonas* bacteremia benchmark derived from Fig S15.

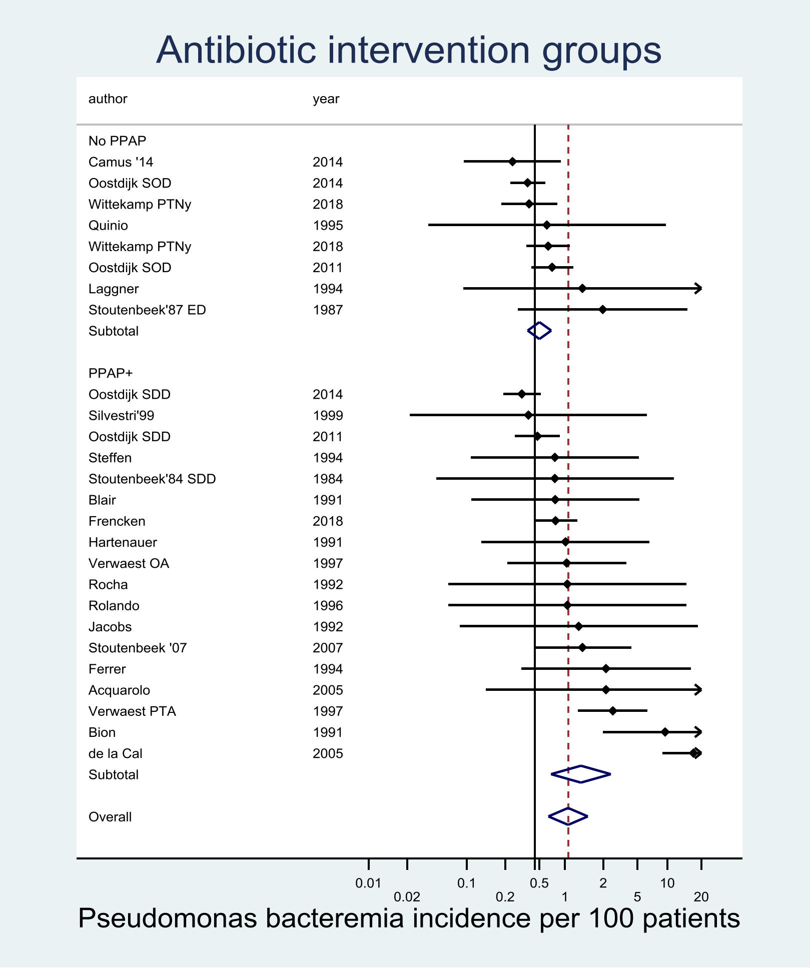

Fig S18

Pseudomonas bacteremia incidence among intervention groups of studies of topical antibiotic methods of infection prevention stratified by use of PPAP or not. Caterpillar plots of the group specific (small squares) and summary (large open diamonds) *Pseudomonas* bacteremia incidence proportion and 95 % CI. Groups are listed in Tables S4. Note that the x axis is a logit scale and that groups with a zero event have a continuity correction (N+0.5) to enable them to appear in the plot. These zero count groups contribute < 2% in weight each towards the summary estimate. The central solid line is the *Pseudomonas* bacteremia benchmark derived from Fig S15.

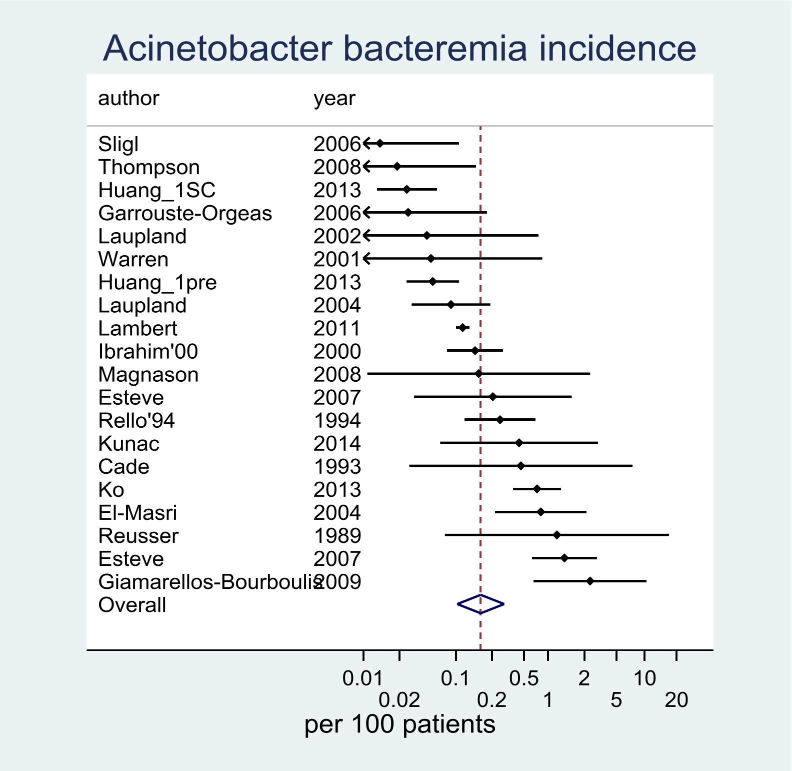

Fig S19

Acinetobacter bacteremia incidence among observational studies. Caterpillar plots of the group specific (small squares) and summary (large open diamonds) *Acinetobacter* bacteremia incidence proportion and 95 % CI. Groups are listed in Table S1. Note that the x axis is a logit scale and that groups with a zero event have a continuity correction (N+0.5) to enable them to appear in the plot. These zero count groups contribute < 3% in weight each towards the summary estimate. The central solid line is the *Acinetobacter* bacteremia benchmark.

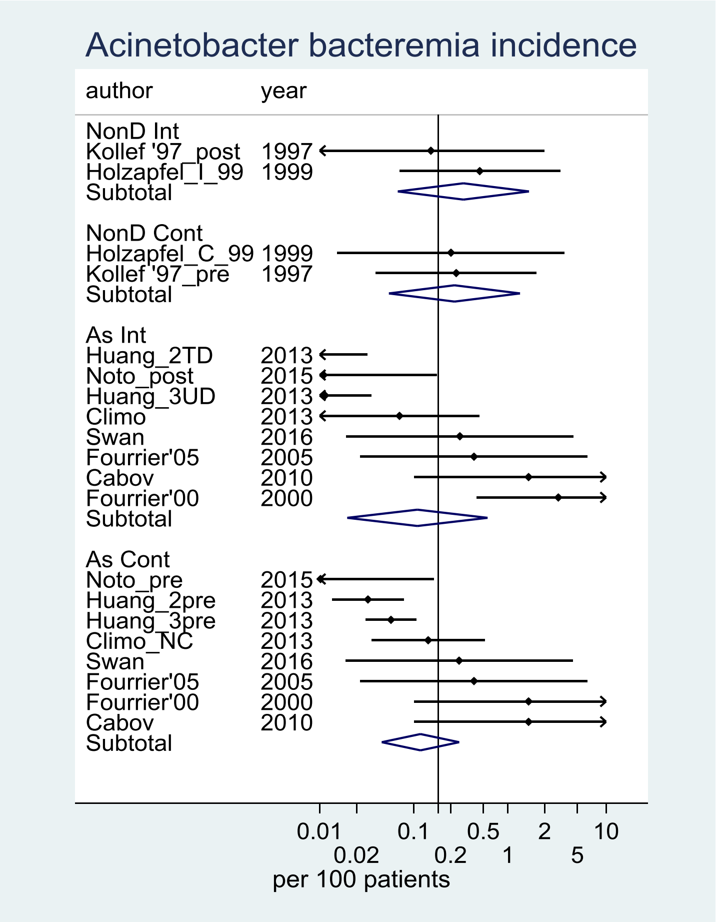

Fig S20

Acinetobacter bacteremia incidence among control and intervention groups of studies of non-decontamination methods & studies of anti-septic methods of infection prevention. Caterpillar plots of the group specific (small squares) and summary (large open diamonds) *Acinetobacter* bacteremia incidence proportion and 95 % CI. Groups are listed in Tables S2 & S3. Note that the x axis is a logit scale and that groups with a zero event have a continuity correction (N+0.5) to enable them to appear in the plot. These zero count groups contribute < 4% in weight each towards the summary estimate. The central solid line is the *Acinetobacter* bacteremia benchmark derived from Fig S19.

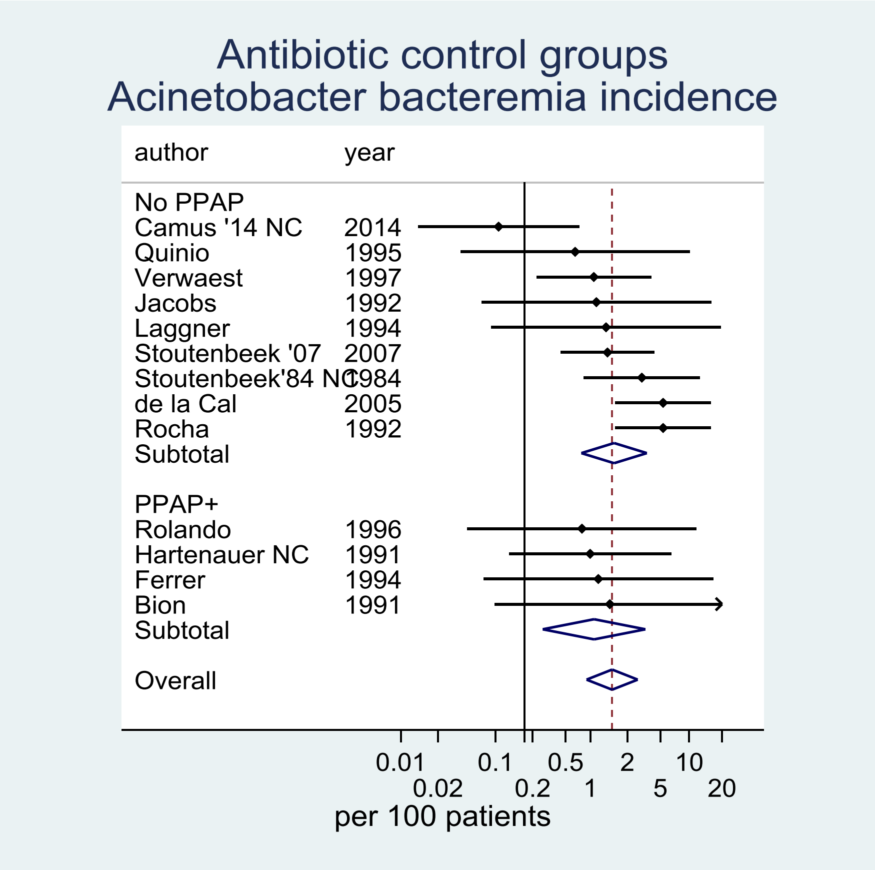

Fig S21

Acinetobacter bacteremia incidence among control groups of studies of topical antibiotic methods of infection prevention stratified by use of PPAP or not. Caterpillar plots of the group specific (small squares) and summary (large open diamonds) *Acinetobacter* bacteremia incidence proportion and 95 % CI. Groups are listed in Tables S4. Note that the x axis is a logit scale and that groups with a zero event have a continuity correction (N+0.5) to enable them to appear in the plot. These zero count groups contribute < 4.5% in weight each towards the summary estimate. The central solid line is the *Acinetobacter* bacteremia benchmark derived from Fig S19.

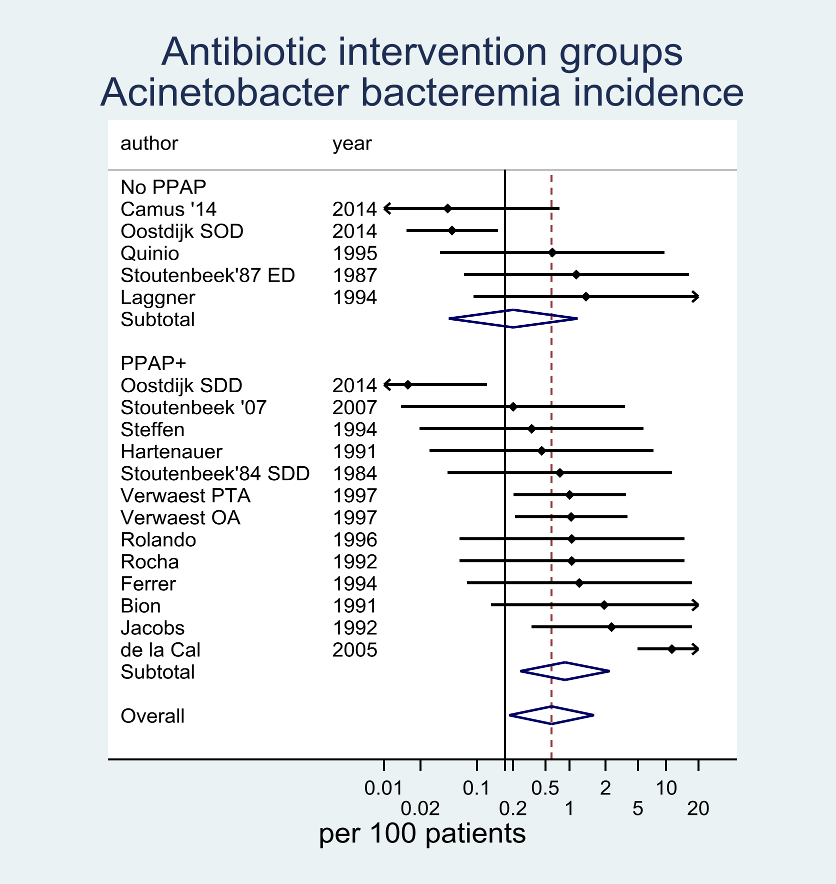

Fig S22

Acinetobacter bacteremia incidence among intervention groups of studies of topical antibiotic methods of infection prevention stratified by use of PPAP or not. Caterpillar plots of the group specific (small squares) and summary (large open diamonds) *Acinetobacter* bacteremia incidence proportion and 95 % CI. Groups are listed in Tables S4. Note that the x axis is a logit scale and that groups with a zero event have a continuity correction (N+0.5) to enable them to appear in the plot. These zero count groups contribute < 5% in weight each towards the summary estimate. The central solid line is the *Acinetobacter* bacteremia benchmark derived from Fig S18.
